# Supplementary figures and images for: Tomosyn affects dense core vesicle composition but not exocytosis in mammalian neurons
Source: eLife. 2023 Sep 11;12:e85561. doi: 10.7554/eLife.85561 (PMC10495110; doi:10.7554/eLife.85561)

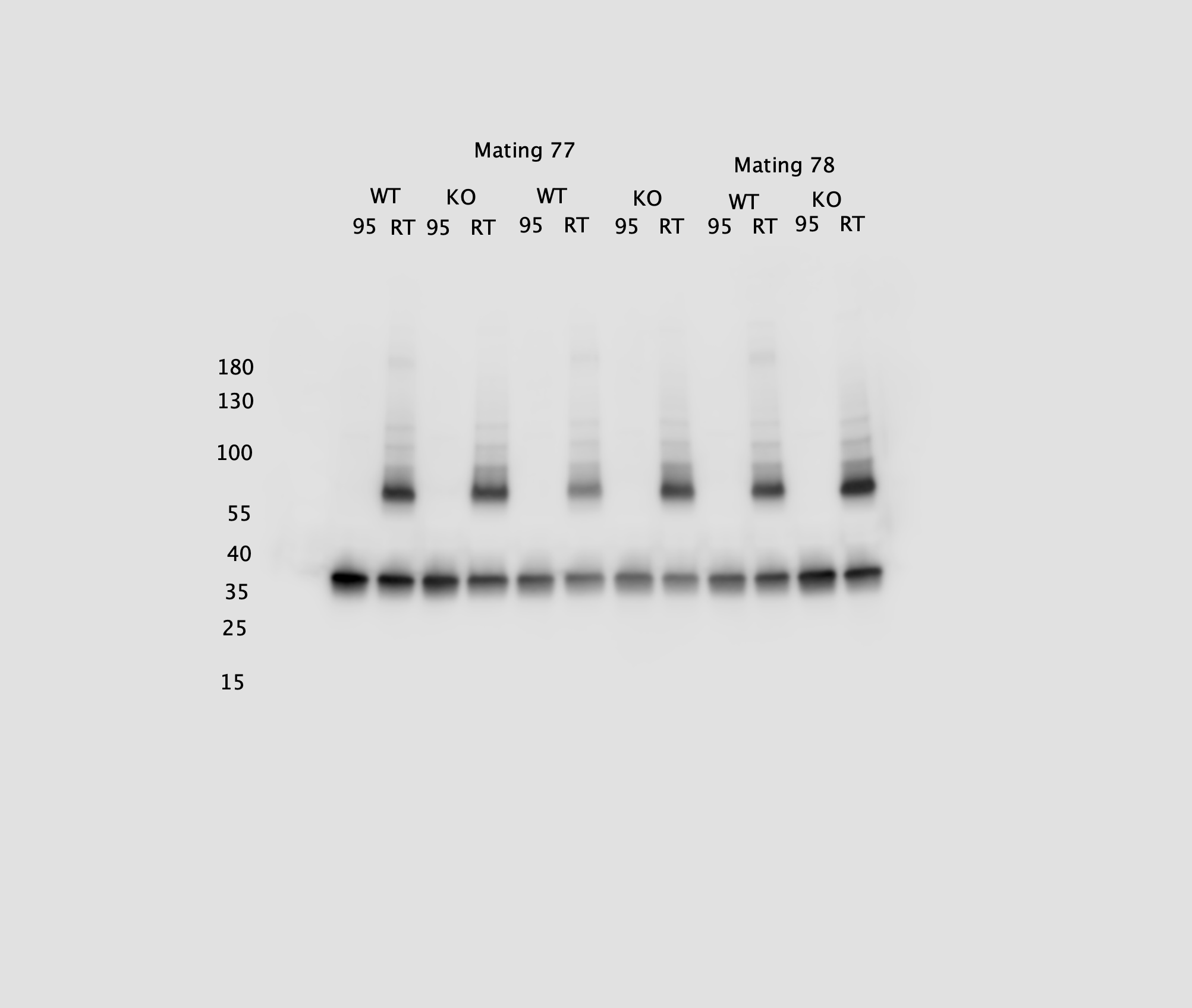

Supplement: Figure 1—source data 1. [file elife-85561-fig1-data1.zip › Figure 1_source files/Syntaxin_replicates3-4.tif]

**Culture #1**

Syntaxin 1

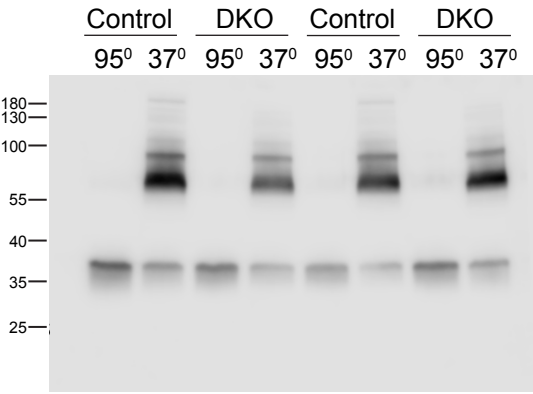

**Culture #2**

Syntaxin 1

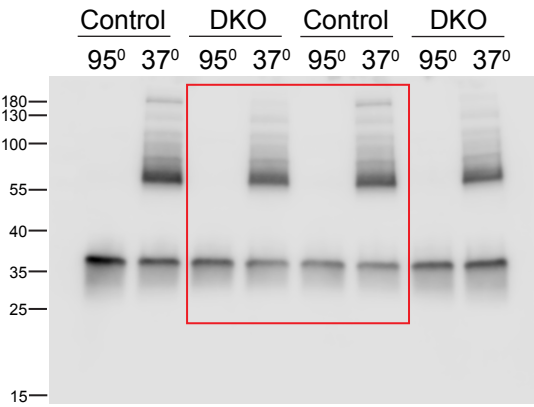

Tomosyn (STXBP5)

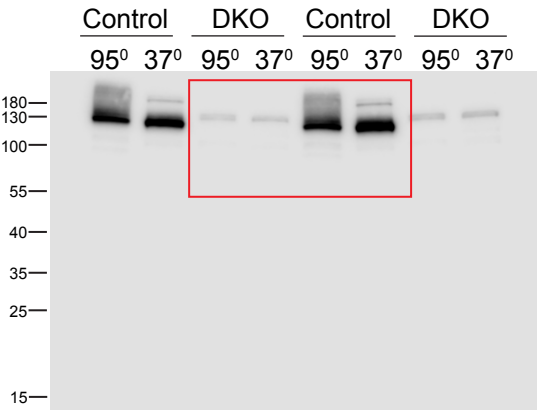

**Cultures #3 and #4**

Syntaxin 1

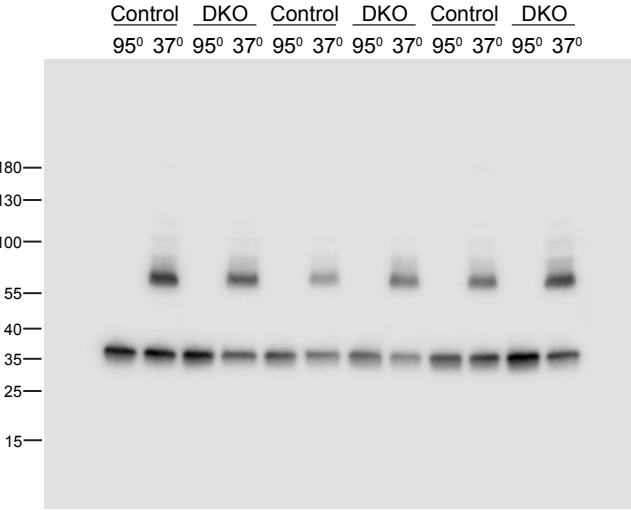

Supplement: Figure 1—source data 1. [file elife-85561-fig1-data1.zip › Figure 1_source files/Figure 1_uncropped blots.pdf]

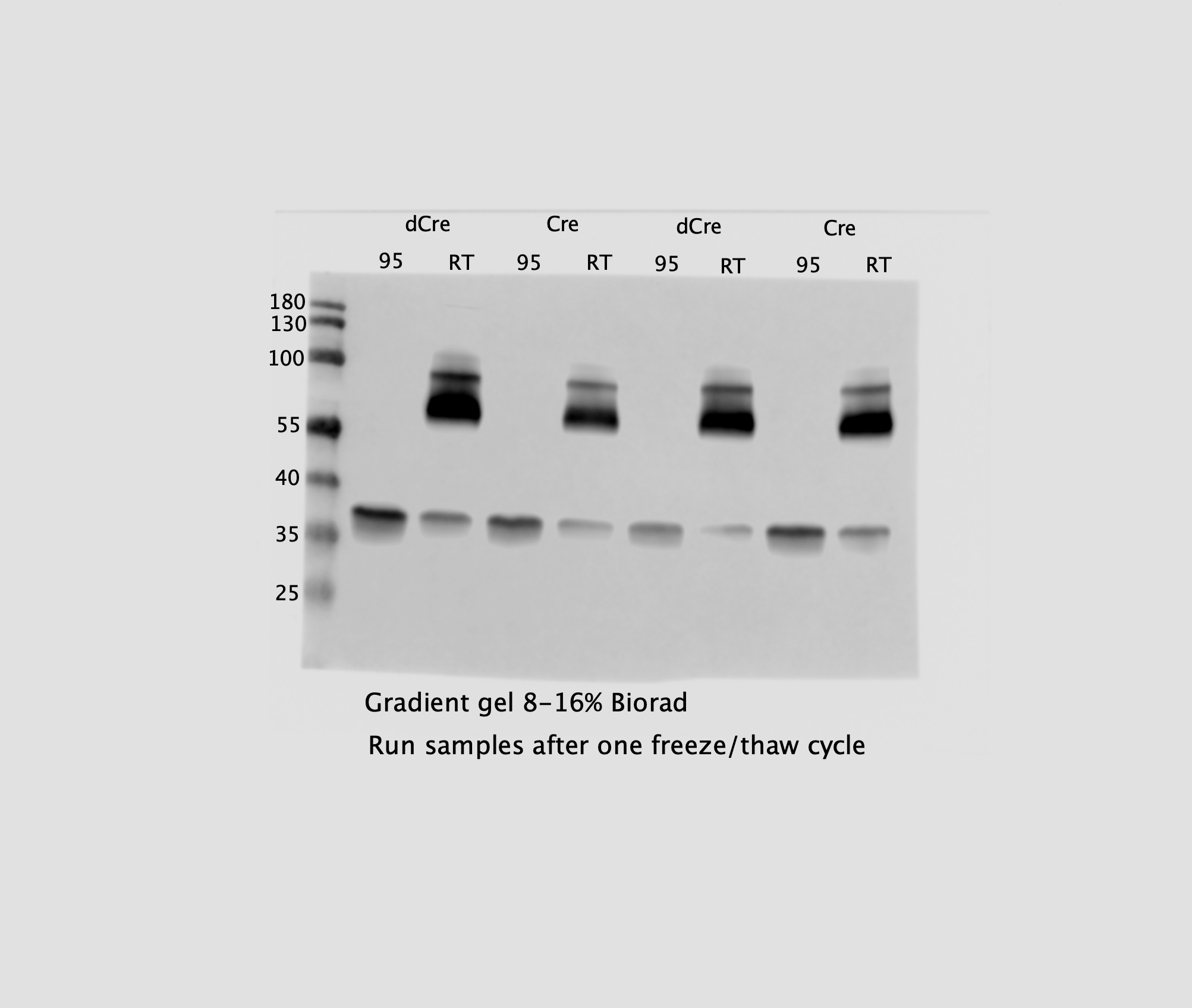

Supplement: Figure 1—source data 1. [file elife-85561-fig1-data1.zip › Figure 1_source files/Syntaxin_replicate1_ladder.tif]

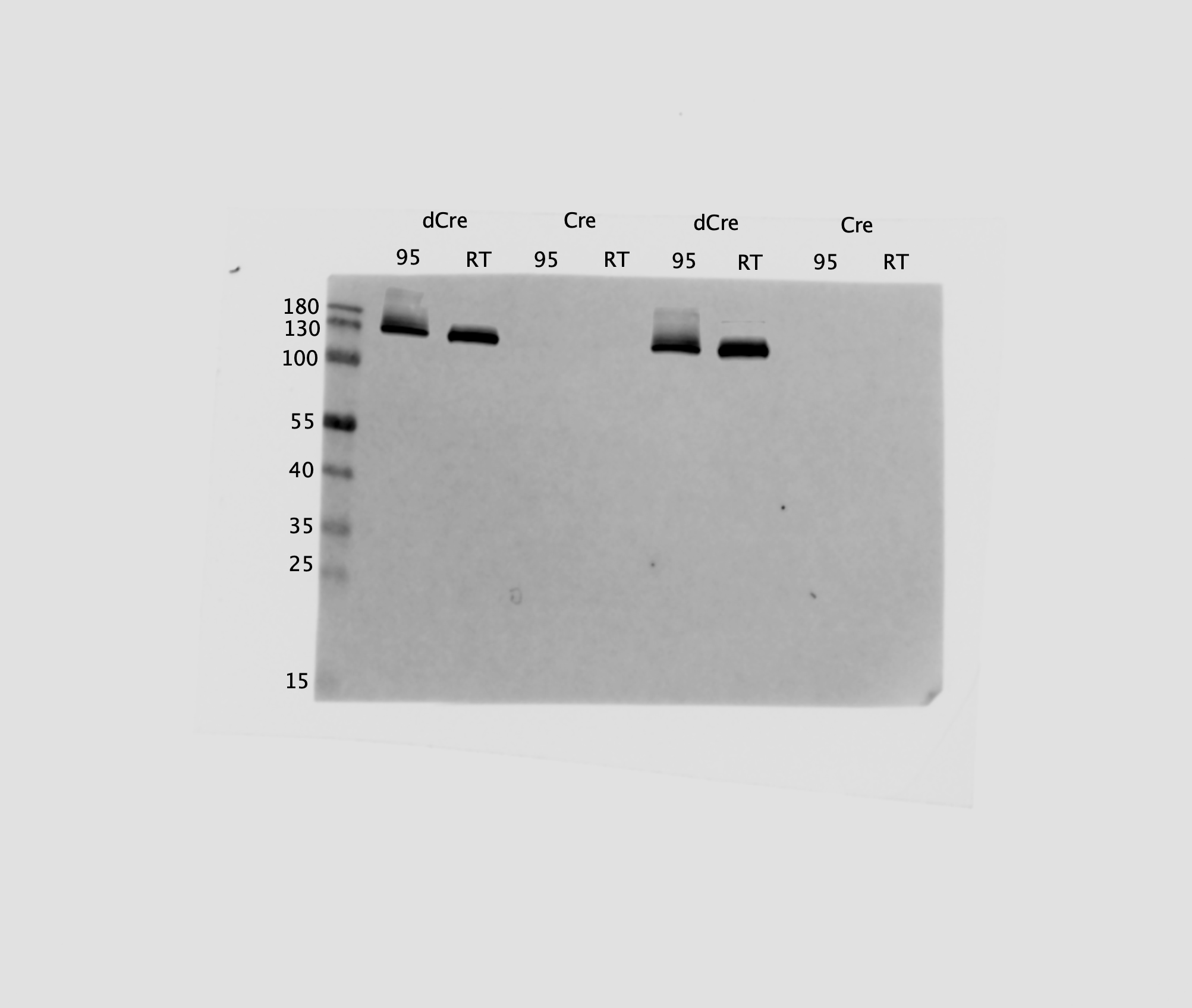

Supplement: Figure 1—source data 1. [file elife-85561-fig1-data1.zip › Figure 1_source files/tomosyn_replicate2_ladder.tif]

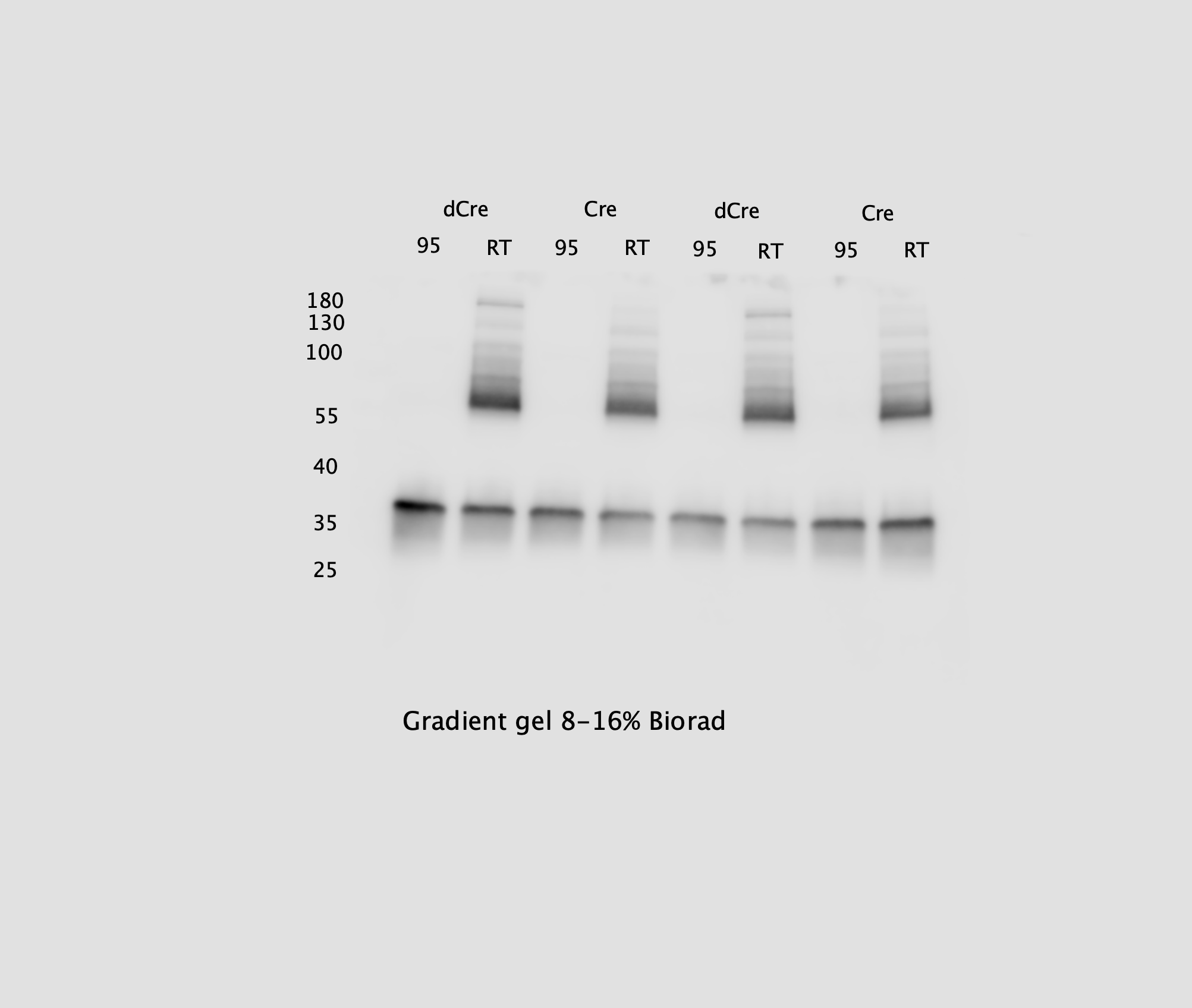

Supplement: Figure 1—source data 1. [file elife-85561-fig1-data1.zip › Figure 1_source files/Syntaxin_replicate2.tif]

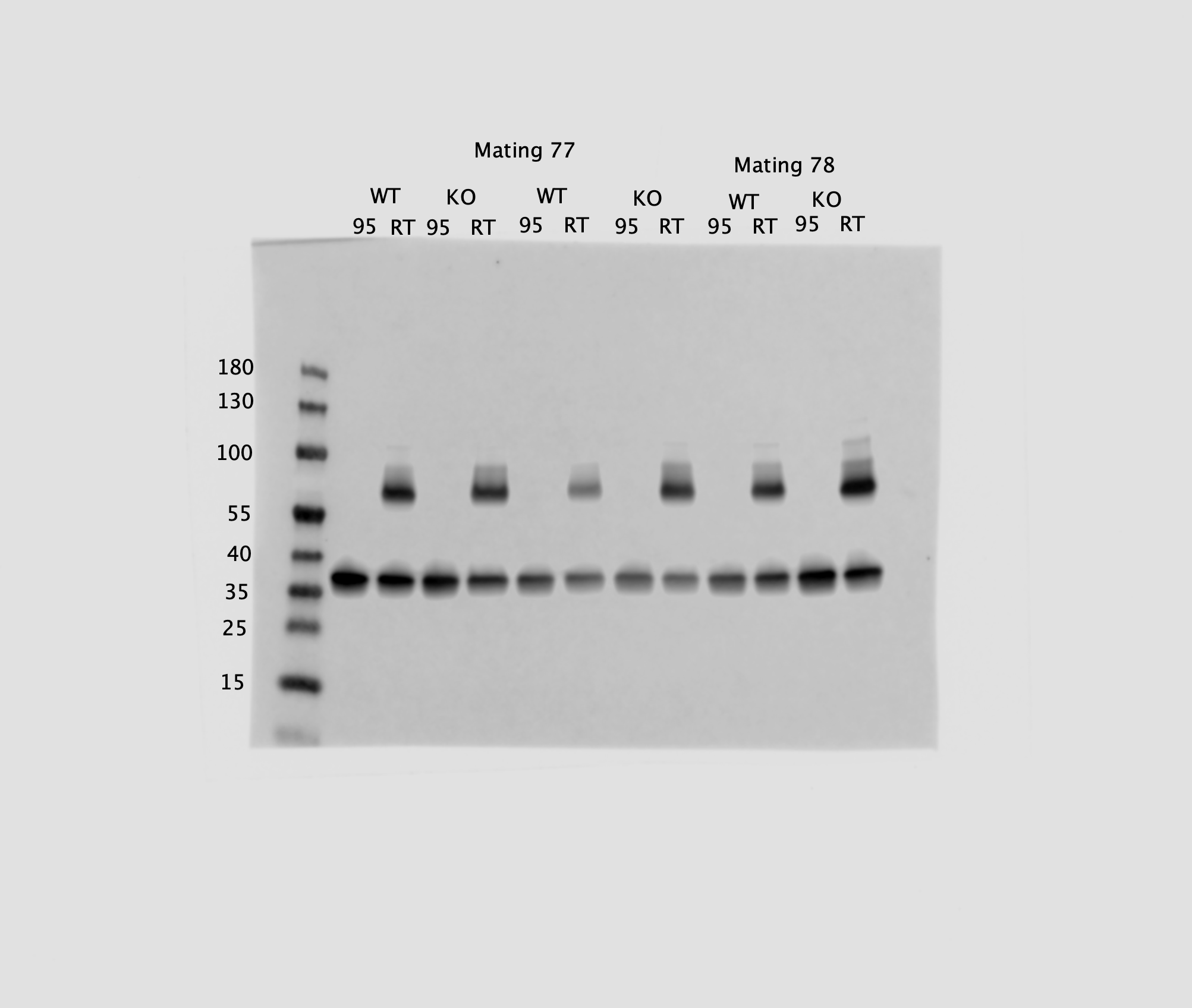

Supplement: Figure 1—source data 1. [file elife-85561-fig1-data1.zip › Figure 1_source files/Syntaxin_replicates3-4_ladder.tif]

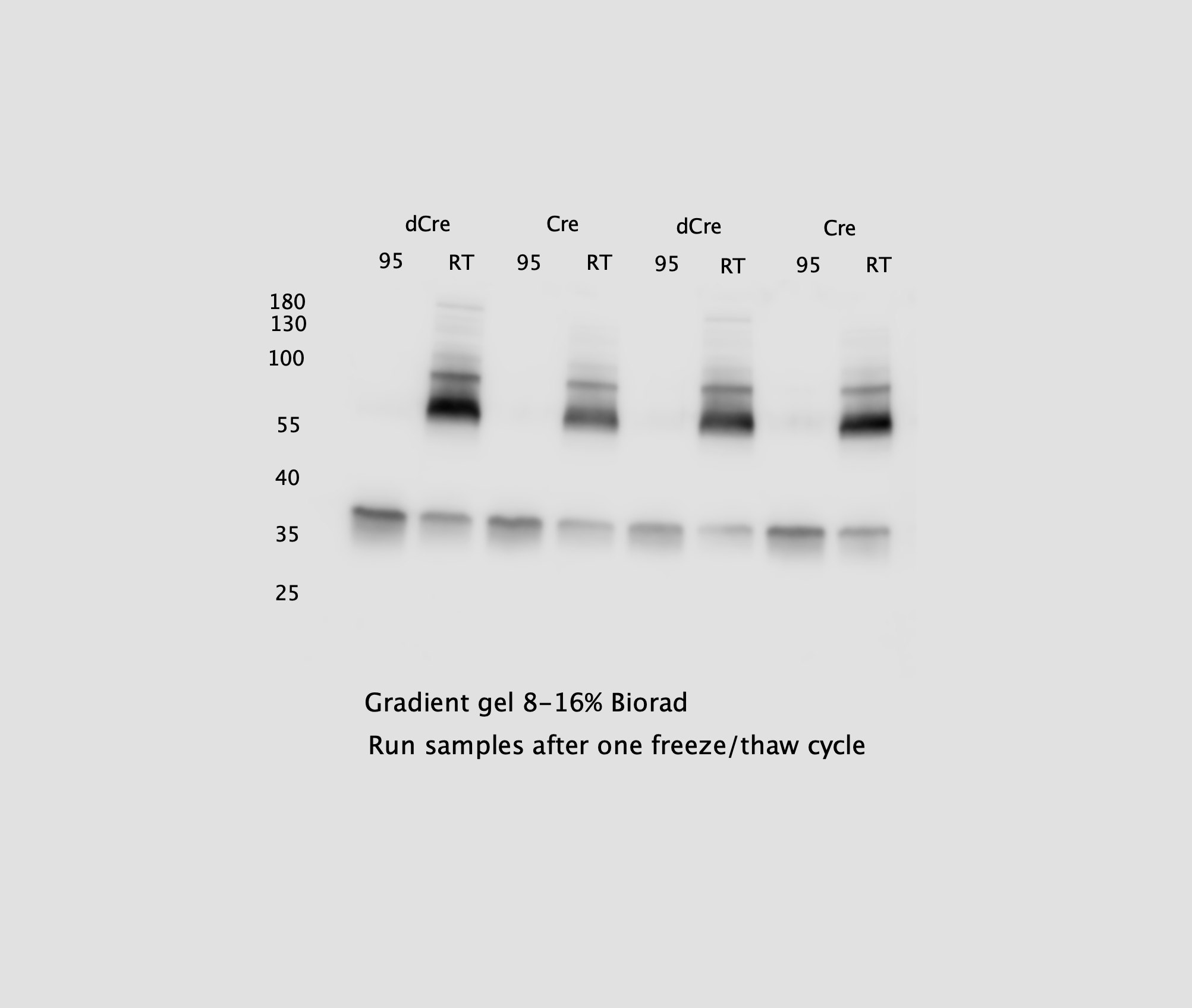

Supplement: Figure 1—source data 1. [file elife-85561-fig1-data1.zip › Figure 1_source files/Syntaxin_replicate1.tif]

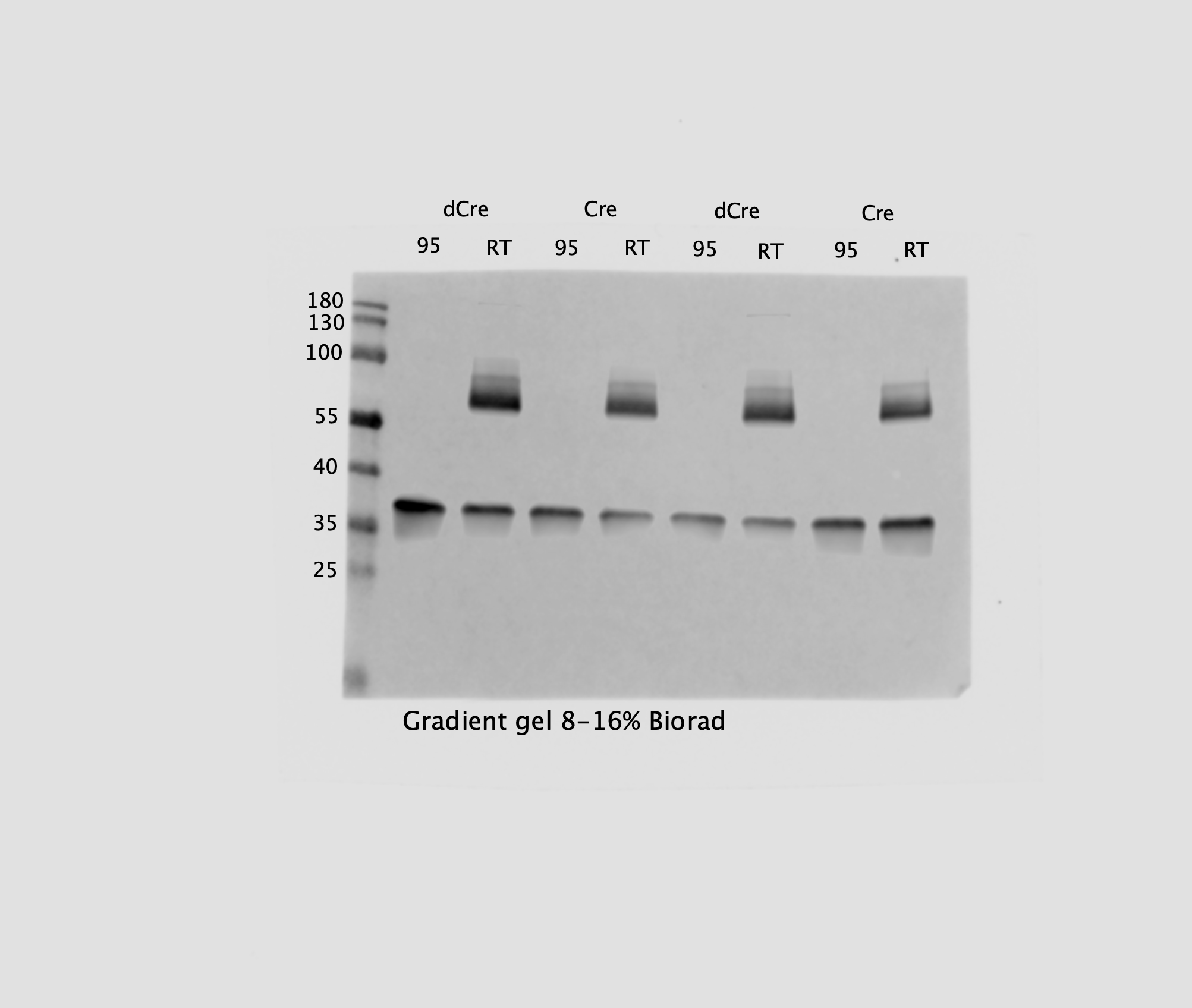

Supplement: Figure 1—source data 1. [file elife-85561-fig1-data1.zip › Figure 1_source files/Syntaxin_replicate2_ladder.tif]

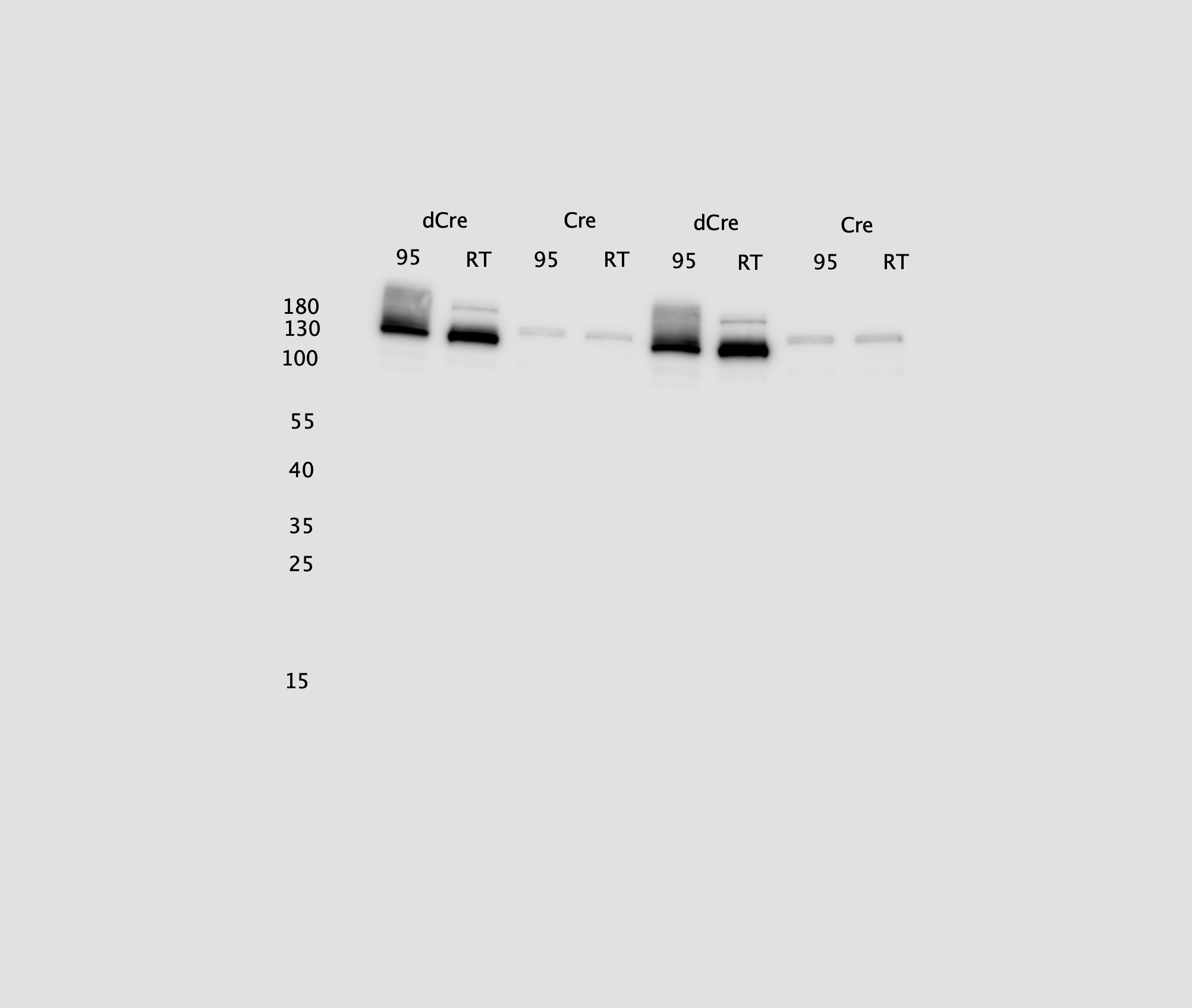

Supplement: Figure 1—source data 1. [file elife-85561-fig1-data1.zip › Figure 1_source files/tomosyn_replicate2.tif]

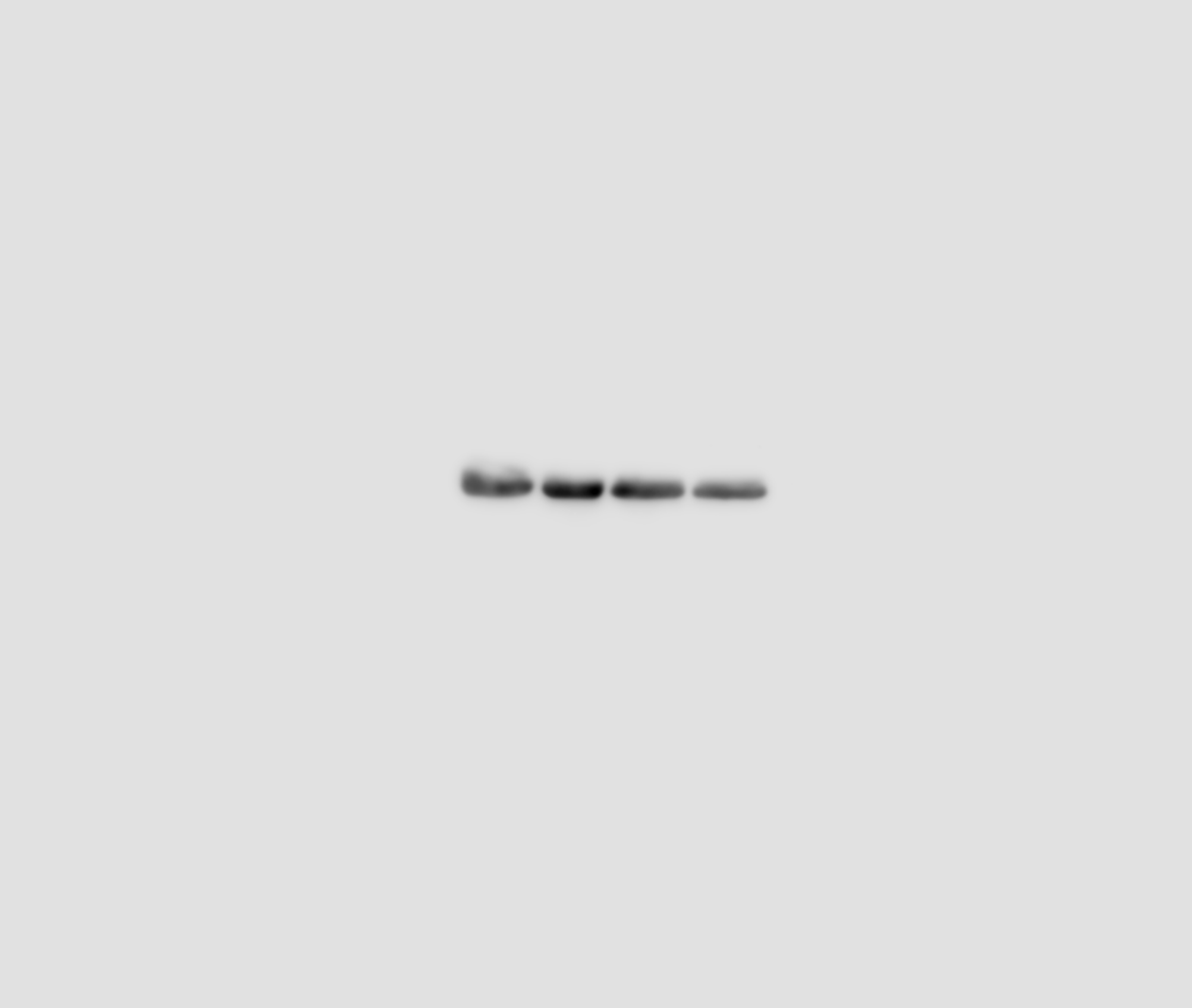

Supplement: Figure 1—figure supplement 1—source data 1. [file elife-85561-fig1-figsupp1-data1.zip › Figure 1 - figure supplement 1_source files/tubulin.tif]

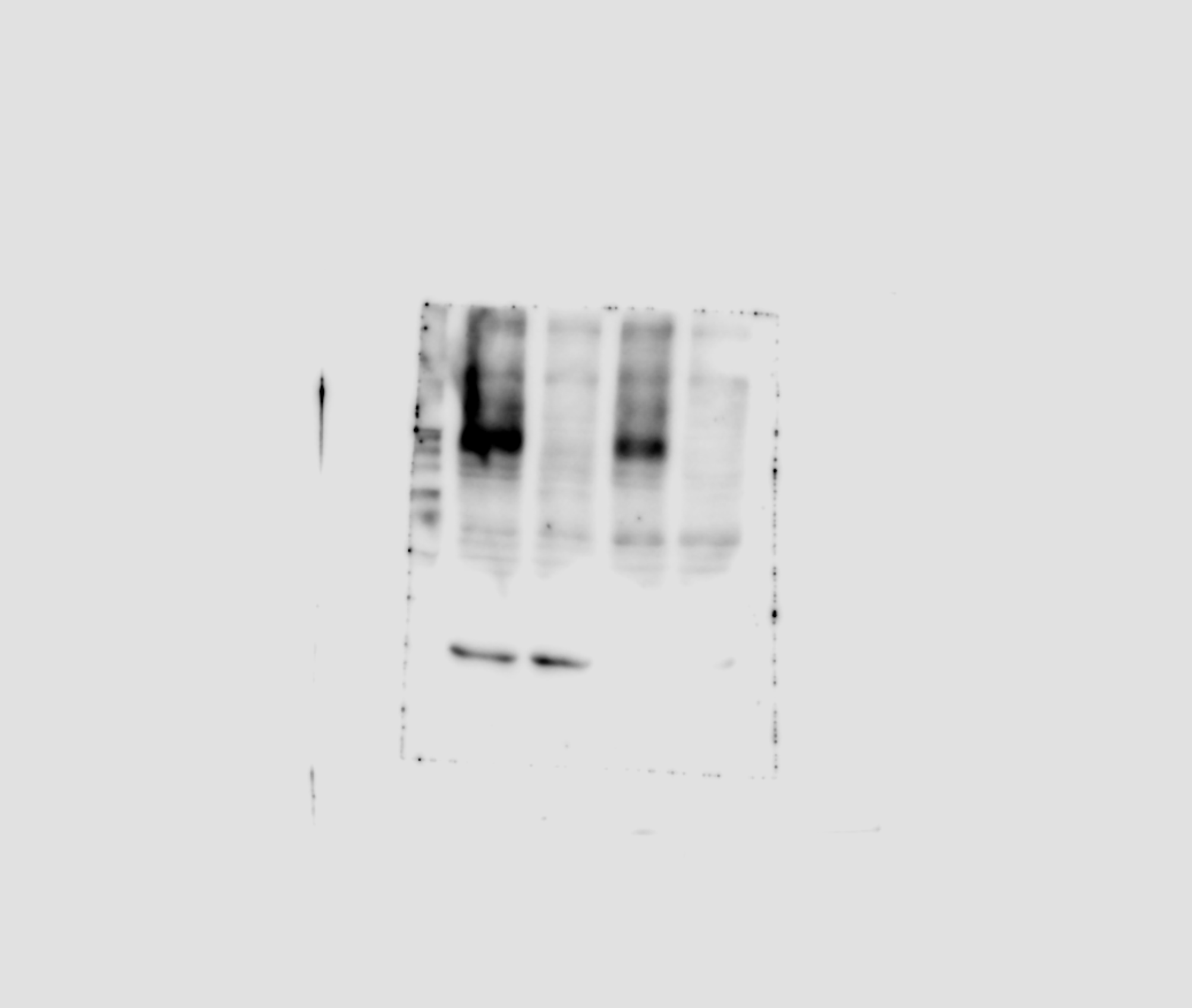

Supplement: Figure 1—figure supplement 1—source data 1. [file elife-85561-fig1-figsupp1-data1.zip › Figure 1 - figure supplement 1_source files/tomosyn-2.tif]

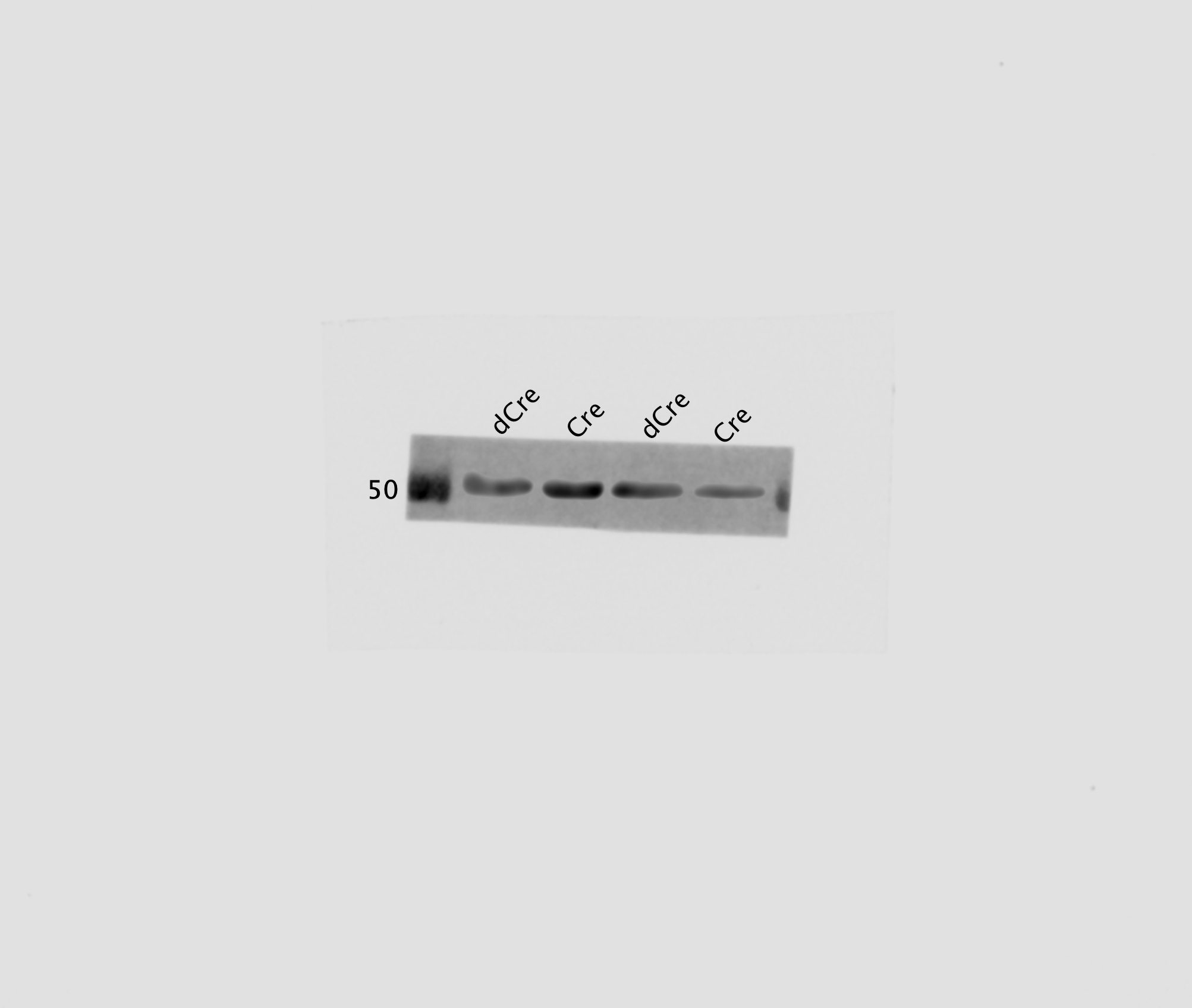

Supplement: Figure 1—figure supplement 1—source data 1. [file elife-85561-fig1-figsupp1-data1.zip › Figure 1 - figure supplement 1_source files/tubulin_ladder.tif]

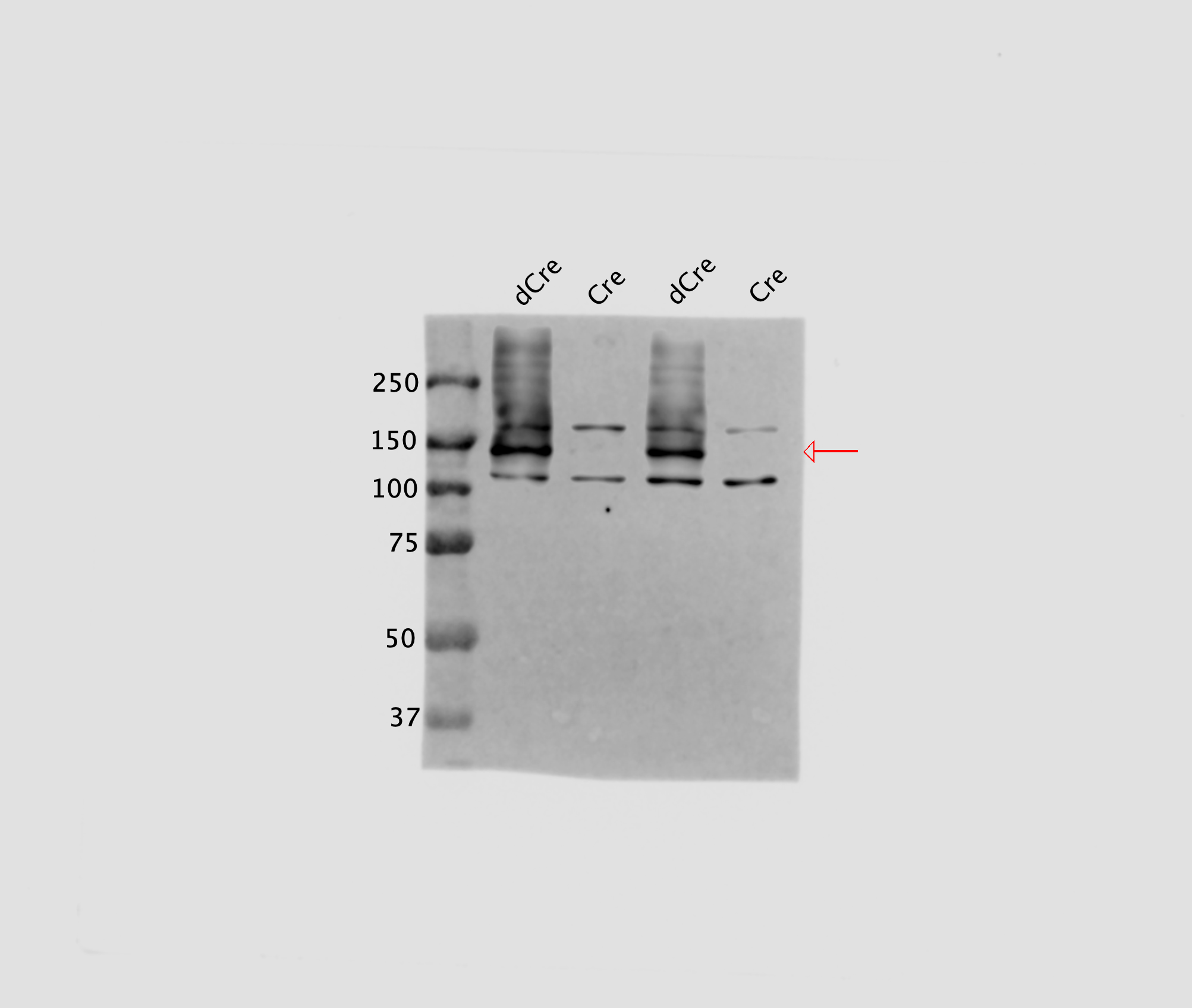

Supplement: Figure 1—figure supplement 1—source data 1. [file elife-85561-fig1-figsupp1-data1.zip › Figure 1 - figure supplement 1_source files/tomosyn_ladder.tif]

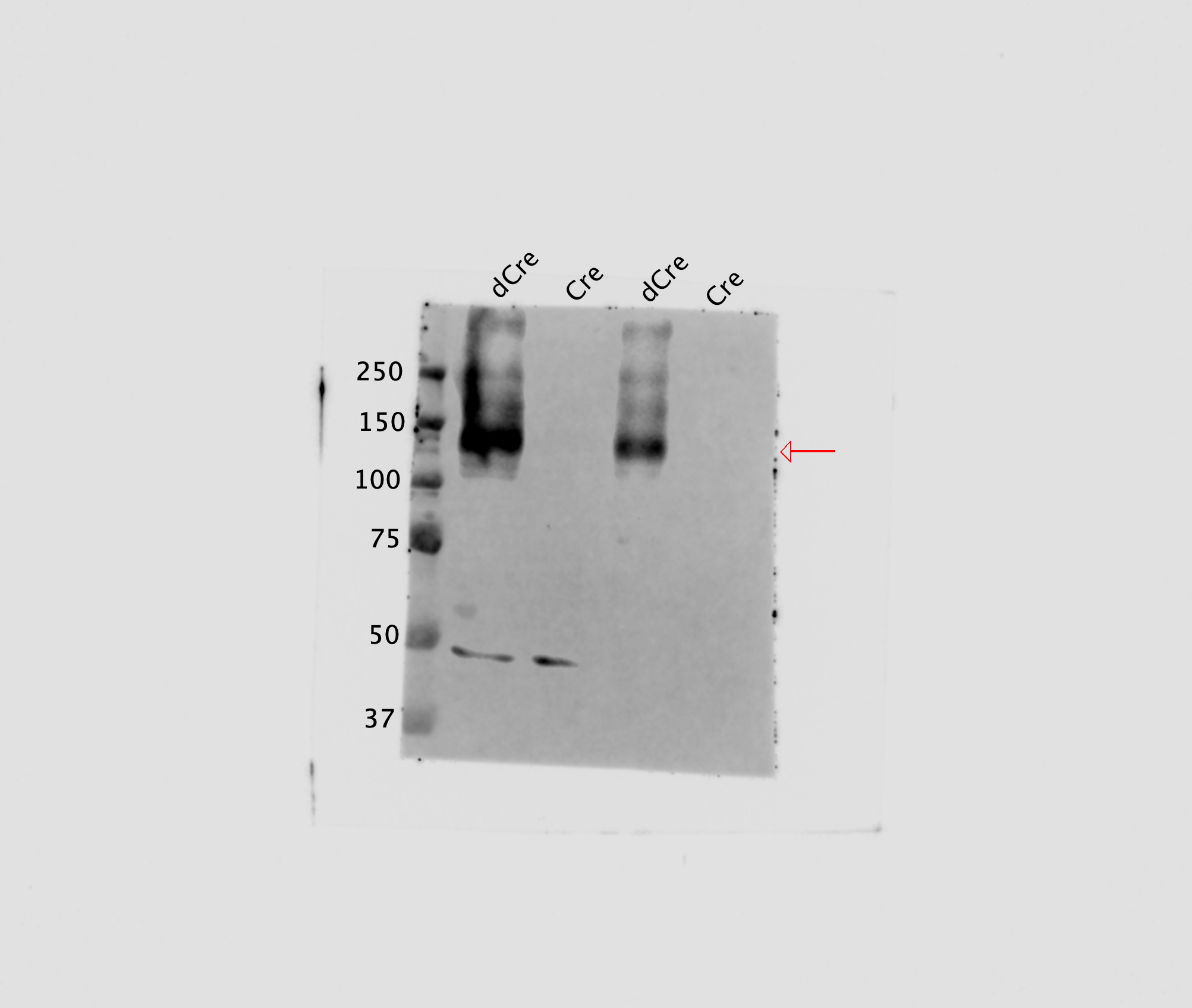

Supplement: Figure 1—figure supplement 1—source data 1. [file elife-85561-fig1-figsupp1-data1.zip › Figure 1 - figure supplement 1_source files/tomosyn-2_ladder.tif]

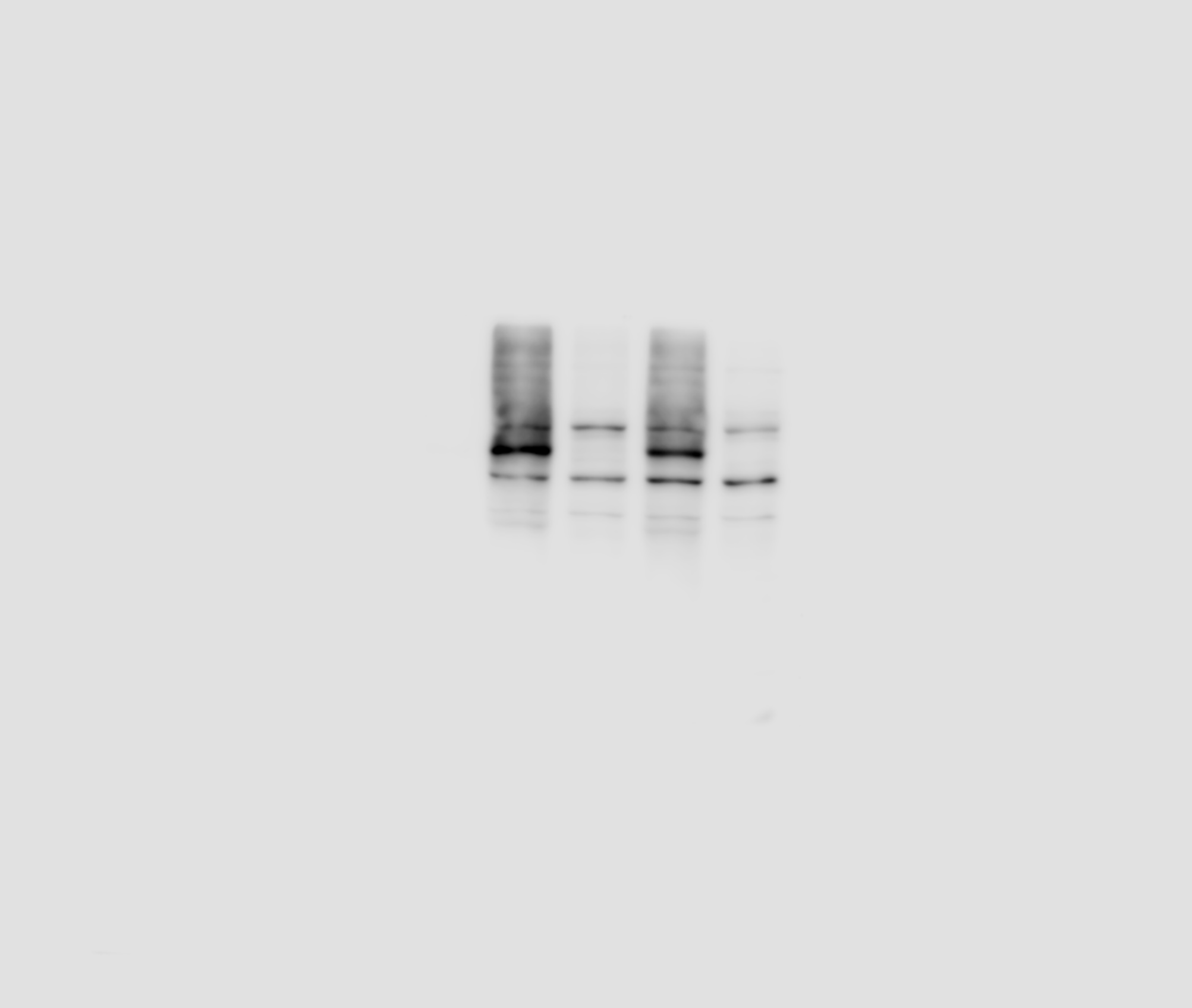

Supplement: Figure 1—figure supplement 1—source data 1. [file elife-85561-fig1-figsupp1-data1.zip › Figure 1 - figure supplement 1_source files/tomosyn.tif]

Tomosyn (STXBP5)

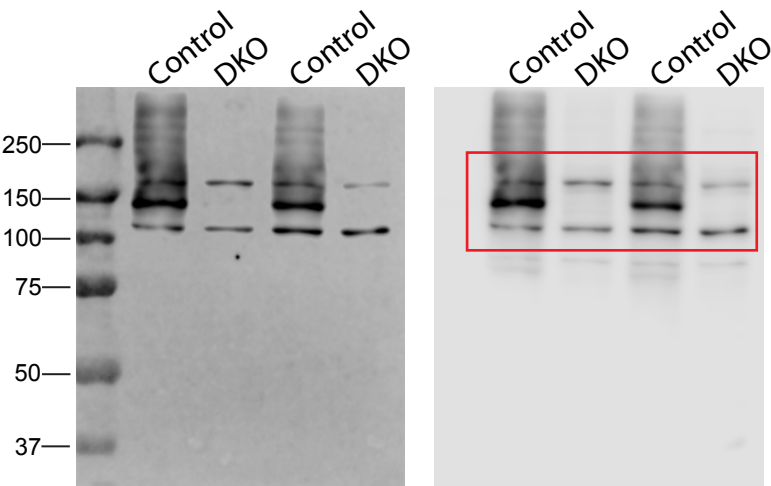

Tomosyn-2 (STXBP5L)

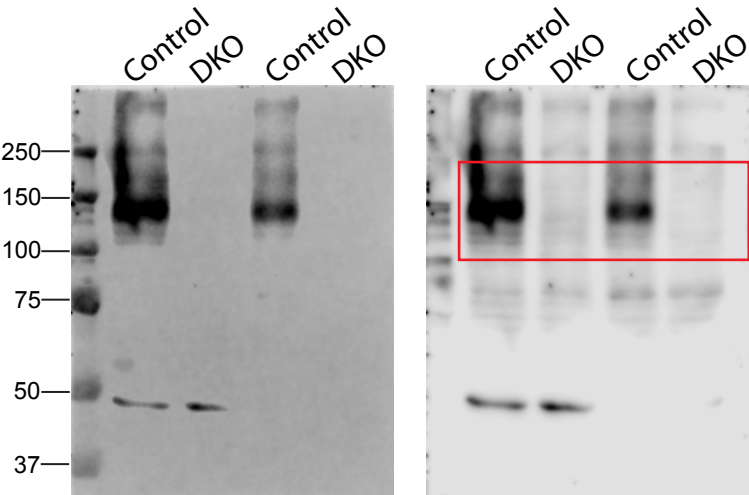

Tubulin (membrane was cut to detect other proteins)

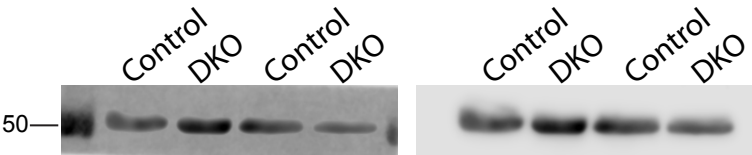

Supplement: Figure 1—figure supplement 1—source data 1. [file elife-85561-fig1-figsupp1-data1.zip › Figure 1 - figure supplement 1_source files/Figure 1_supplement 1_uncropped blots.pdf]

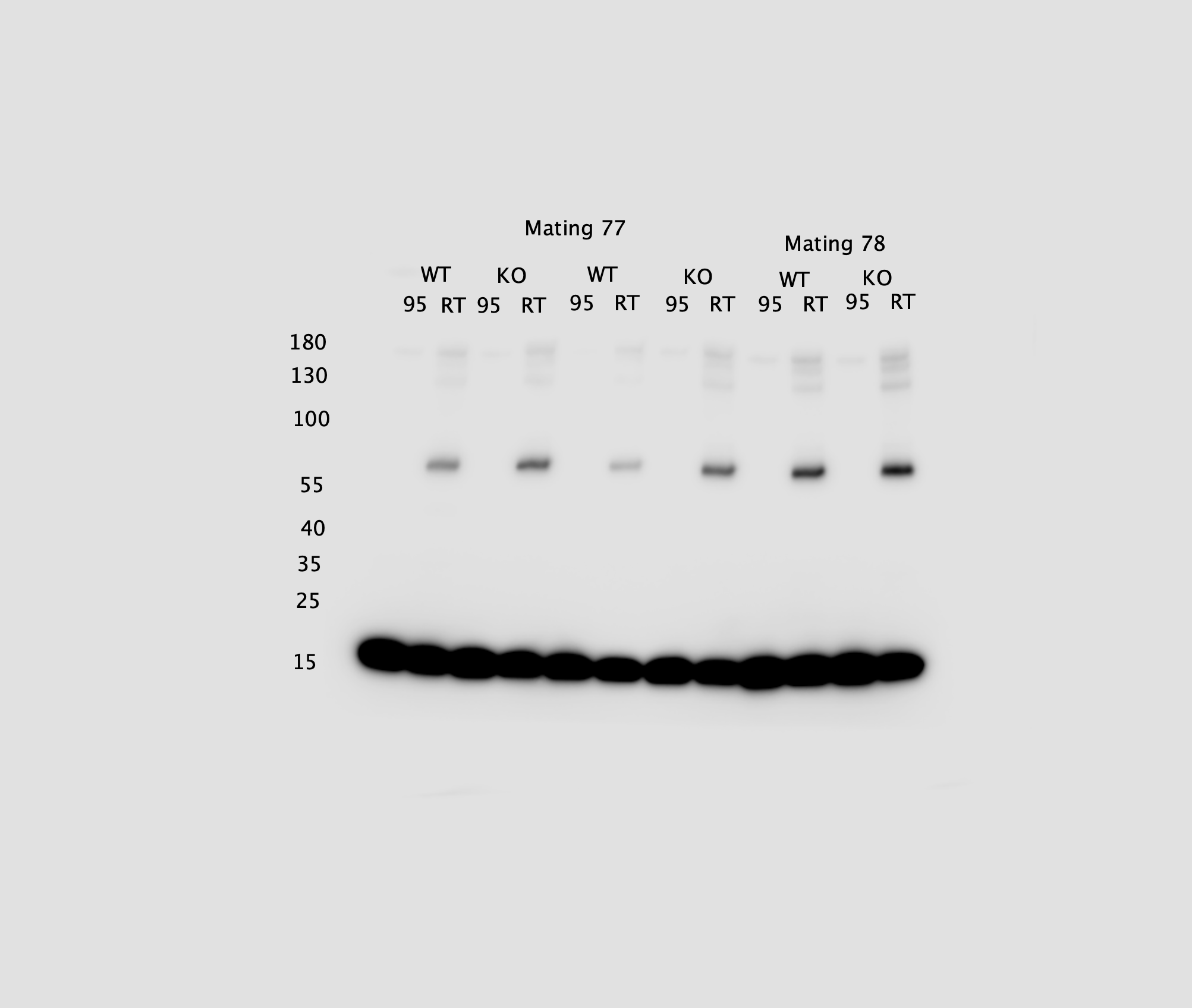

Supplement: Figure 1—figure supplement 3—source data 1. [file elife-85561-fig1-figsupp3-data1.zip › Figure 1 - figure supplement 3_source data/VAMP2_replicates2-3.tif]

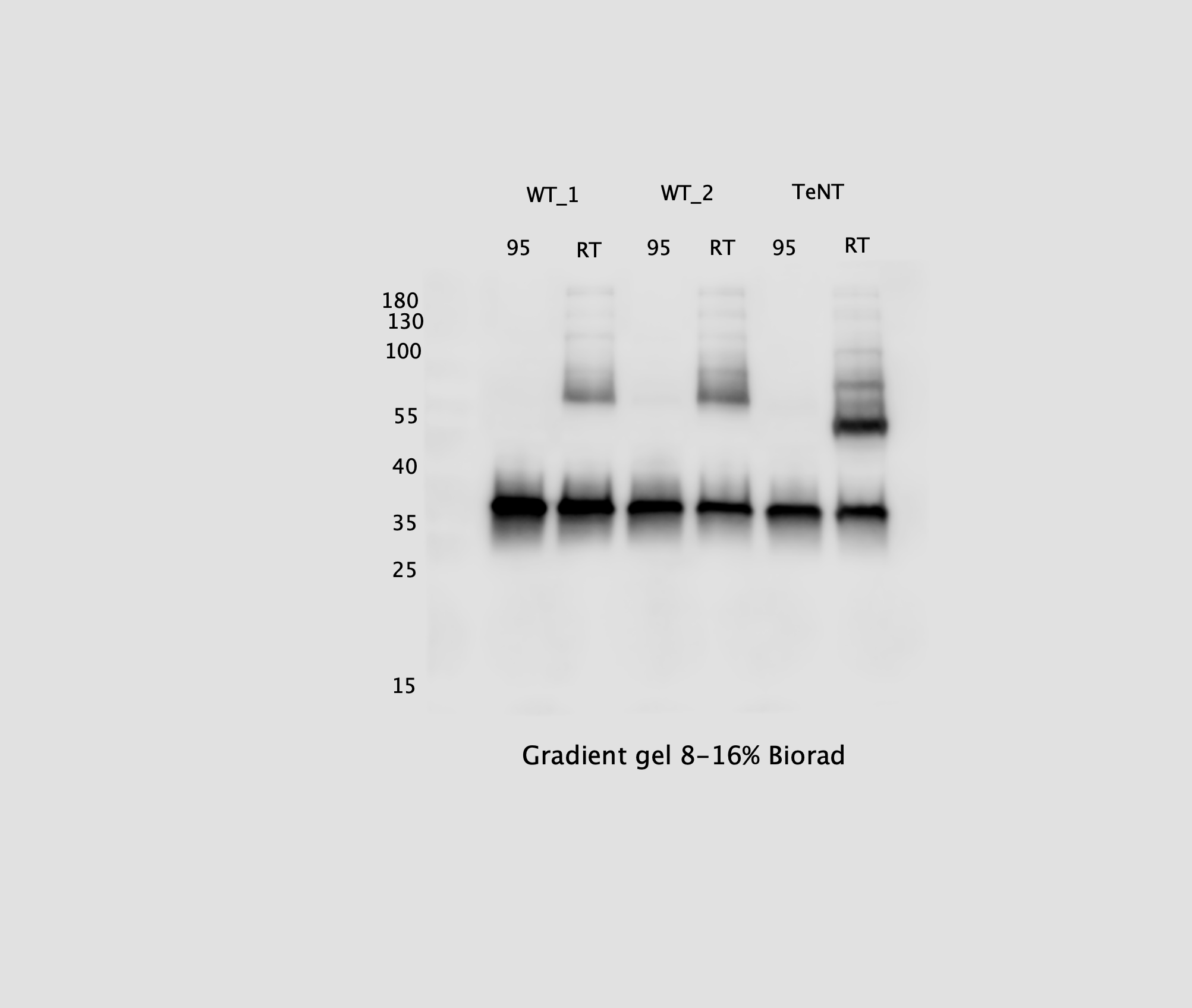

Supplement: Figure 1—figure supplement 3—source data 1. [file elife-85561-fig1-figsupp3-data1.zip › Figure 1 - figure supplement 3_source data/Syntaxin-1.tif]

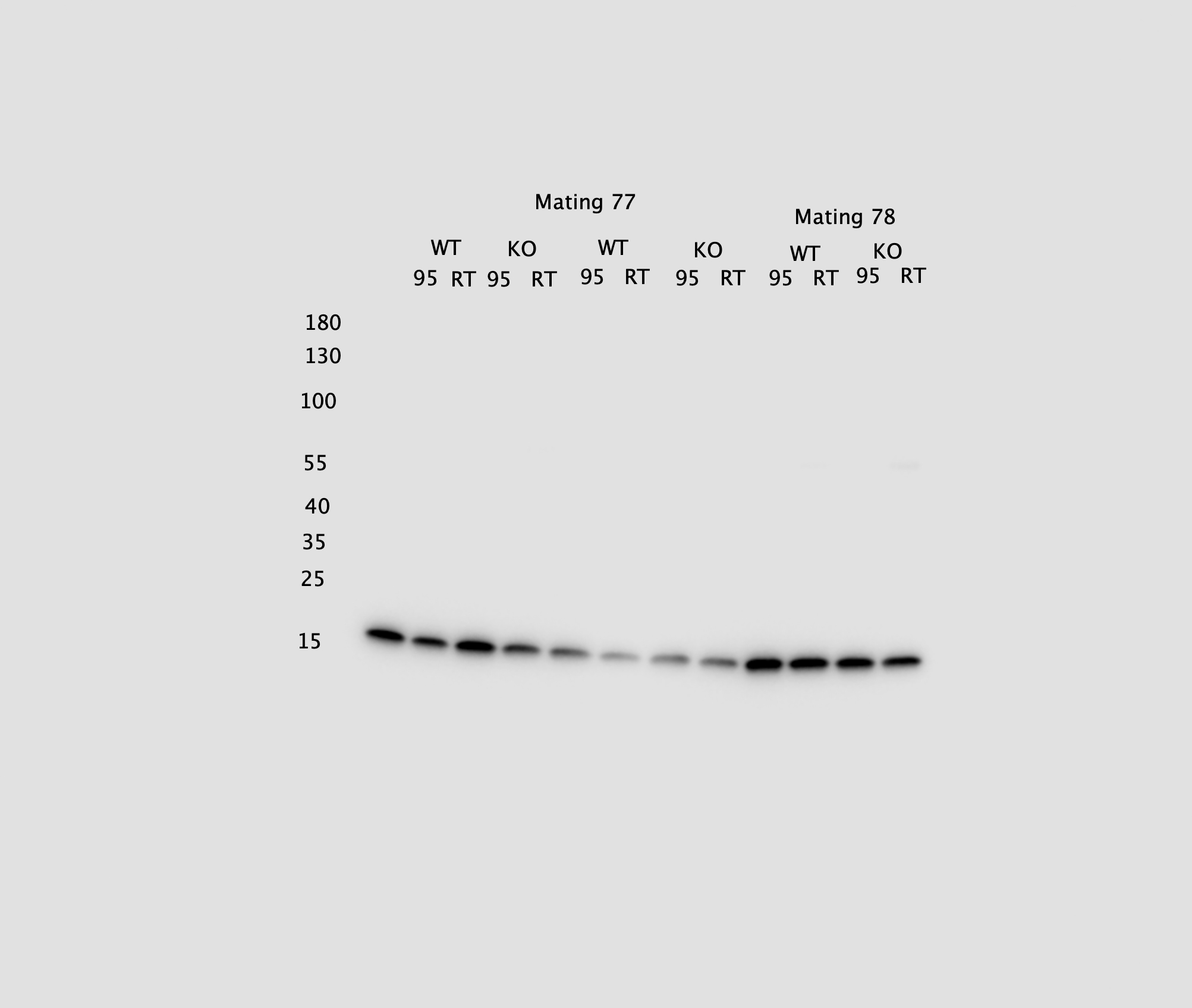

Supplement: Figure 1—figure supplement 3—source data 1. [file elife-85561-fig1-figsupp3-data1.zip › Figure 1 - figure supplement 3_source data/VAMP2_replicates2-3_low exposure.tif]

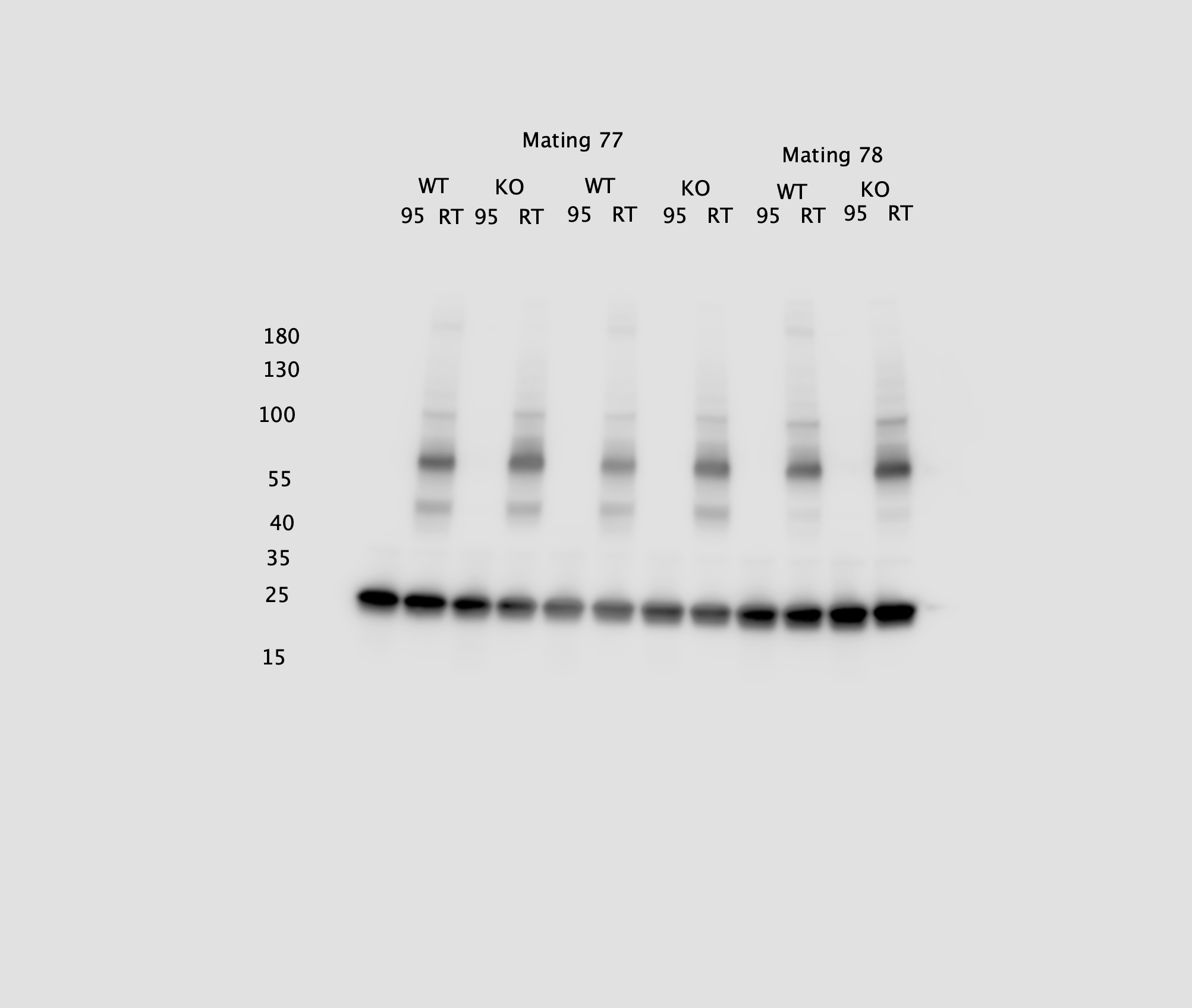

Supplement: Figure 1—figure supplement 3—source data 1. [file elife-85561-fig1-figsupp3-data1.zip › Figure 1 - figure supplement 3_source data/SNAP25_replicates2-3.tif]

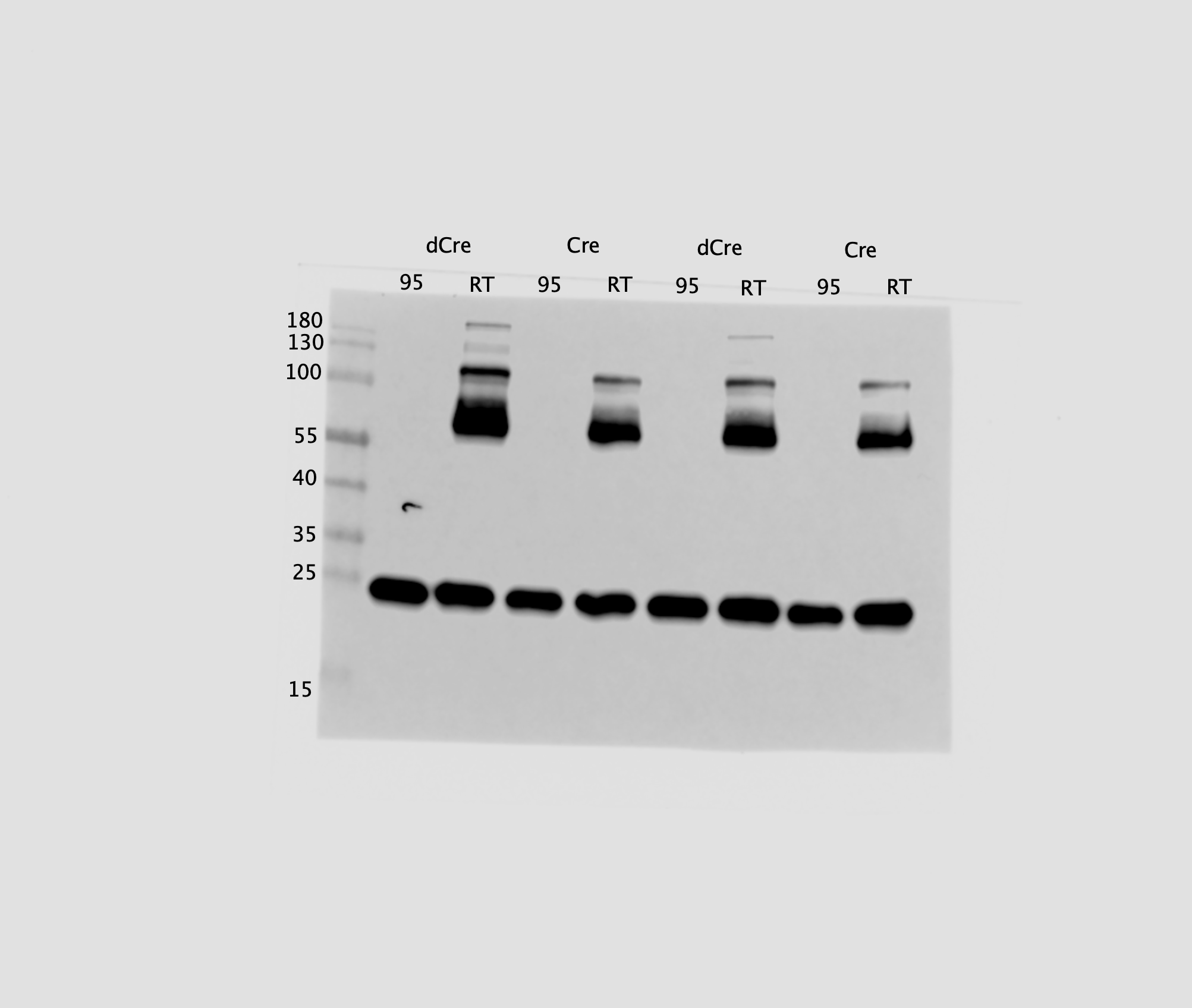

Supplement: Figure 1—figure supplement 3—source data 1. [file elife-85561-fig1-figsupp3-data1.zip › Figure 1 - figure supplement 3_source data/SNAP25_replacate1_ladder.tif]

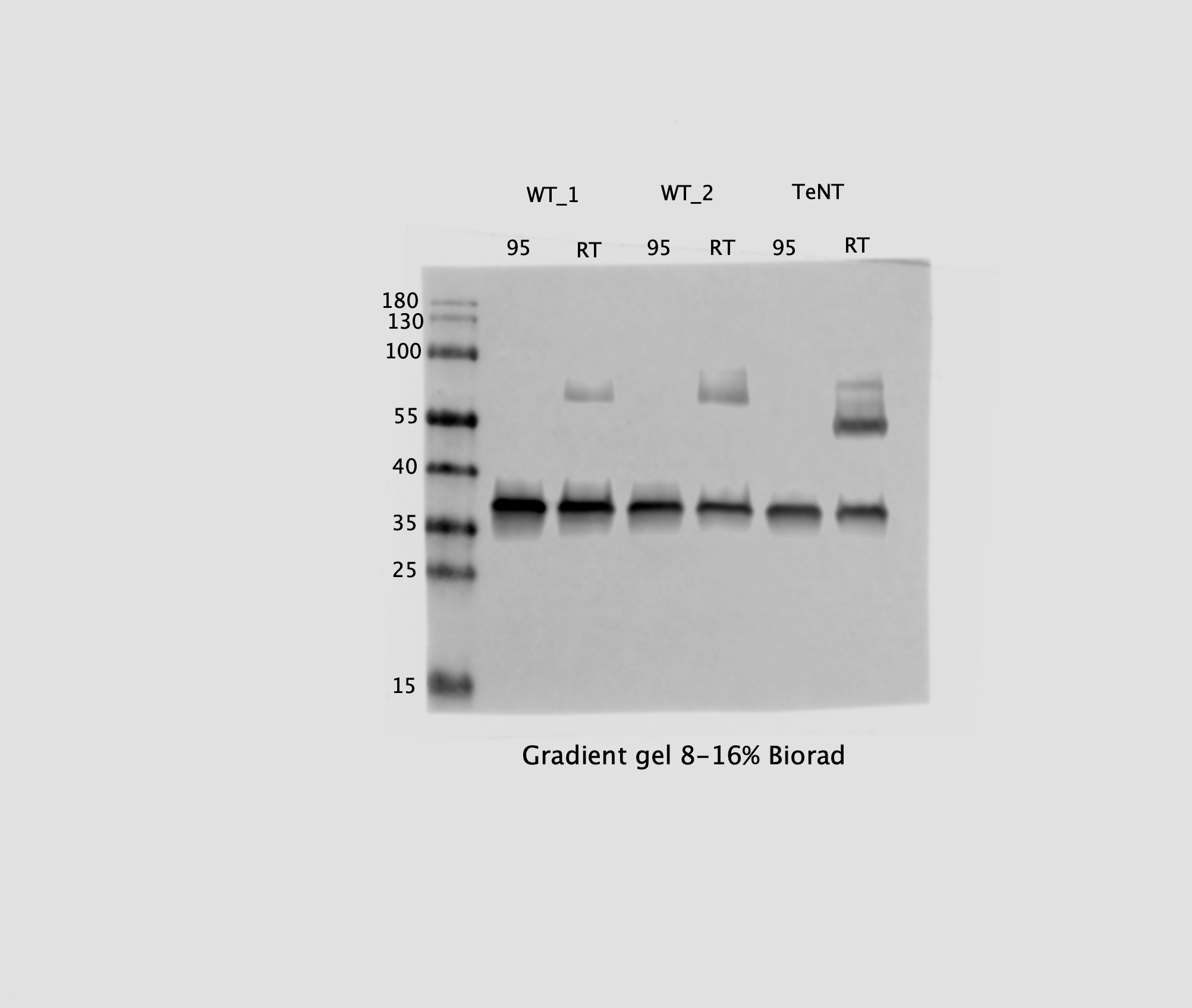

Supplement: Figure 1—figure supplement 3—source data 1. [file elife-85561-fig1-figsupp3-data1.zip › Figure 1 - figure supplement 3_source data/Syntaxin-1_ladder.tif]

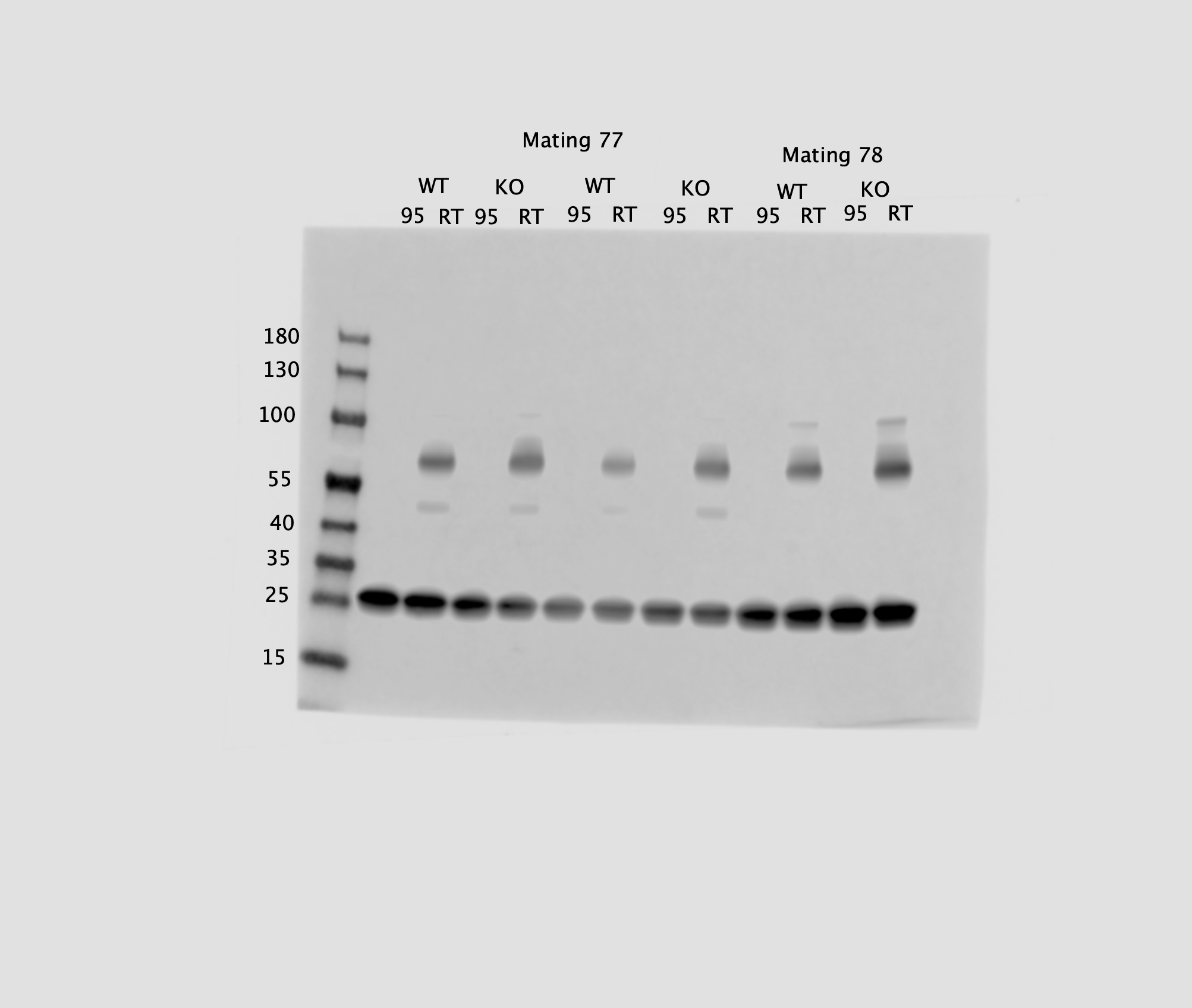

Supplement: Figure 1—figure supplement 3—source data 1. [file elife-85561-fig1-figsupp3-data1.zip › Figure 1 - figure supplement 3_source data/SNAP25_replicates2-3_ladder.tif]

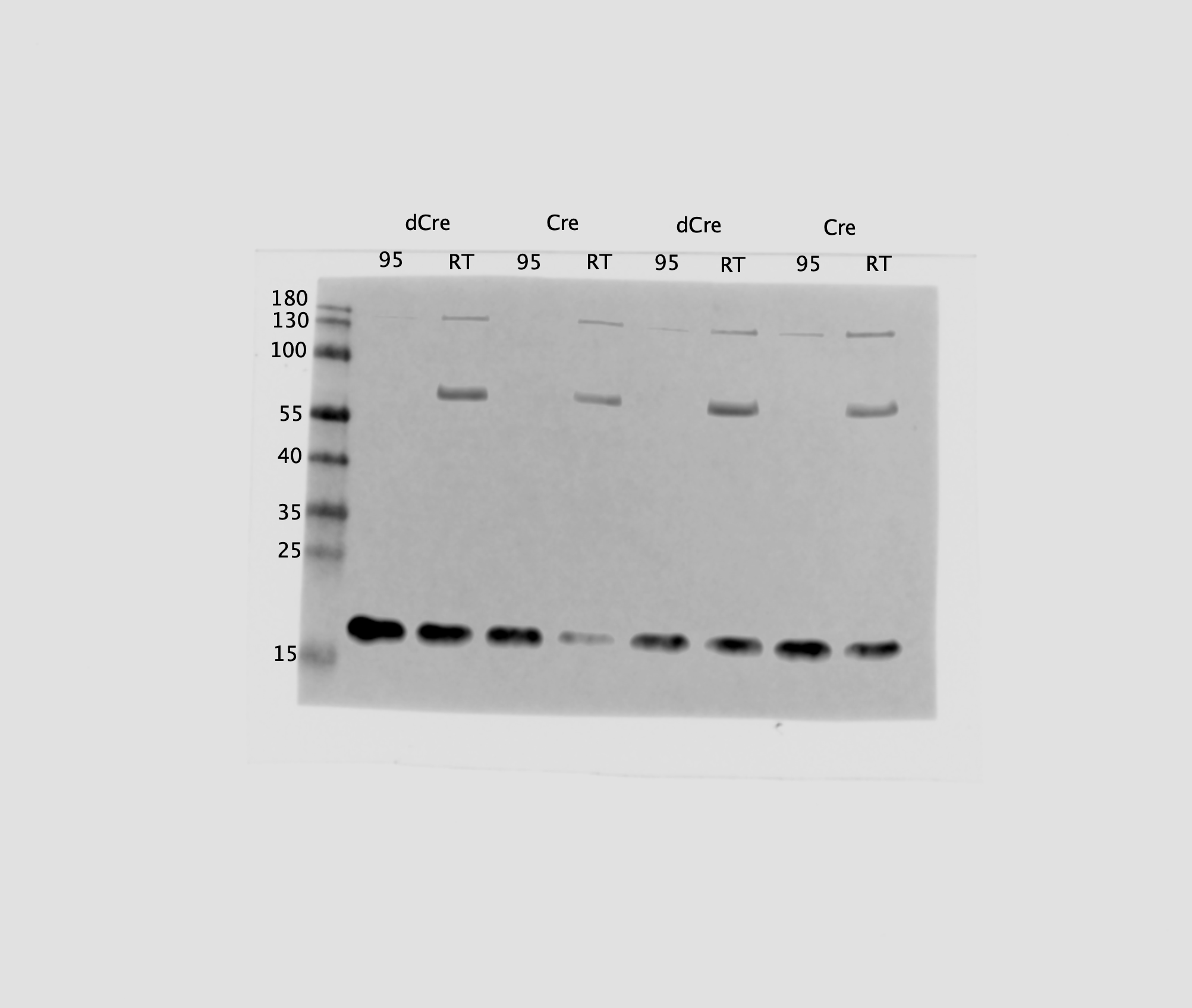

Supplement: Figure 1—figure supplement 3—source data 1. [file elife-85561-fig1-figsupp3-data1.zip › Figure 1 - figure supplement 3_source data/VAMP2_replicate1_ladder.tif]

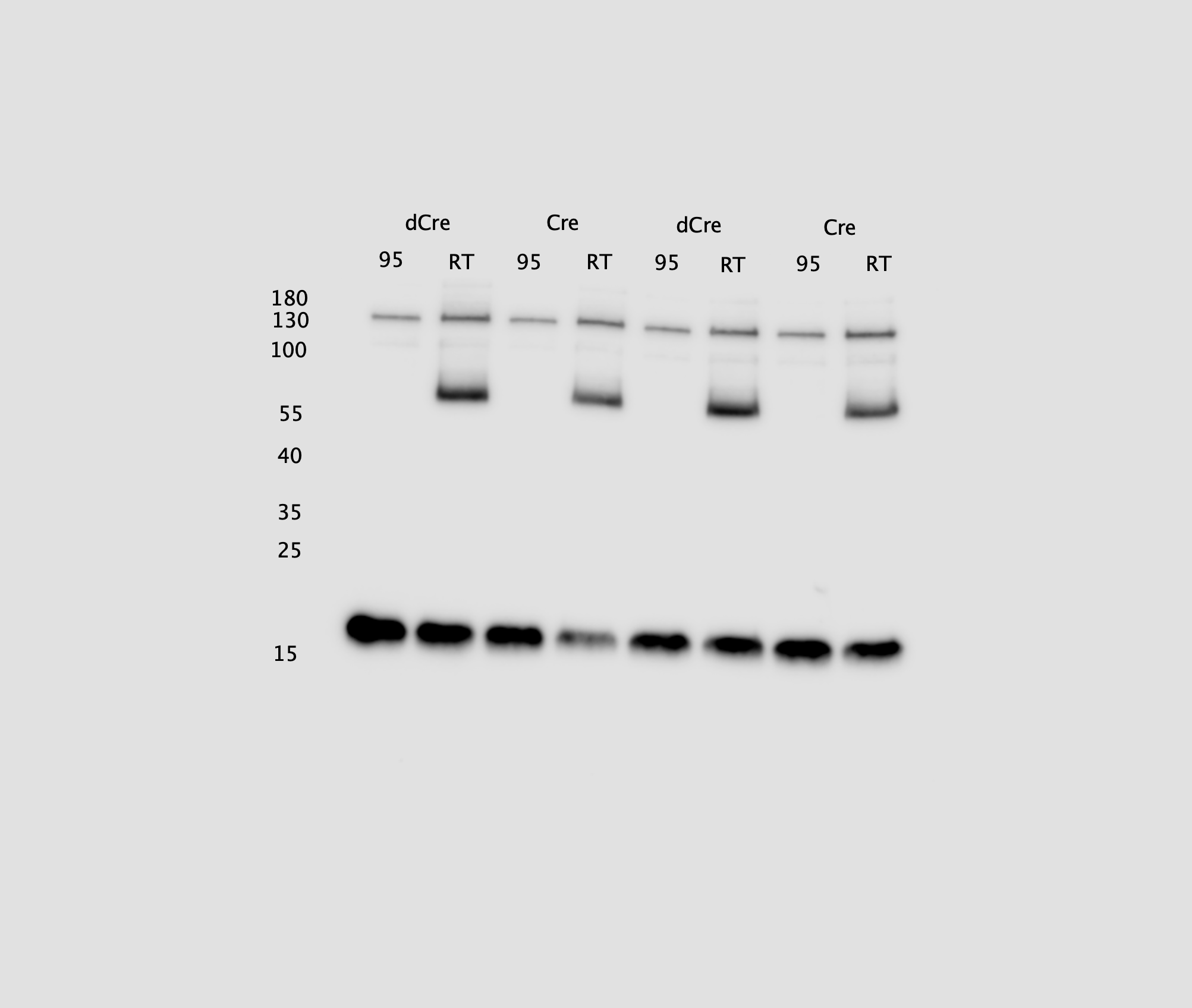

Supplement: Figure 1—figure supplement 3—source data 1. [file elife-85561-fig1-figsupp3-data1.zip › Figure 1 - figure supplement 3_source data/VAMP2_replicate1.tif]

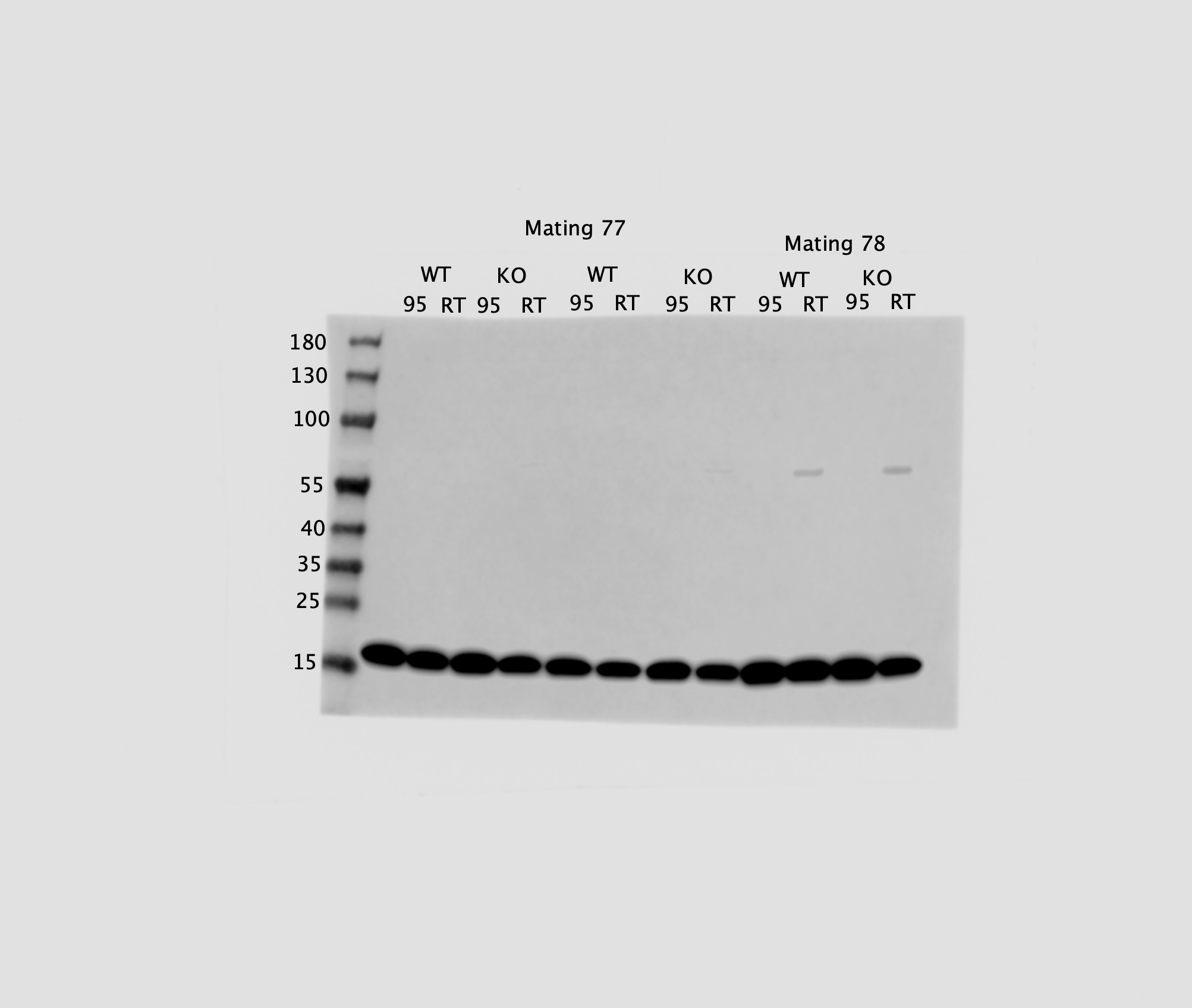

Supplement: Figure 1—figure supplement 3—source data 1. [file elife-85561-fig1-figsupp3-data1.zip › Figure 1 - figure supplement 3_source data/VAMP2_replicates2-3_ladder.tif]

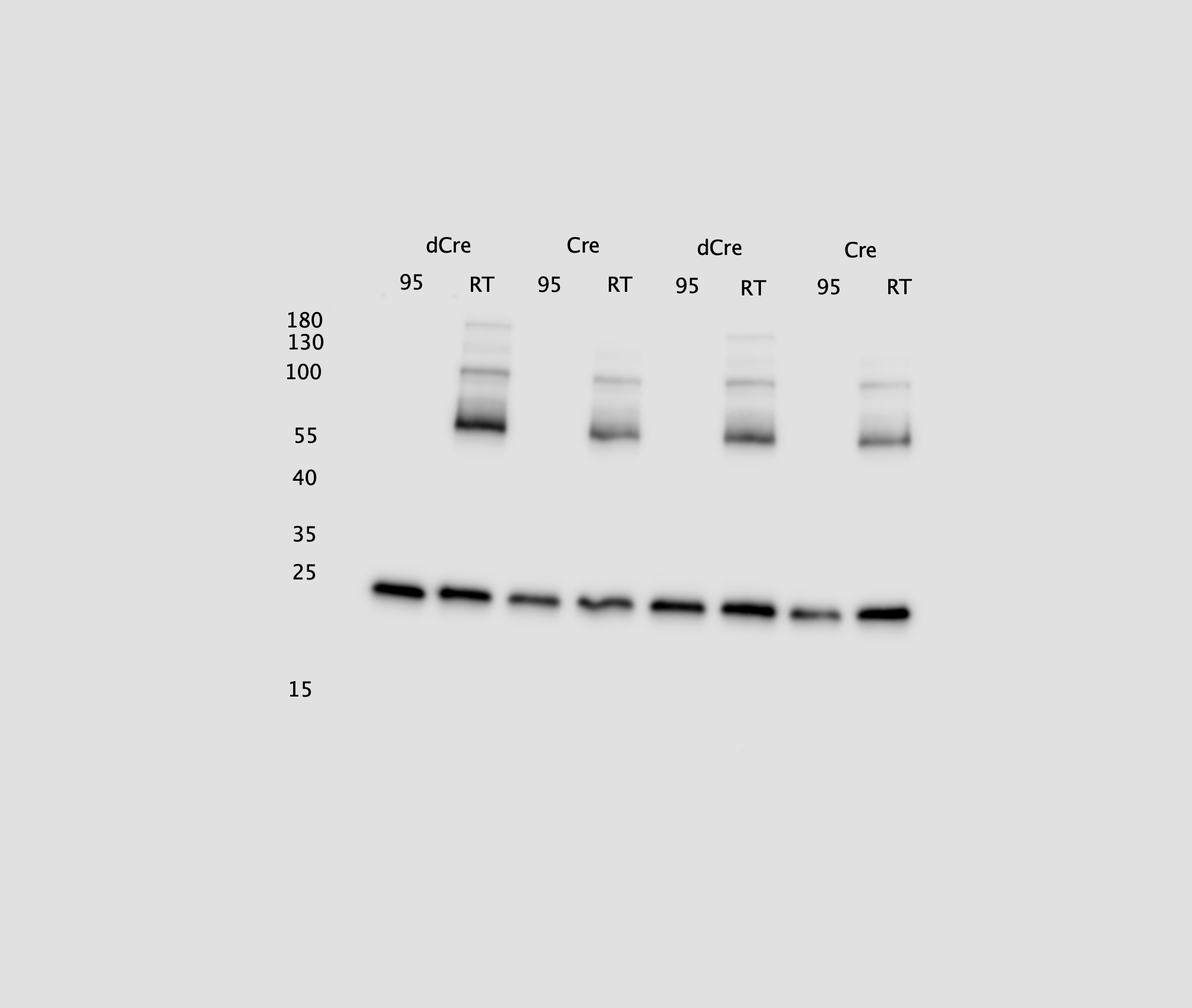

Supplement: Figure 1—figure supplement 3—source data 1. [file elife-85561-fig1-figsupp3-data1.zip › Figure 1 - figure supplement 3_source data/SNAP25_replicate1.tif]

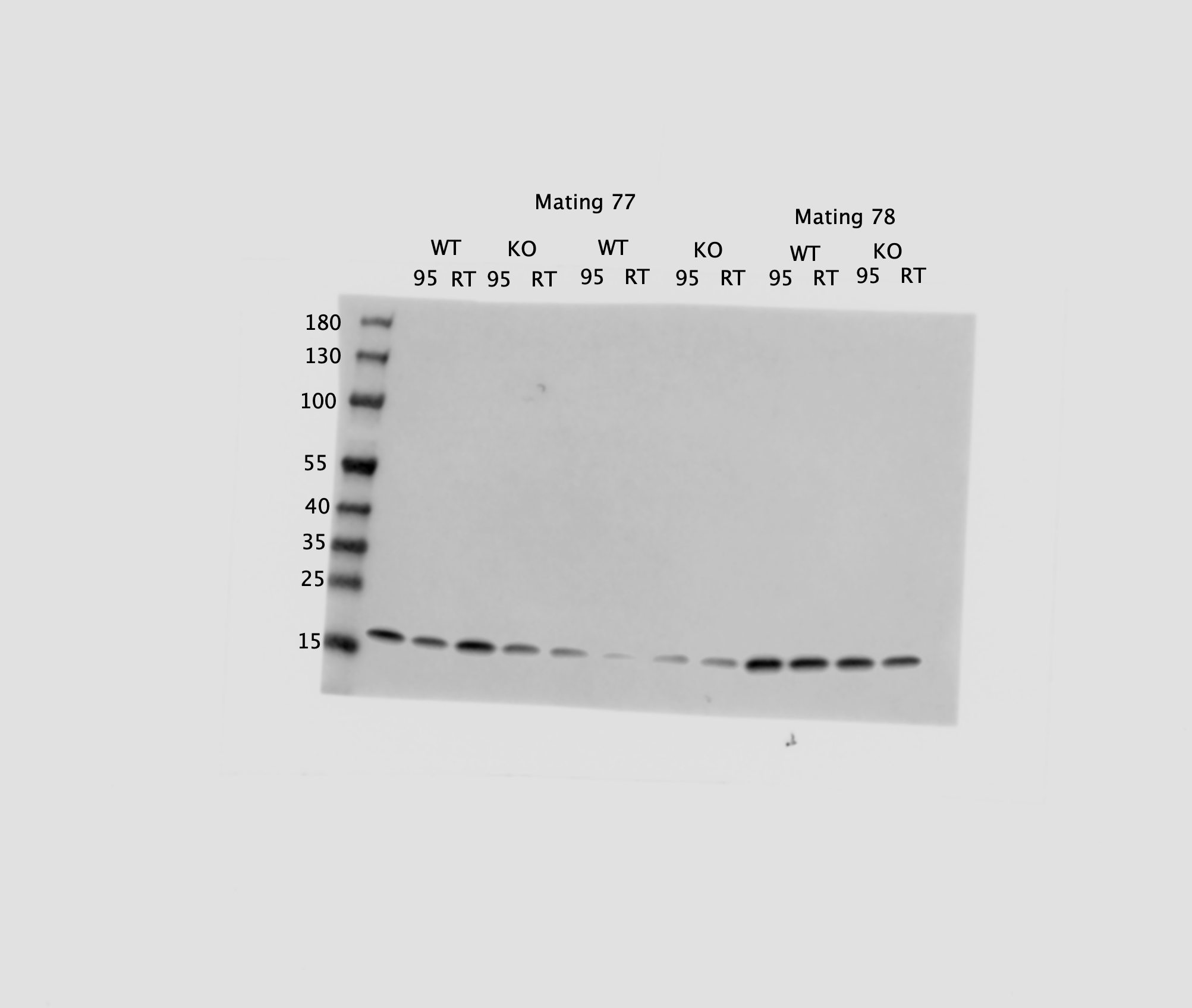

Supplement: Figure 1—figure supplement 3—source data 1. [file elife-85561-fig1-figsupp3-data1.zip › Figure 1 - figure supplement 3_source data/VAMP2_replicates2-3_low exposure_ladder.tif]

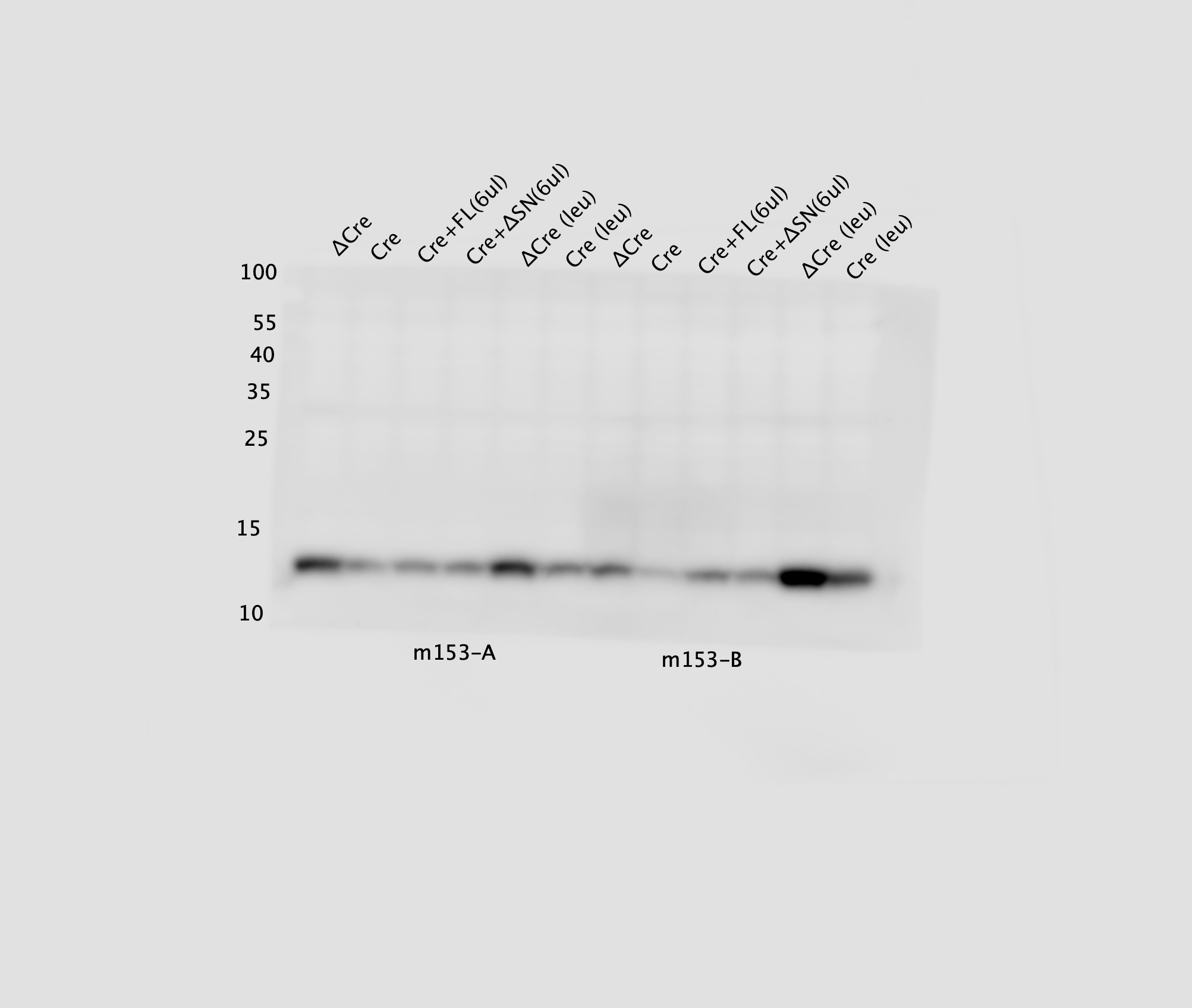

Supplement: Figure 2—source data 1. [file elife-85561-fig2-data1.zip › Figure 2_source files/Figure 2G_source files/BDNF_memb1.tif]

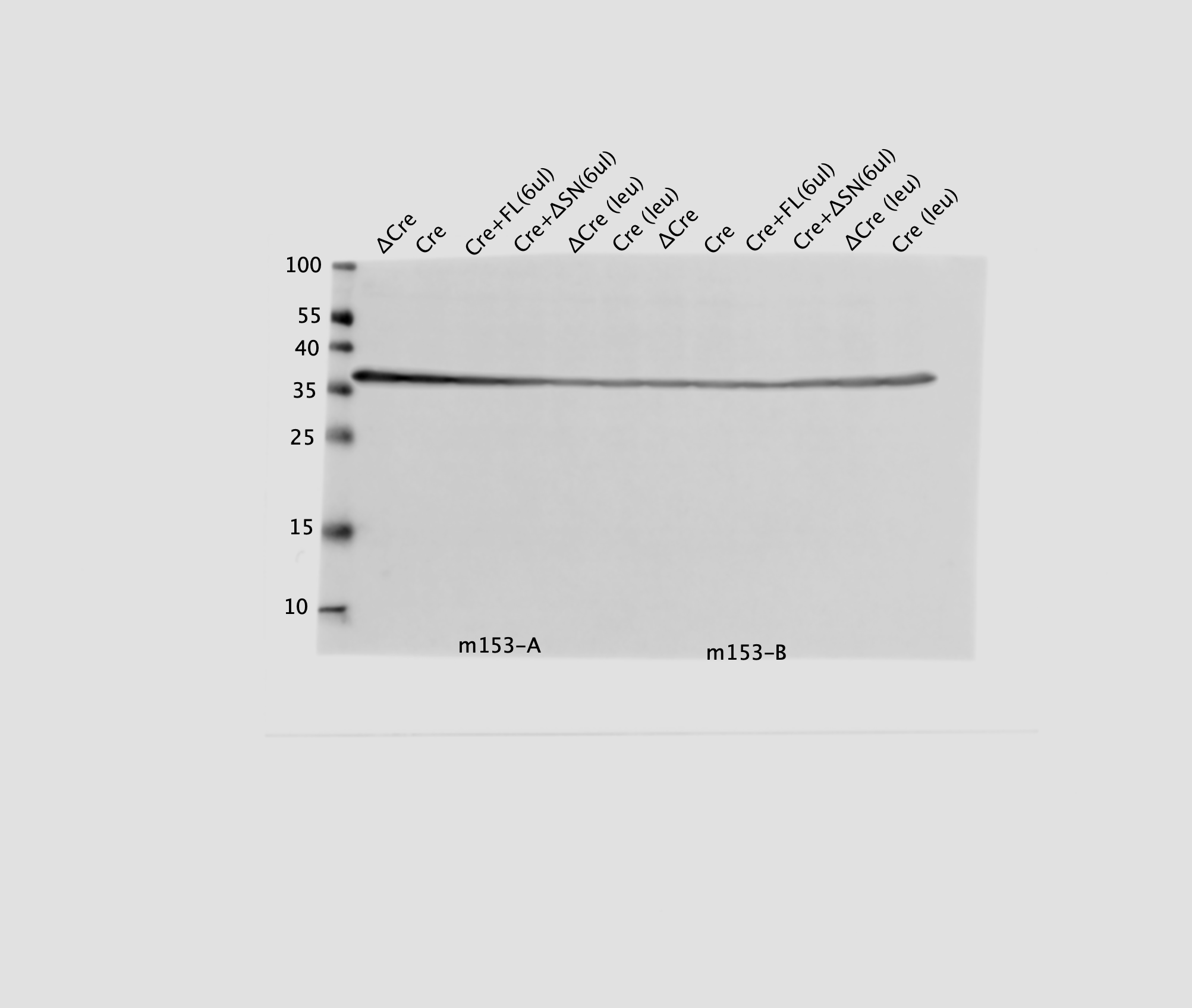

Supplement: Figure 2—source data 1. [file elife-85561-fig2-data1.zip › Figure 2_source files/Figure 2G_source files/Gapdh_memb1_ladder.tif]

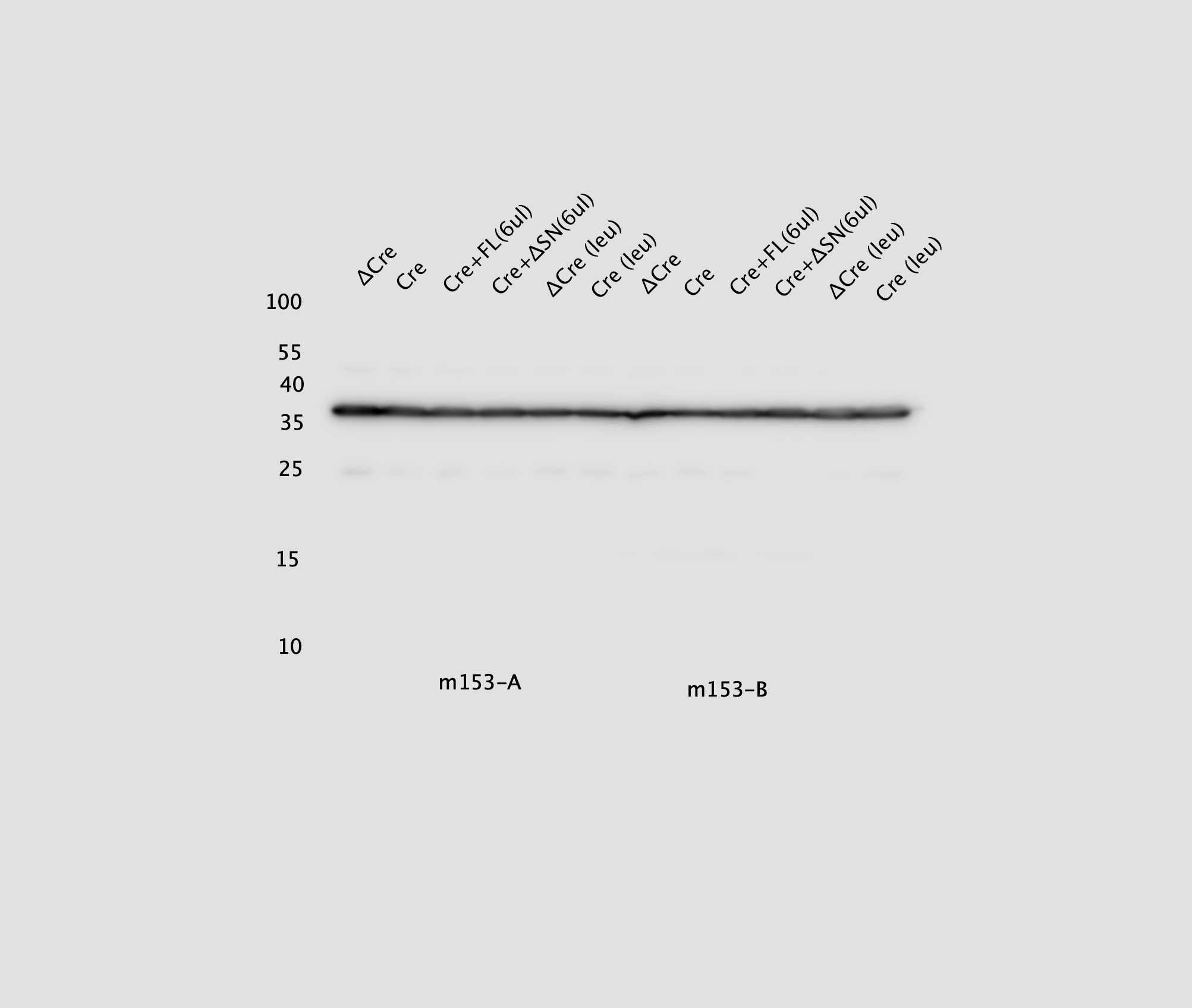

Supplement: Figure 2—source data 1. [file elife-85561-fig2-data1.zip › Figure 2_source files/Figure 2G_source files/Gapdh_memb2.tif]

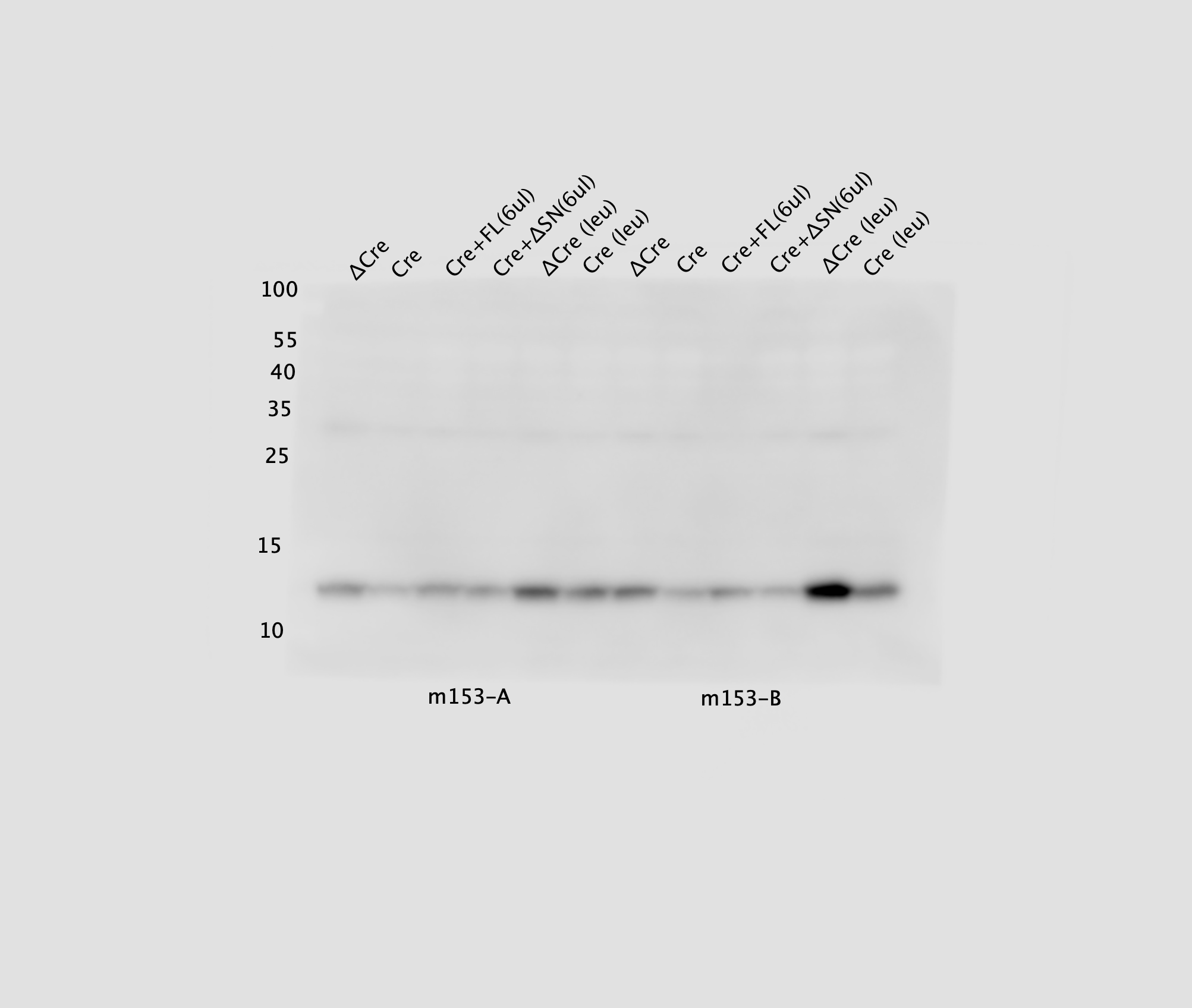

Supplement: Figure 2—source data 1. [file elife-85561-fig2-data1.zip › Figure 2_source files/Figure 2G_source files/BDNF_memb2.tif]

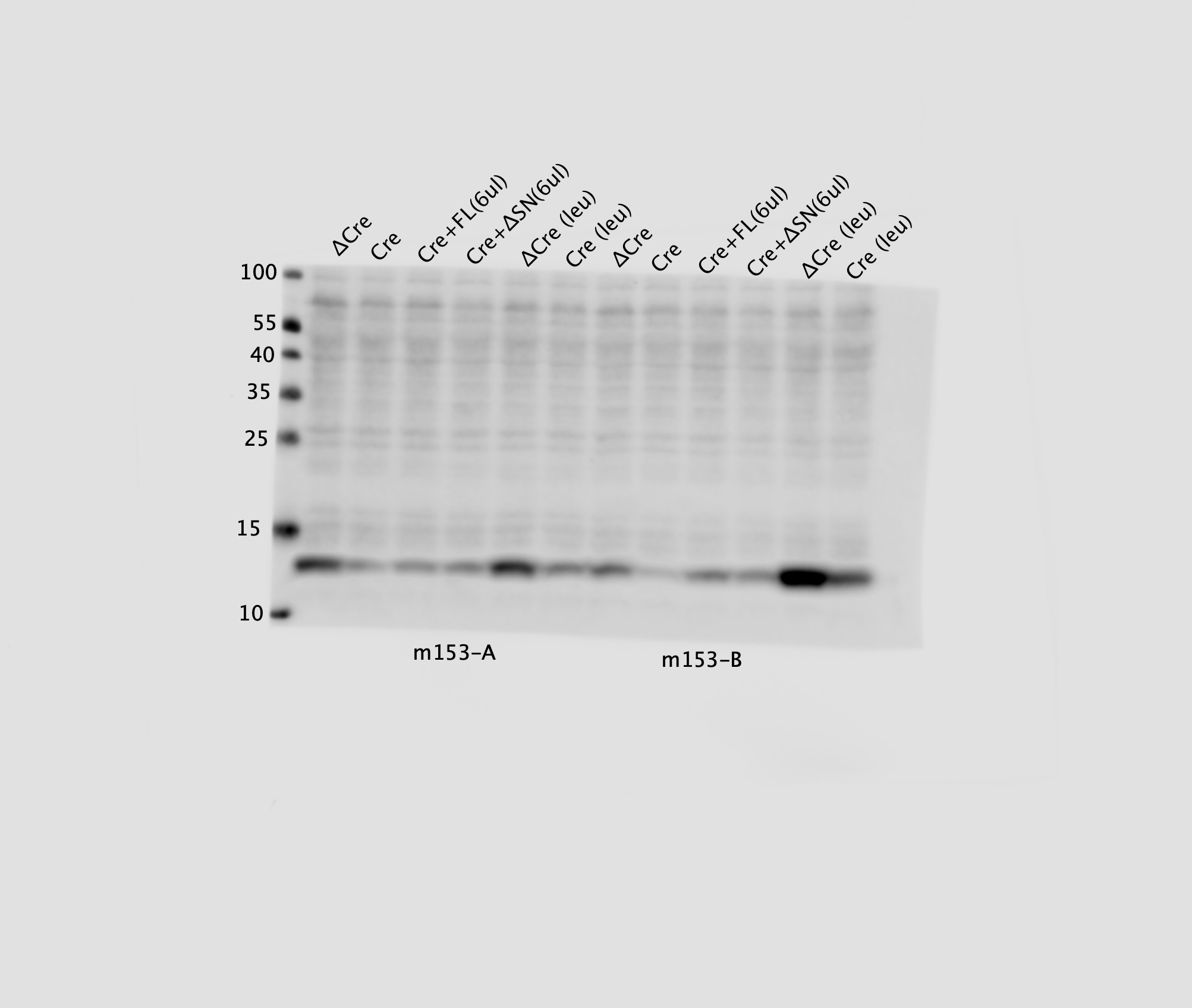

Supplement: Figure 2—source data 1. [file elife-85561-fig2-data1.zip › Figure 2_source files/Figure 2G_source files/BDNF_memb1_ladder.tif]

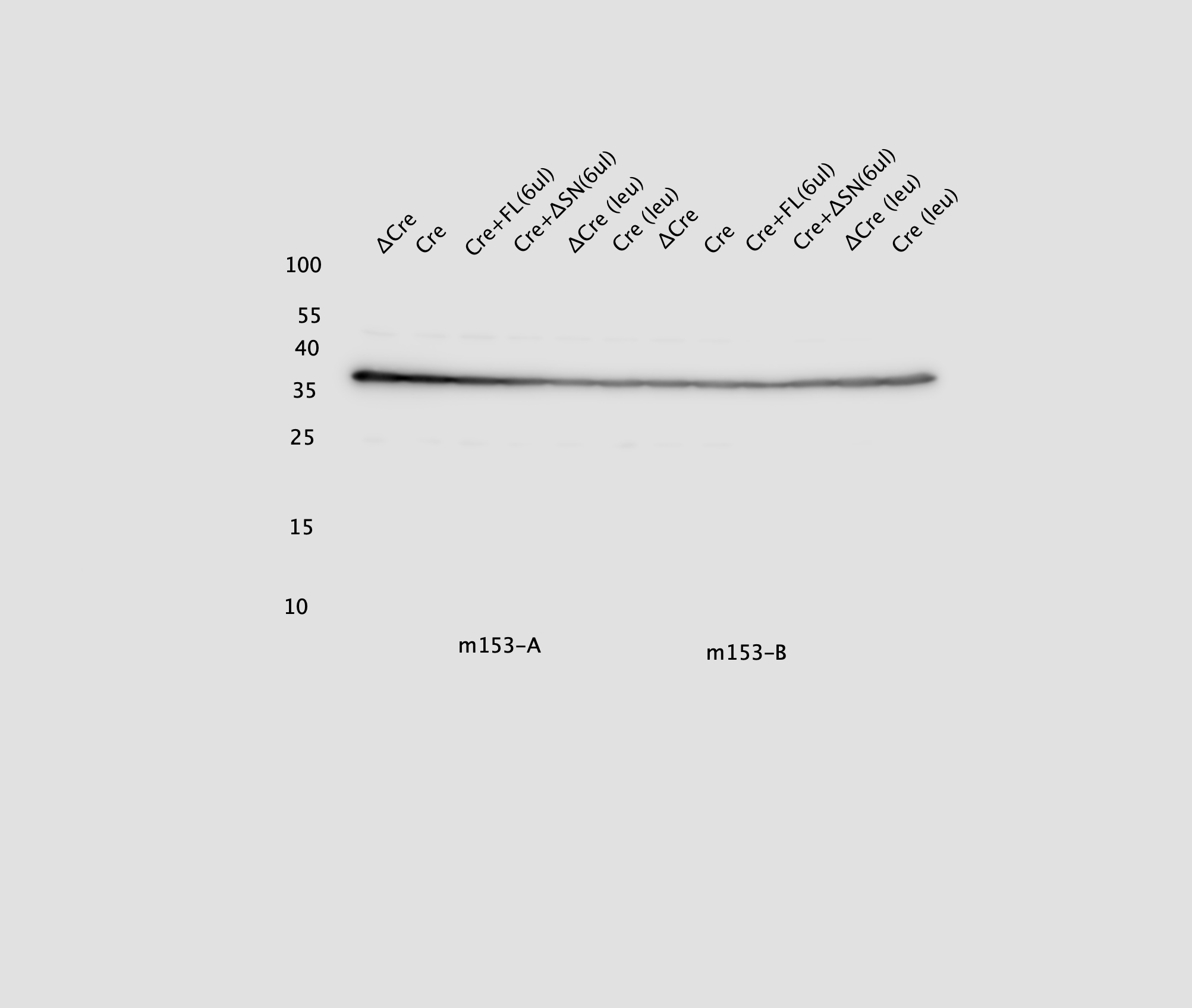

Supplement: Figure 2—source data 1. [file elife-85561-fig2-data1.zip › Figure 2_source files/Figure 2G_source files/Gapdh_memb1.tif]

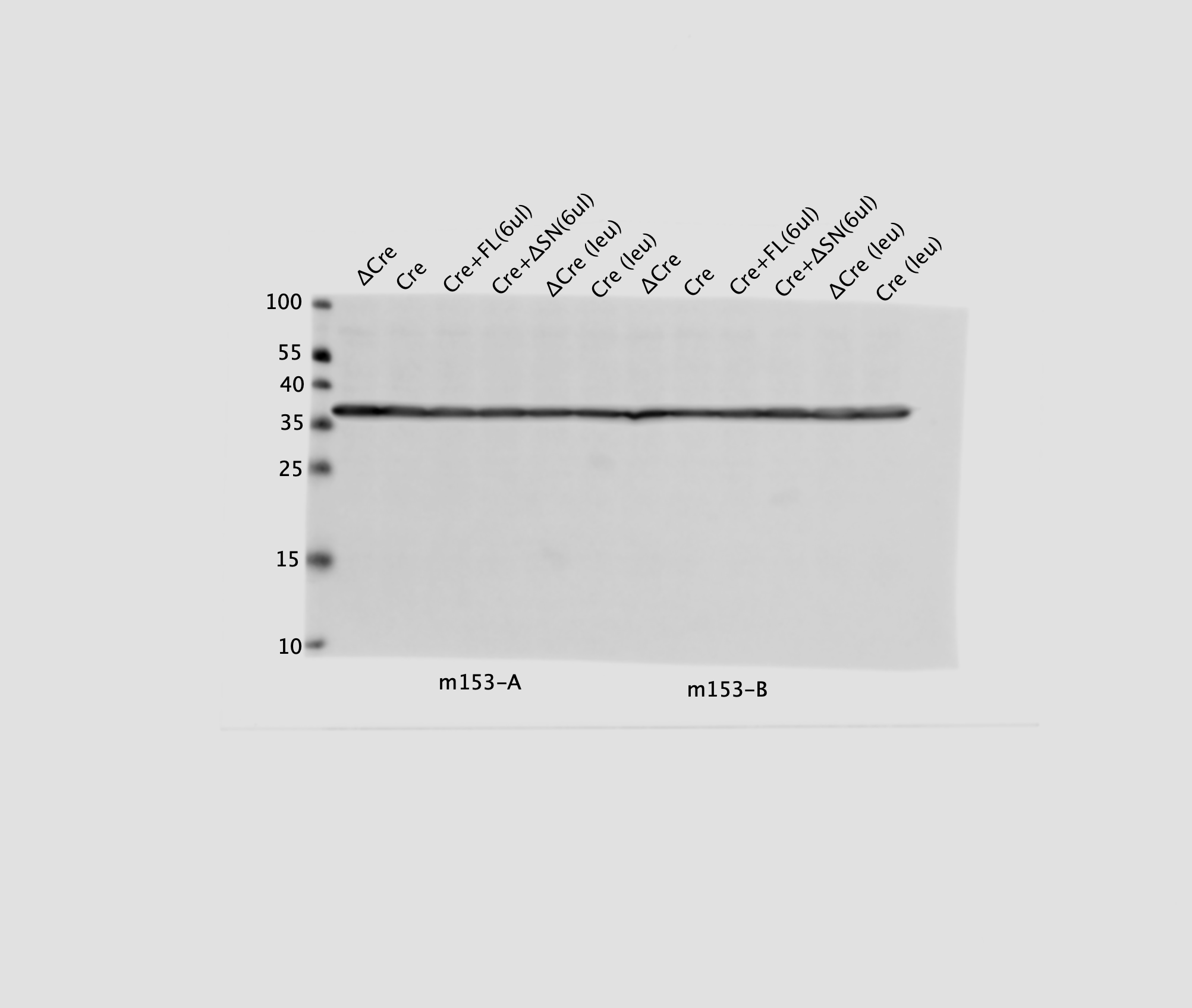

Supplement: Figure 2—source data 1. [file elife-85561-fig2-data1.zip › Figure 2_source files/Figure 2G_source files/Gapdh_memb2_ladder.tif]

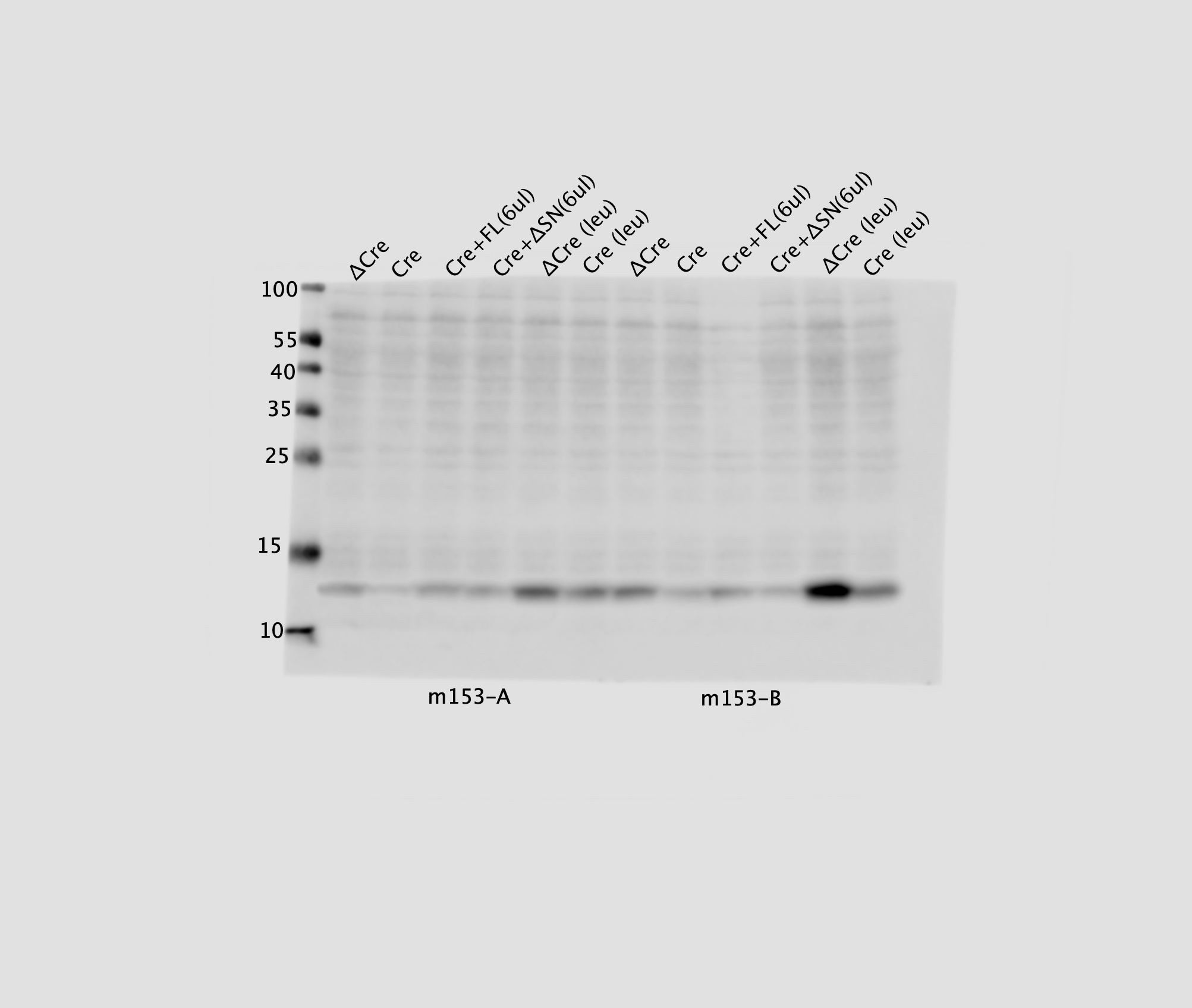

Supplement: Figure 2—source data 1. [file elife-85561-fig2-data1.zip › Figure 2_source files/Figure 2G_source files/BDNF_memb2_ladder.tif]

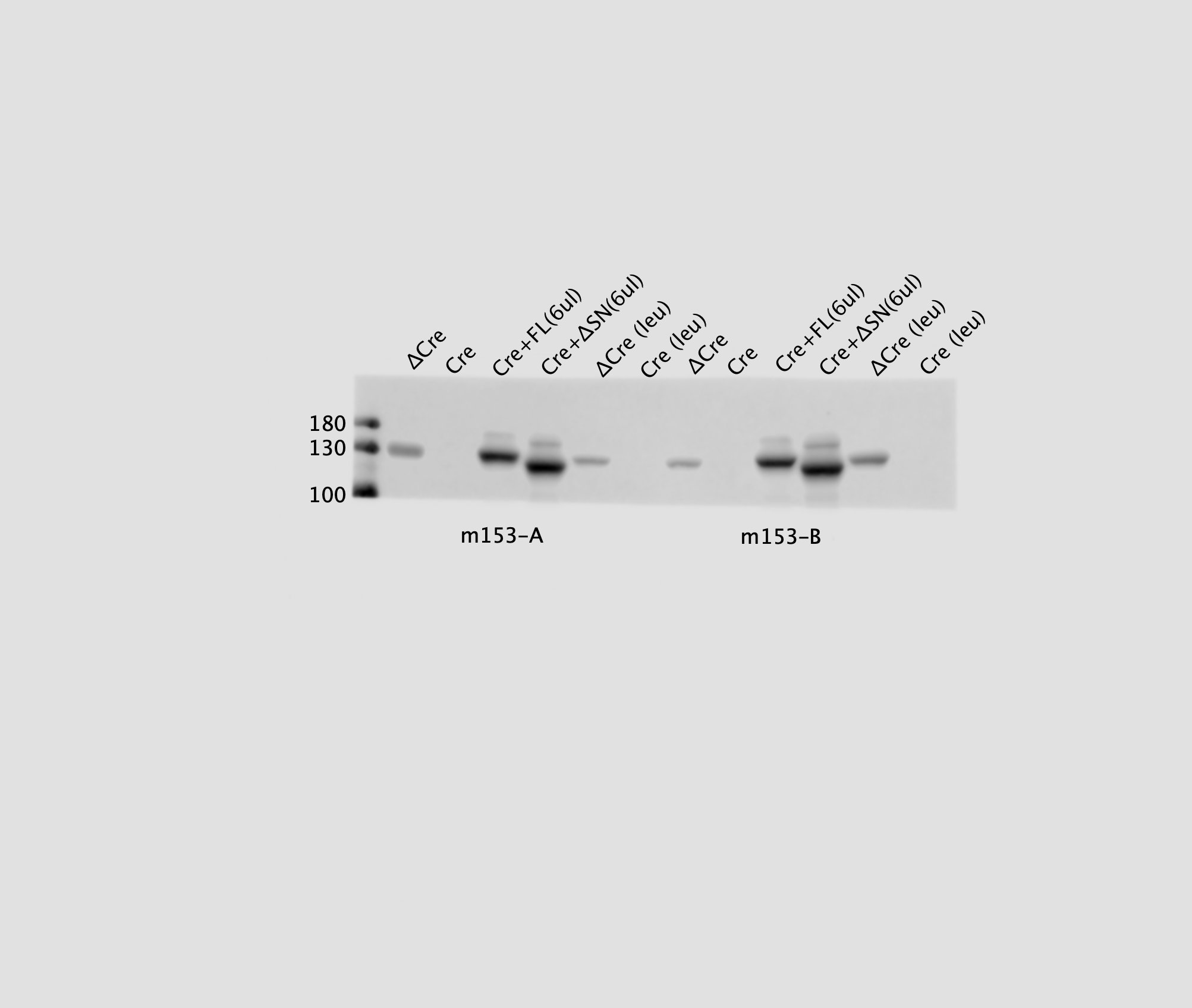

Supplement: Figure 2—source data 1. [file elife-85561-fig2-data1.zip › Figure 2_source files/Figure 2G_source files/tomosyn_ladder.tif]

## BDNF

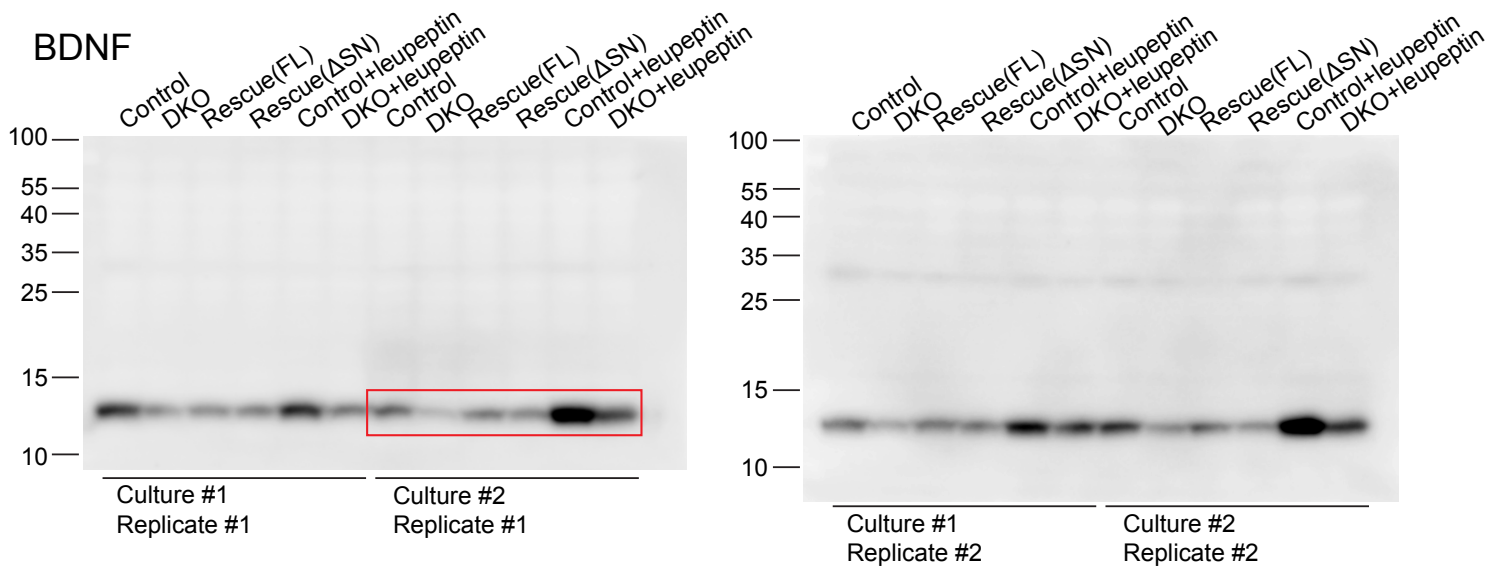

## Tomosyn (STXBP5)

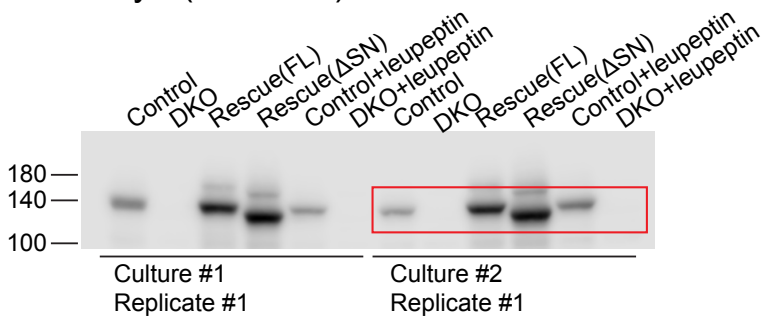

## GAPDH

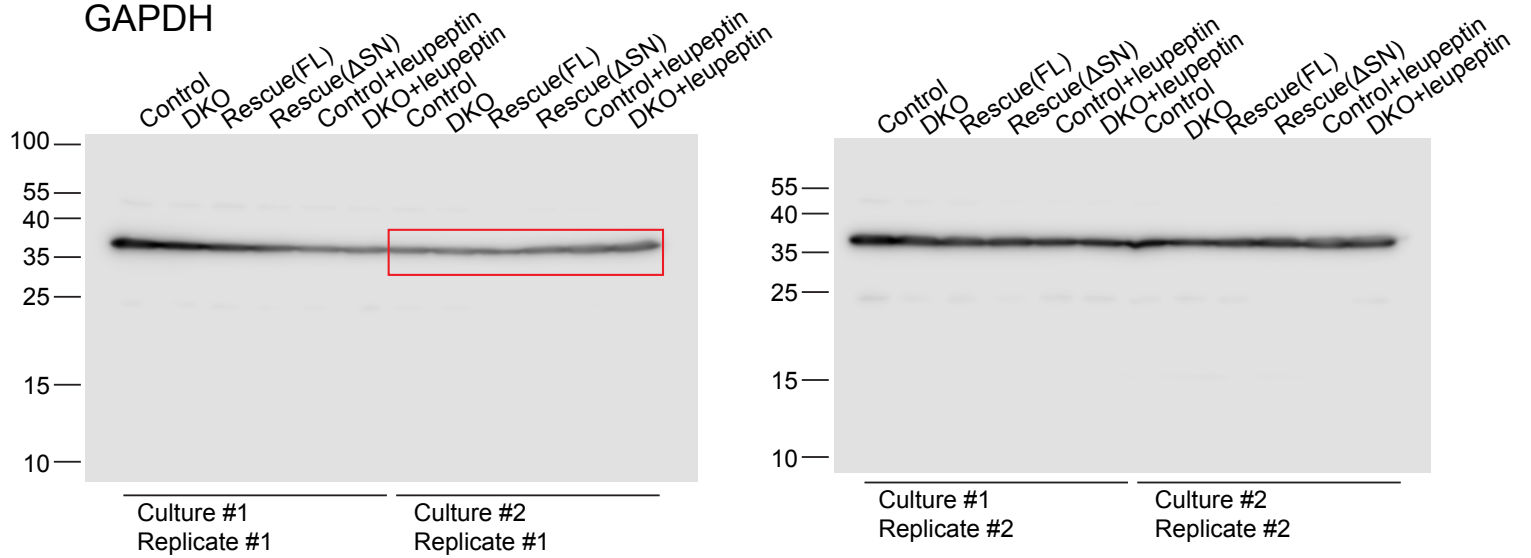

Supplement: Figure 2—source data 1. [file elife-85561-fig2-data1.zip › Figure 2_source files/Figure 2G_source files/Figure 2G_uncropped blots.pdf]

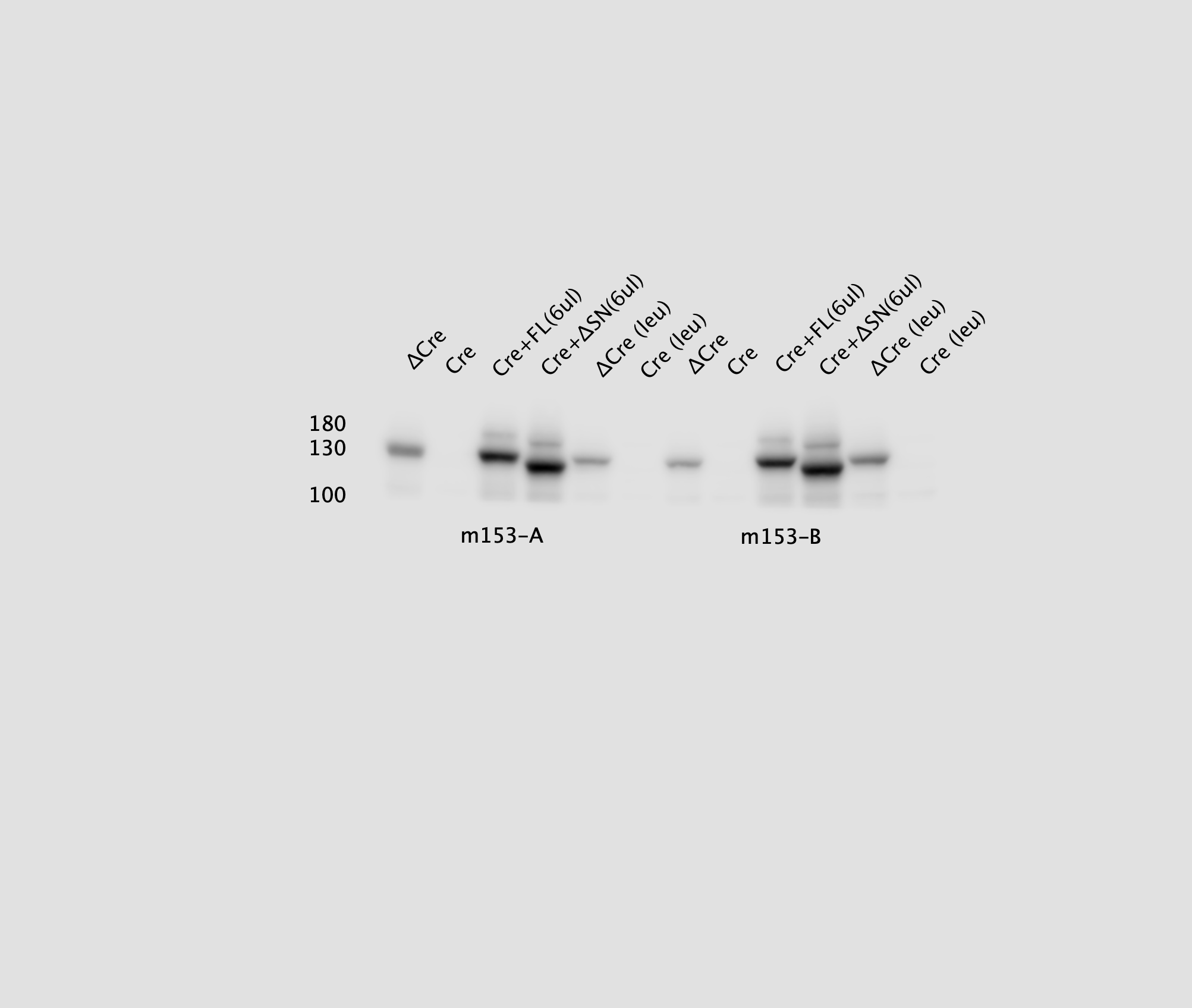

Supplement: Figure 2—source data 1. [file elife-85561-fig2-data1.zip › Figure 2_source files/Figure 2G_source files/tomosyn.tif]

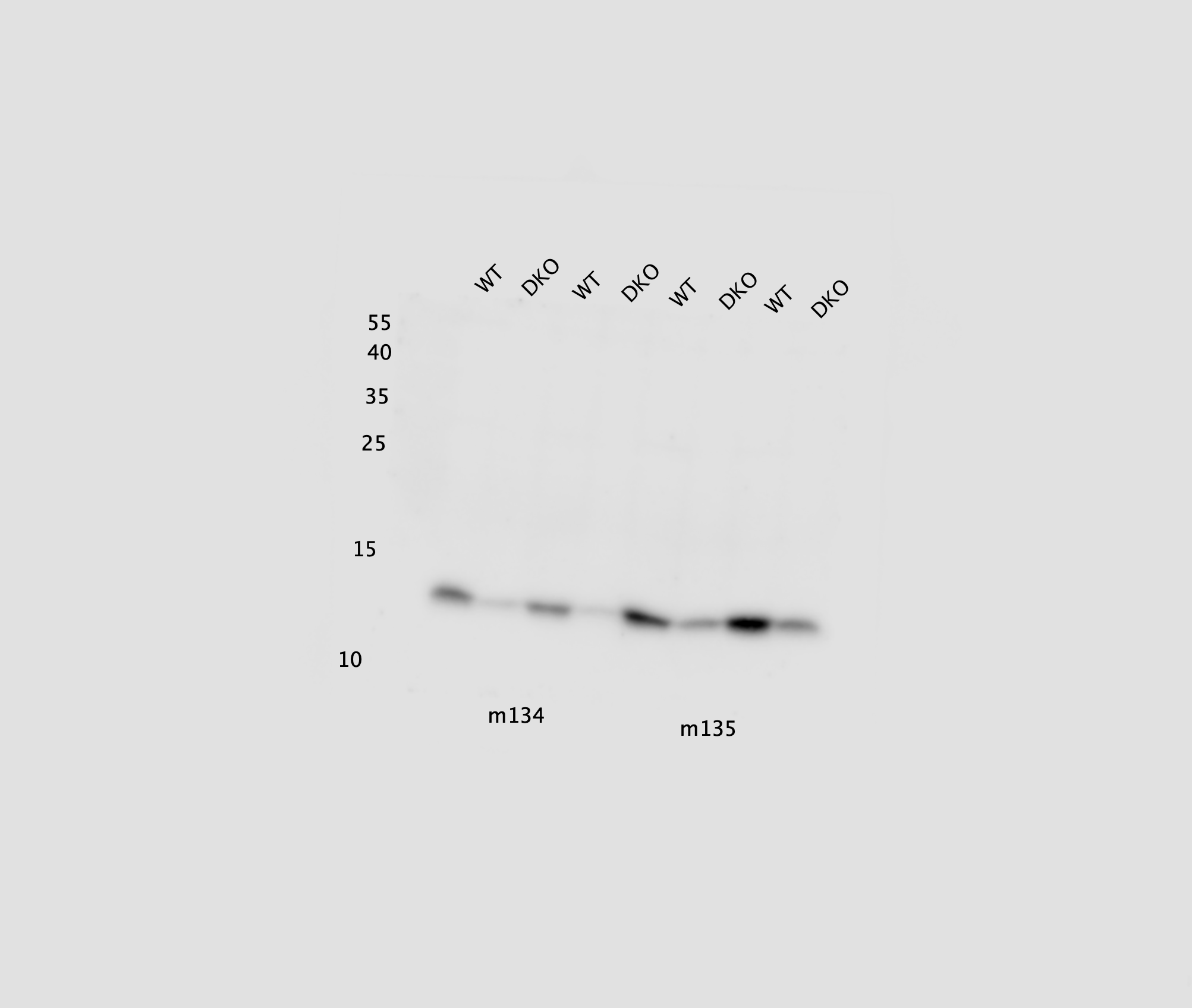

Supplement: Figure 2—source data 1. [file elife-85561-fig2-data1.zip › Figure 2_source files/Figure 2E_source files/BDNF_replicates3-4.tif]

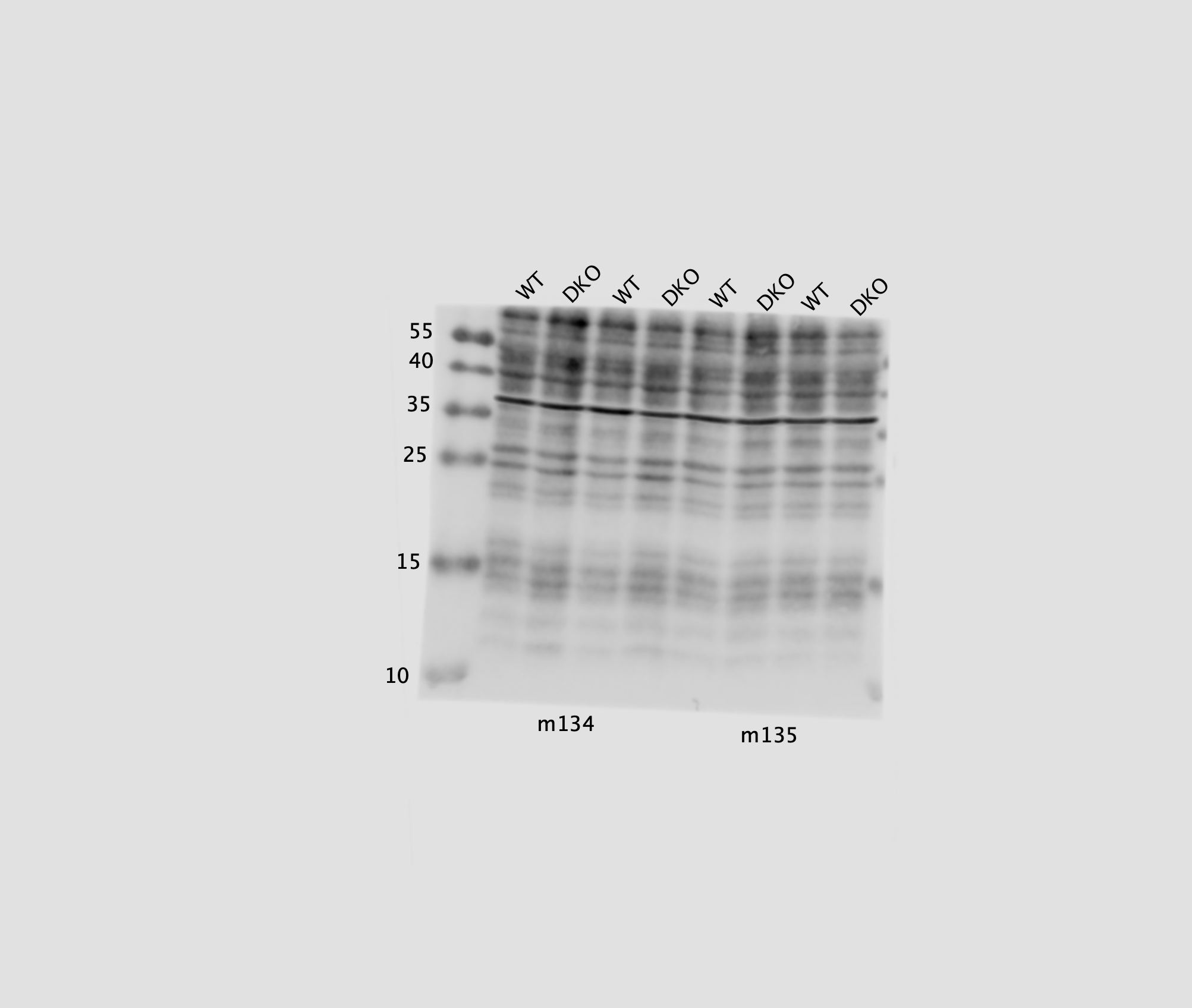

Supplement: Figure 2—source data 1. [file elife-85561-fig2-data1.zip › Figure 2_source files/Figure 2E_source files/GAPDH_replicates3-4_ladder.tif]

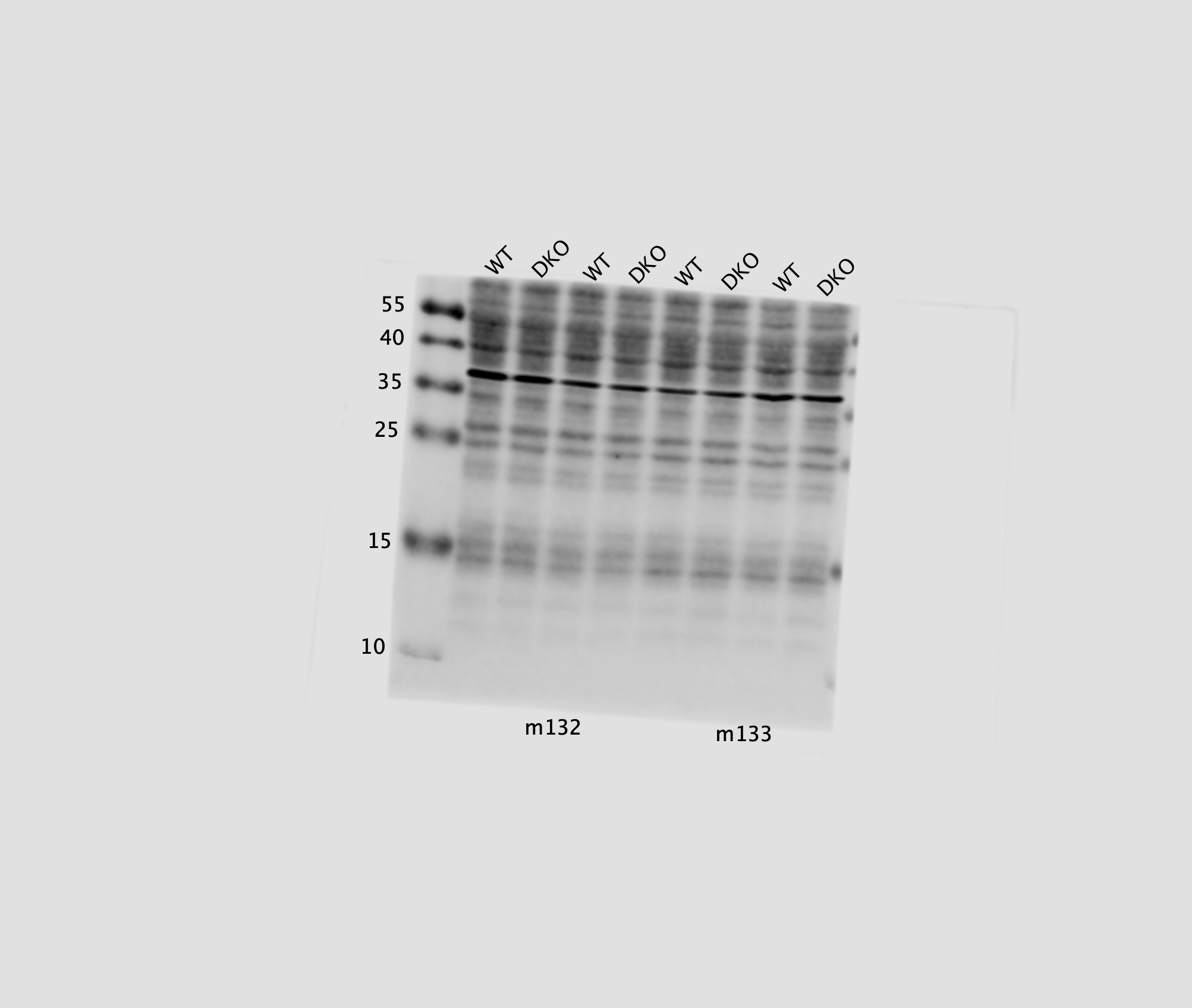

Supplement: Figure 2—source data 1. [file elife-85561-fig2-data1.zip › Figure 2_source files/Figure 2E_source files/GAPDH_replicates1-2_ladder.tif]

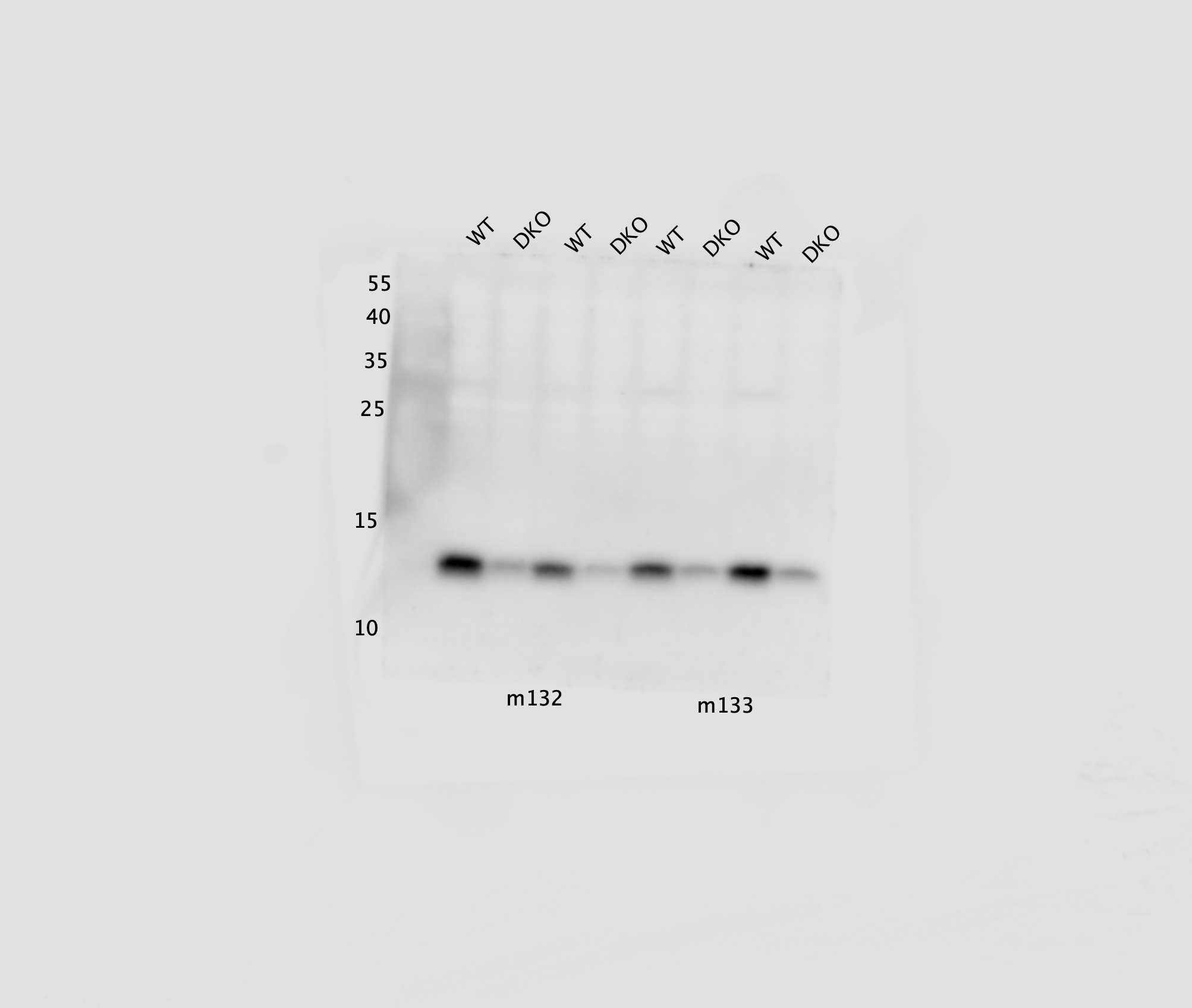

Supplement: Figure 2—source data 1. [file elife-85561-fig2-data1.zip › Figure 2_source files/Figure 2E_source files/BDNF_replicates1-2.tif]

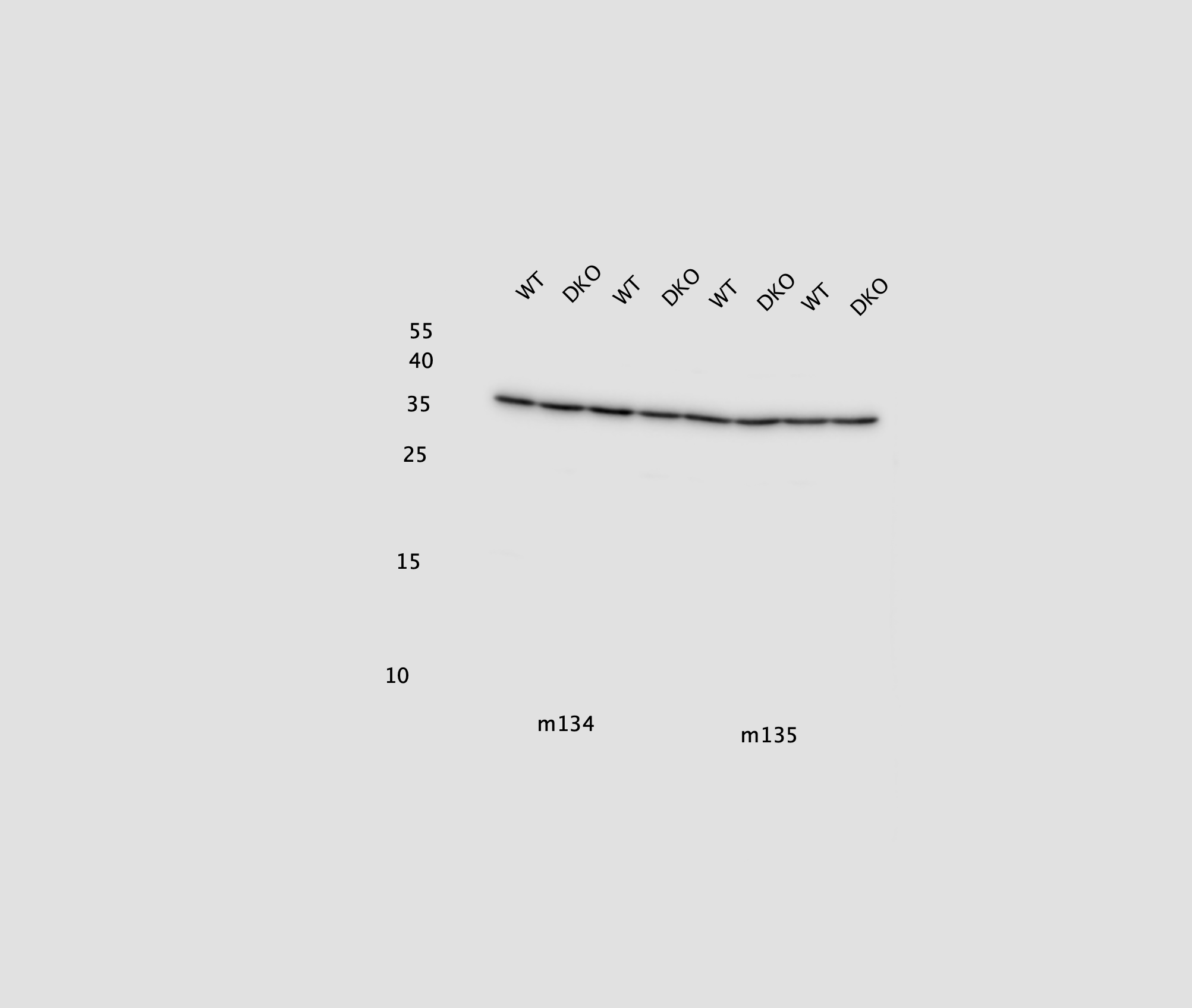

Supplement: Figure 2—source data 1. [file elife-85561-fig2-data1.zip › Figure 2_source files/Figure 2E_source files/GAPDH_replicates3-4.tif]

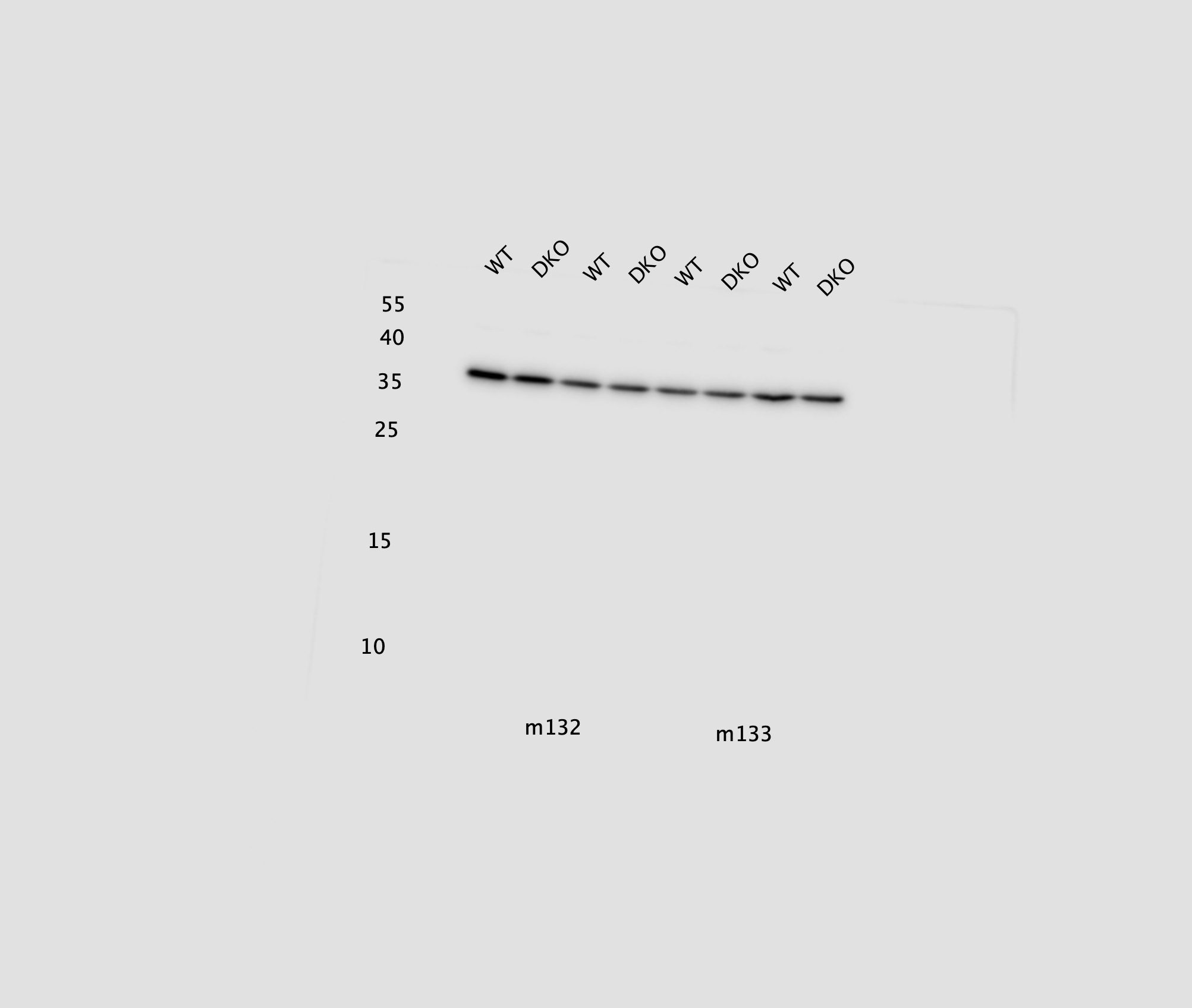

Supplement: Figure 2—source data 1. [file elife-85561-fig2-data1.zip › Figure 2_source files/Figure 2E_source files/GAPDH_replicates1-2.tif]

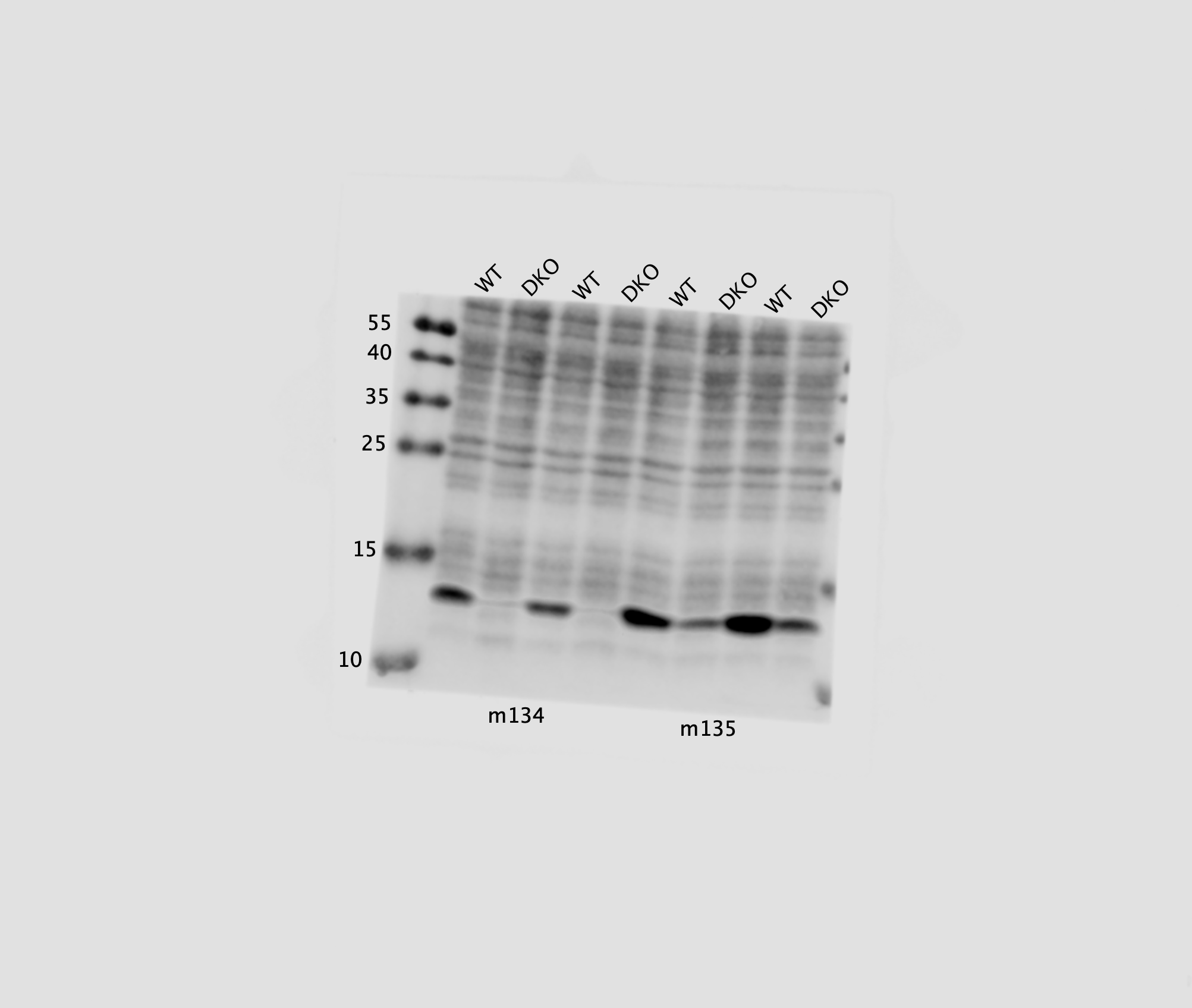

Supplement: Figure 2—source data 1. [file elife-85561-fig2-data1.zip › Figure 2_source files/Figure 2E_source files/BDNF_replicates3-4_ladder.tif]

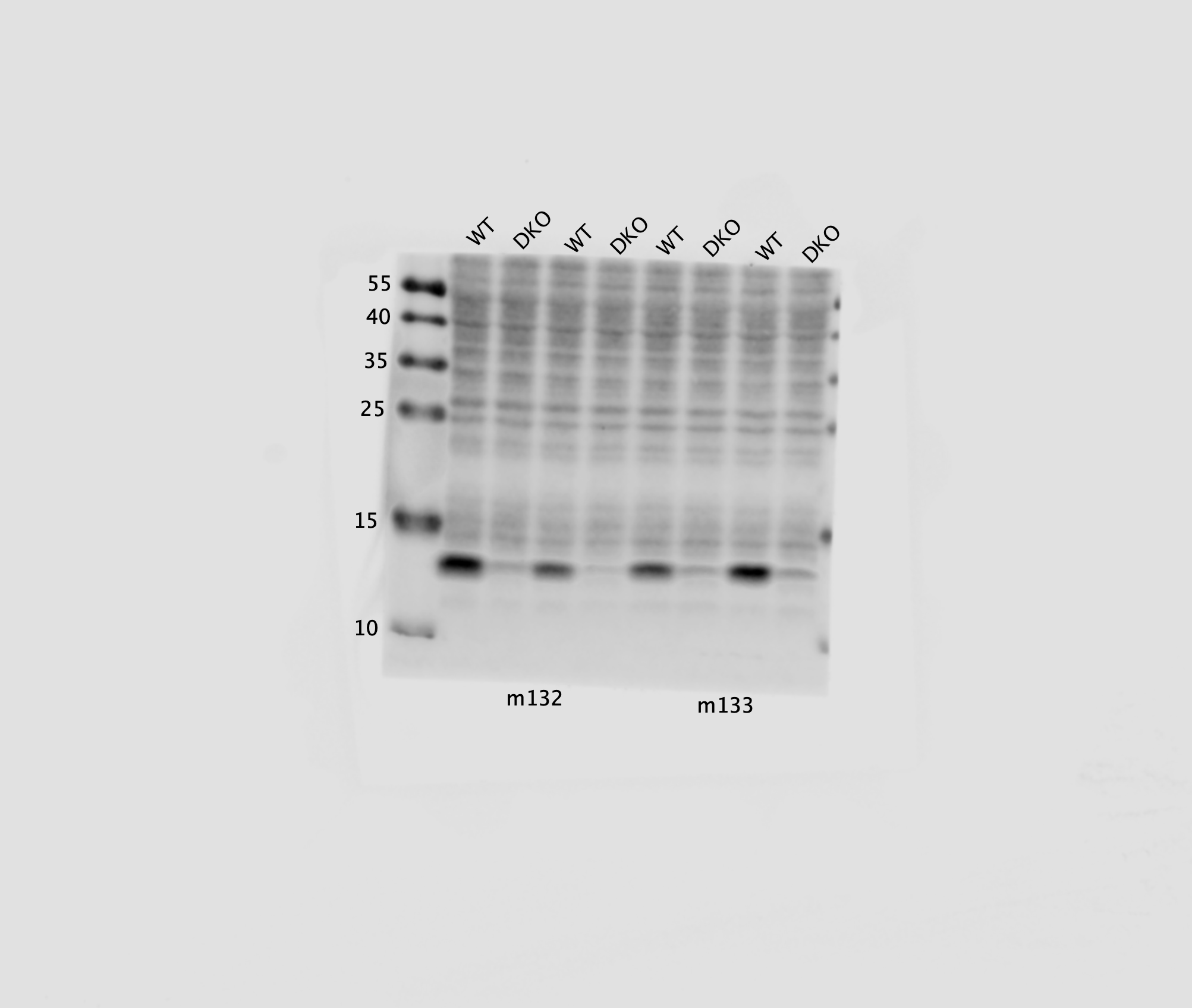

Supplement: Figure 2—source data 1. [file elife-85561-fig2-data1.zip › Figure 2_source files/Figure 2E_source files/BDNF_replicates1-2_ladder.tif]

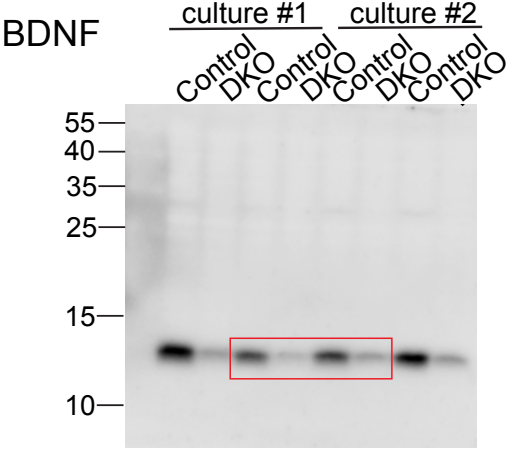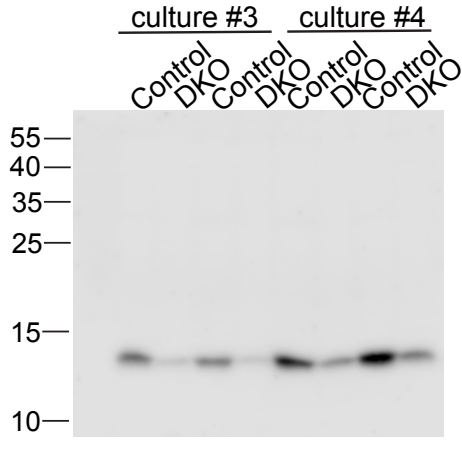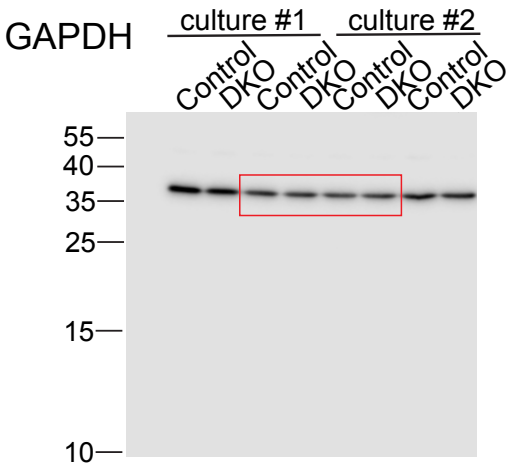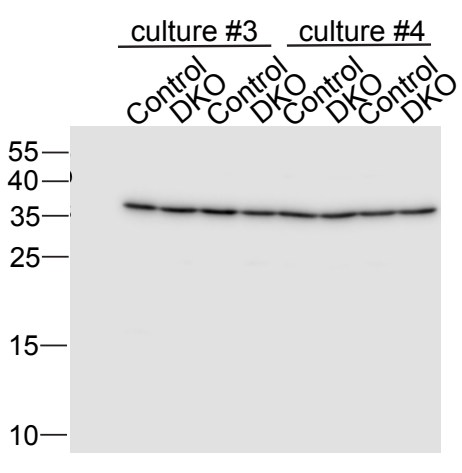

Supplement: Figure 2—source data 1. [file elife-85561-fig2-data1.zip › Figure 2_source files/Figure 2E_source files/Figure 2E_uncropped blots.pdf]

IA-2 (PTPRN)

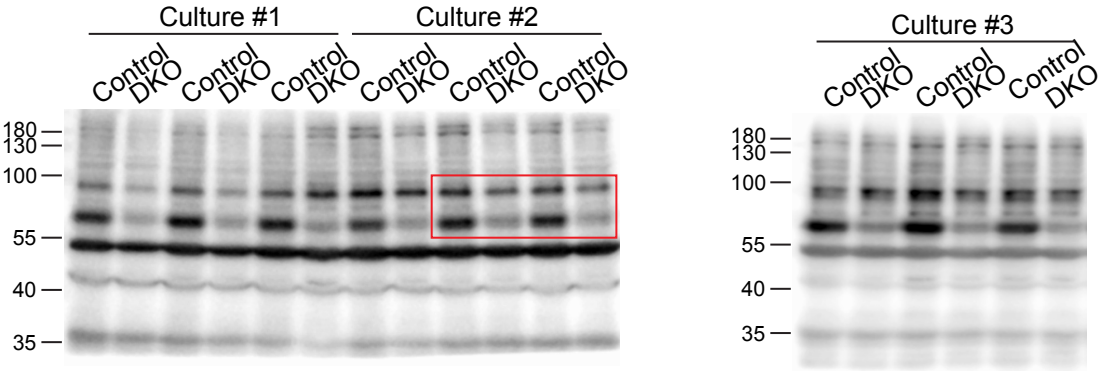

Total protein stain

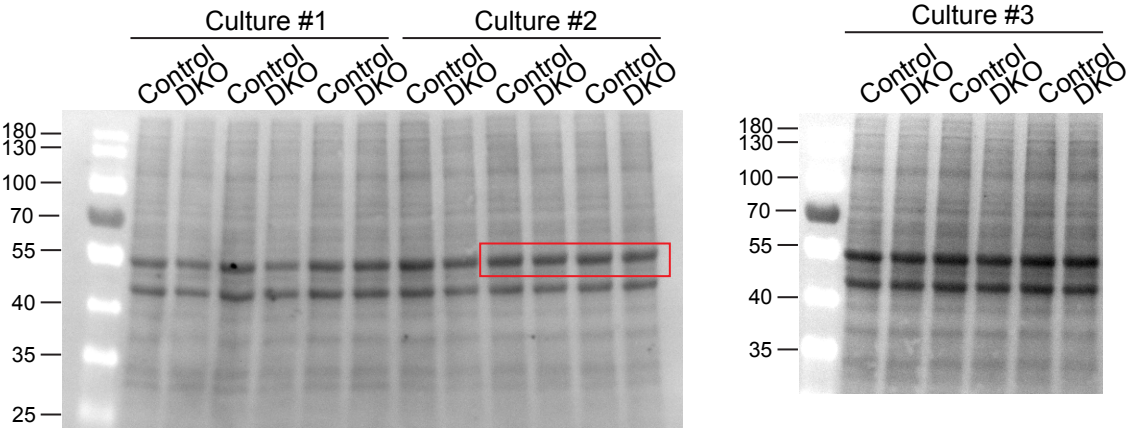

Actin

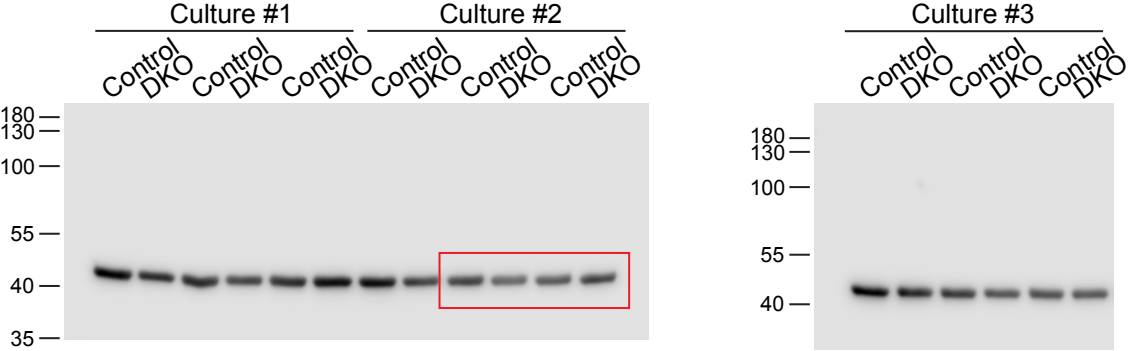

Supplement: Figure 2—figure supplement 2—source data 1. [file elife-85561-fig2-figsupp2-data1.zip › Figure 2 - figure supplement 2_source files/Figure 2 - figure supplement 2_uncropped blots.pdf]

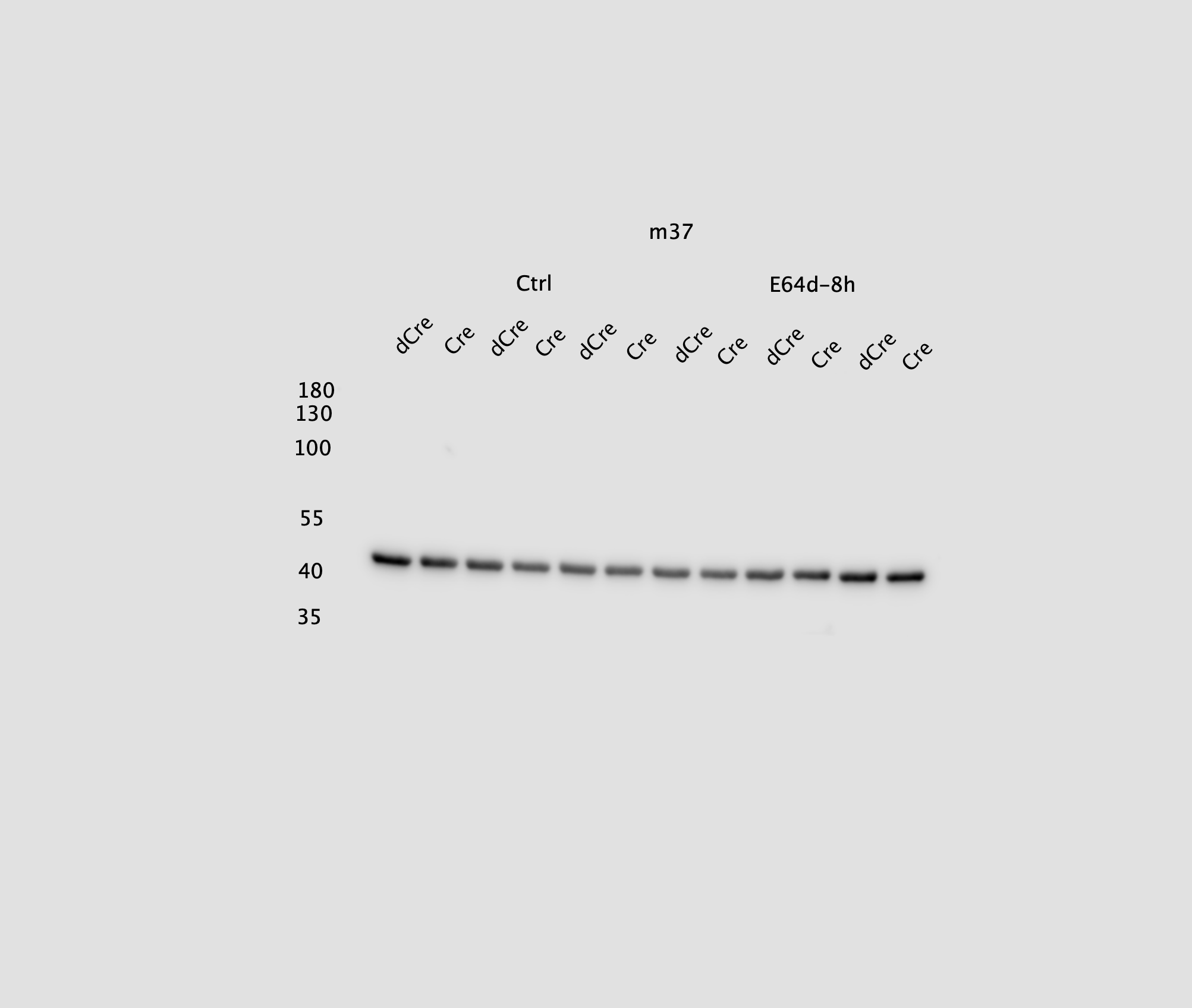

Supplement: Figure 2—figure supplement 2—source data 1. [file elife-85561-fig2-figsupp2-data1.zip › Figure 2 - figure supplement 2_source files/actin_replicate3.tif]

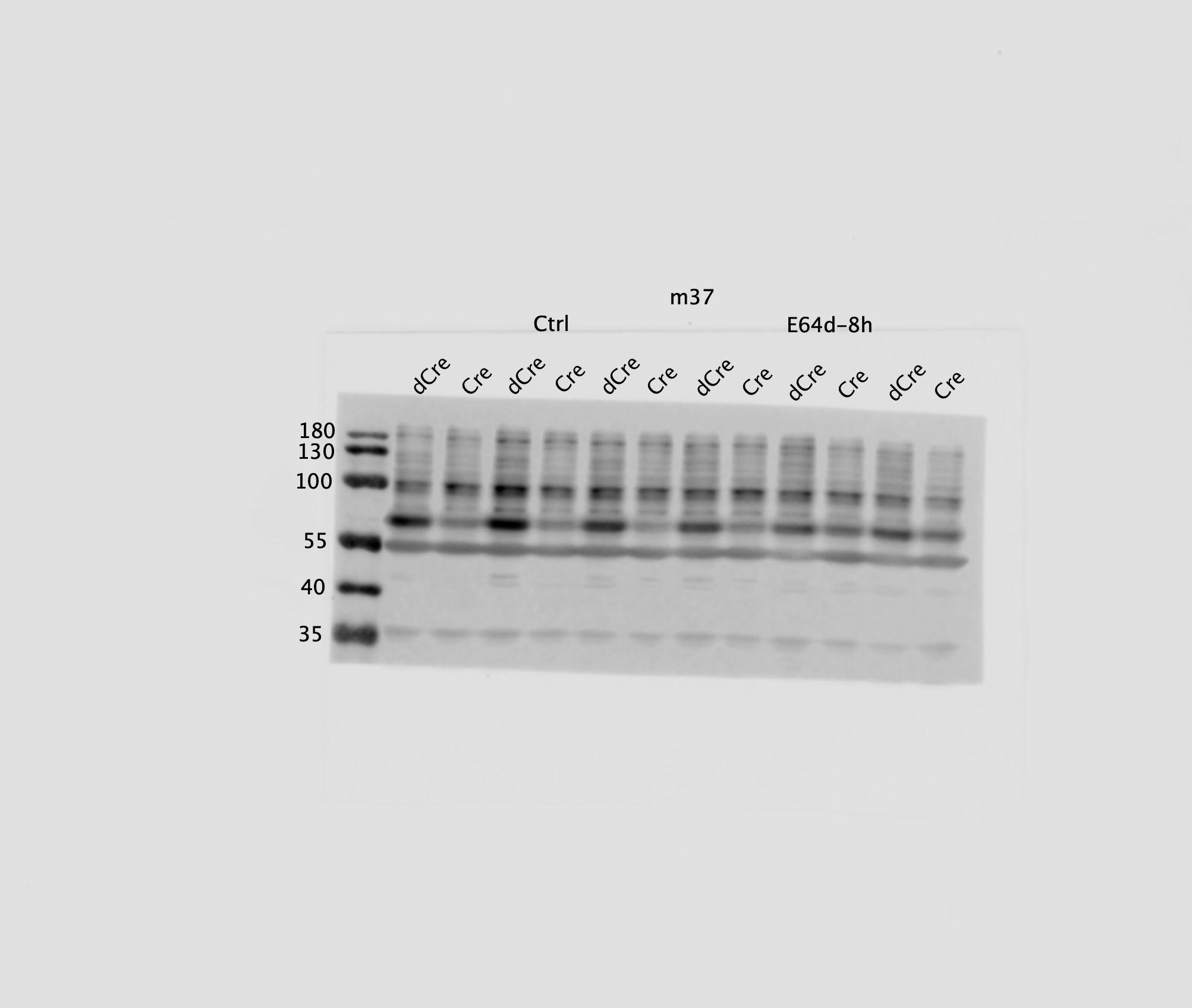

Supplement: Figure 2—figure supplement 2—source data 1. [file elife-85561-fig2-figsupp2-data1.zip › Figure 2 - figure supplement 2_source files/IA-2_replicate3_ladder.tif]

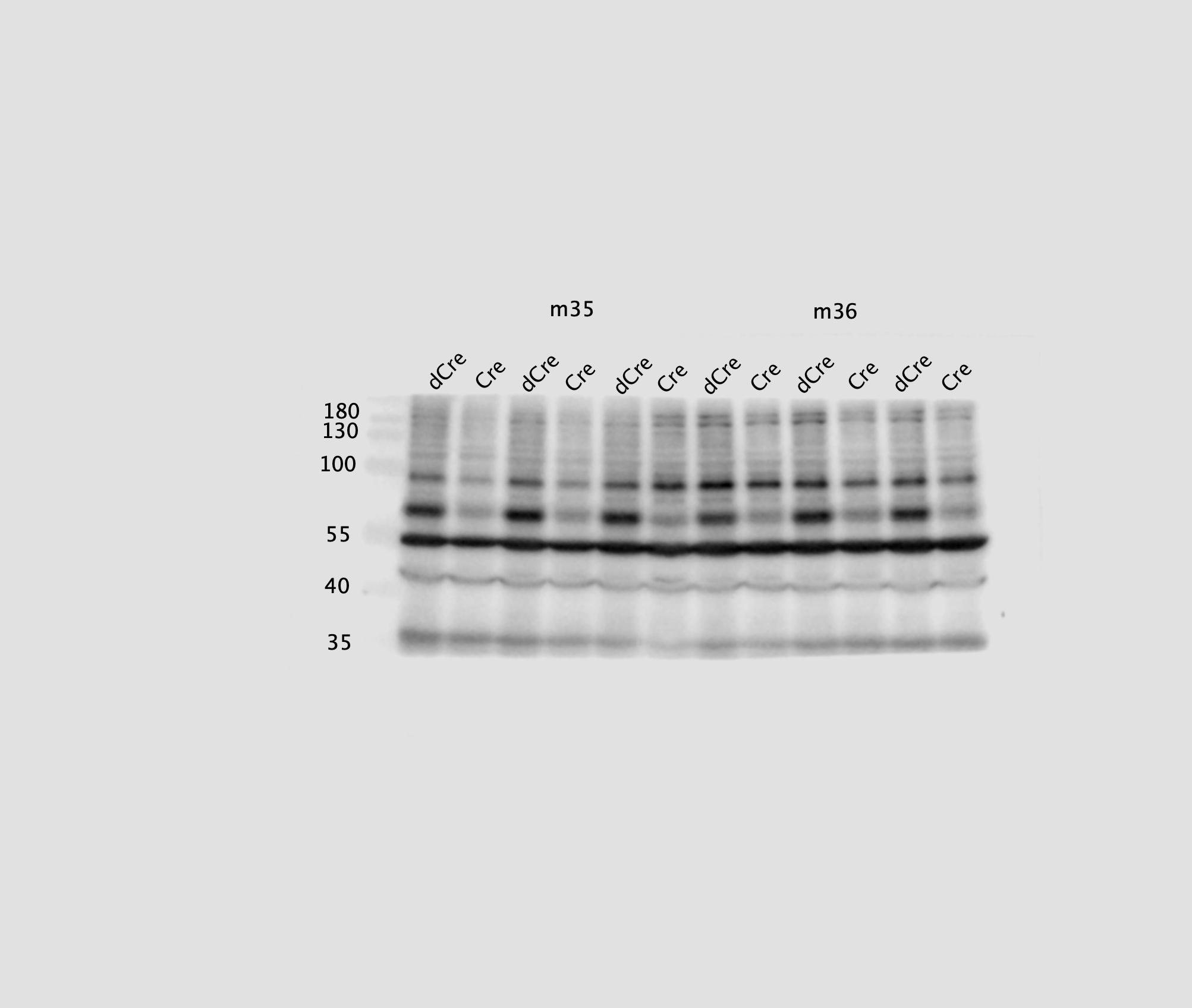

Supplement: Figure 2—figure supplement 2—source data 1. [file elife-85561-fig2-figsupp2-data1.zip › Figure 2 - figure supplement 2_source files/IA-2_replicates1-2.tif]

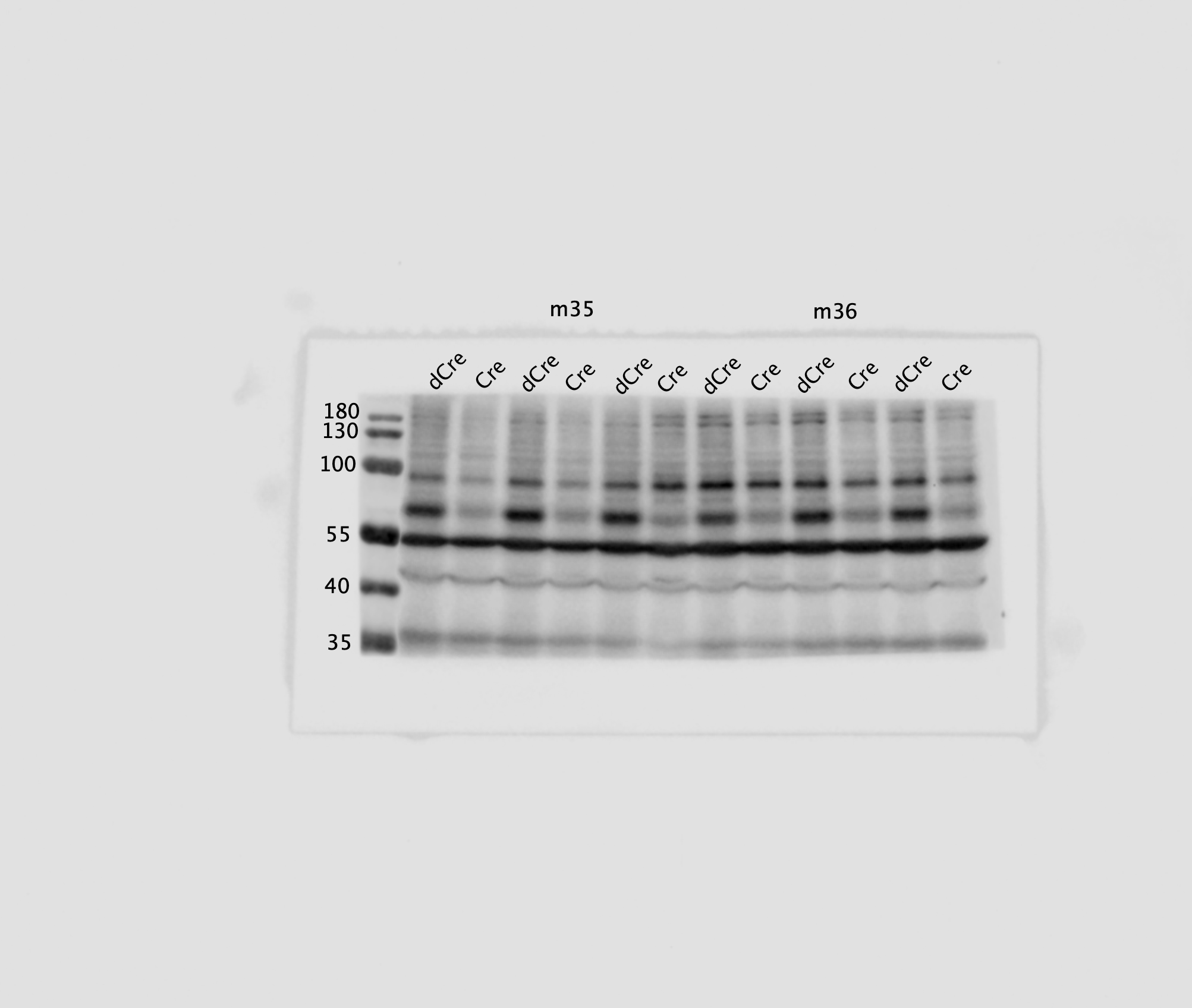

Supplement: Figure 2—figure supplement 2—source data 1. [file elife-85561-fig2-figsupp2-data1.zip › Figure 2 - figure supplement 2_source files/IA-2_replicates1-2_ladder.tif]

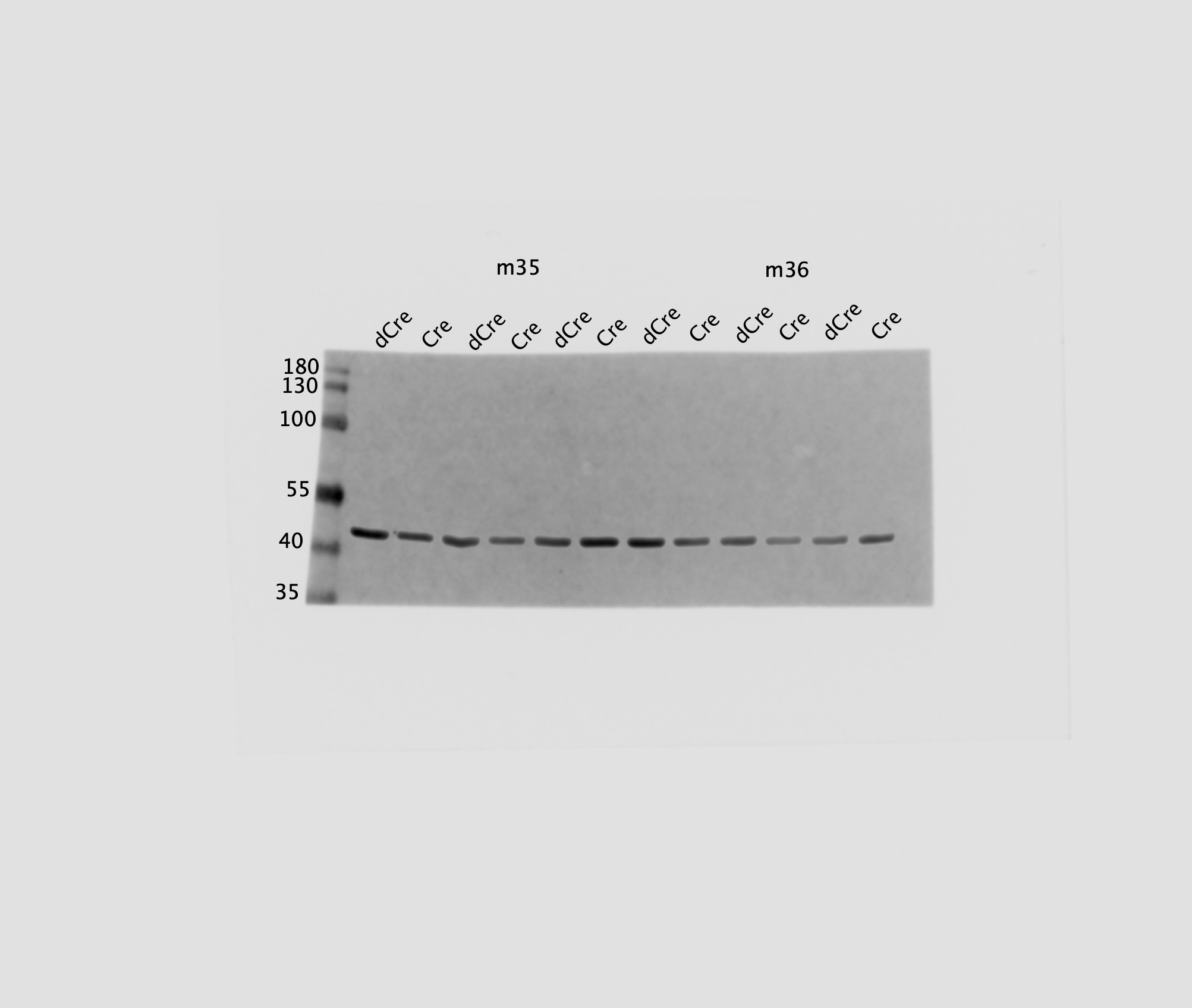

Supplement: Figure 2—figure supplement 2—source data 1. [file elife-85561-fig2-figsupp2-data1.zip › Figure 2 - figure supplement 2_source files/actin_replicates1-2_ladder.tif]

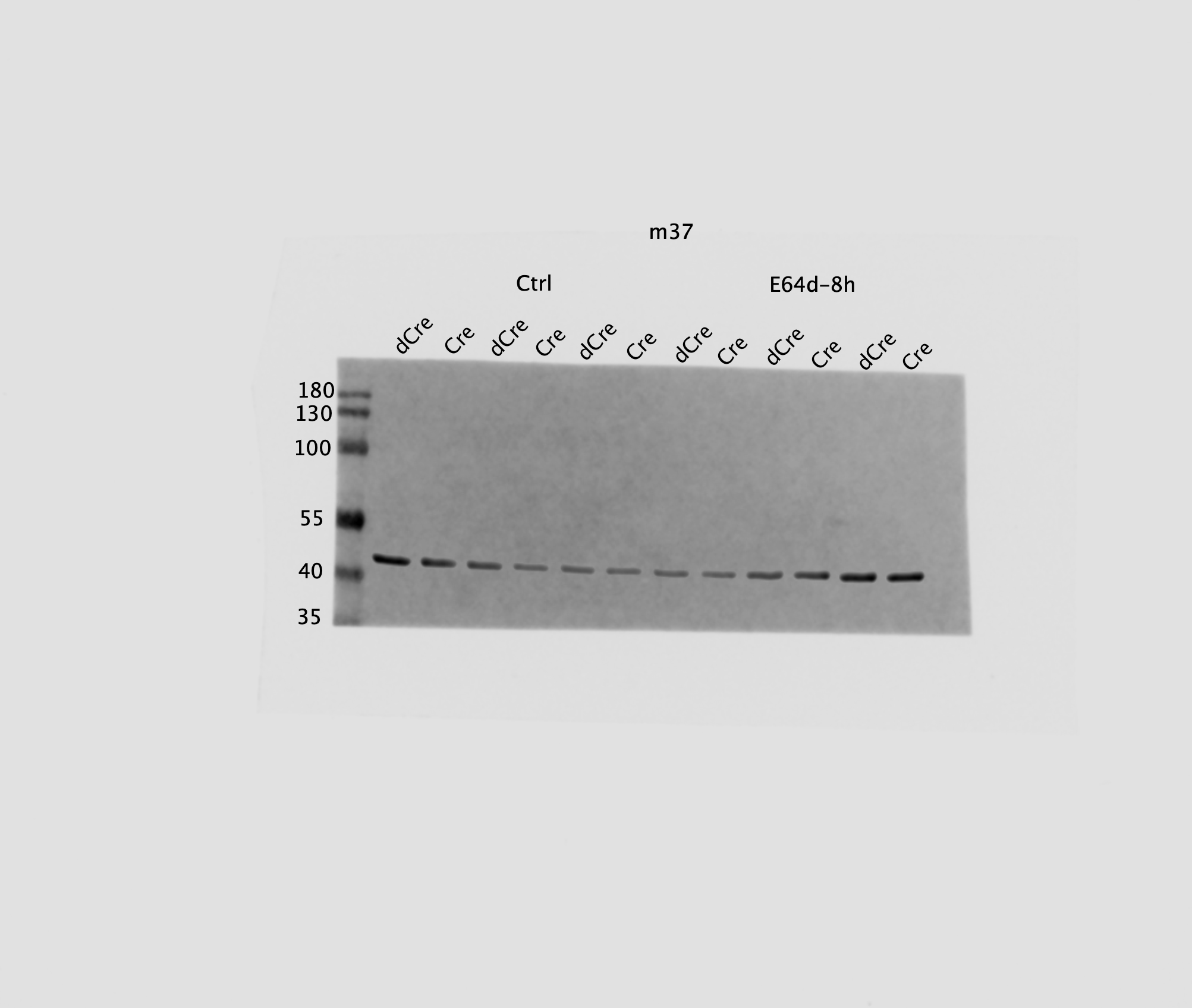

Supplement: Figure 2—figure supplement 2—source data 1. [file elife-85561-fig2-figsupp2-data1.zip › Figure 2 - figure supplement 2_source files/actin_replicate3_ladder.tif]

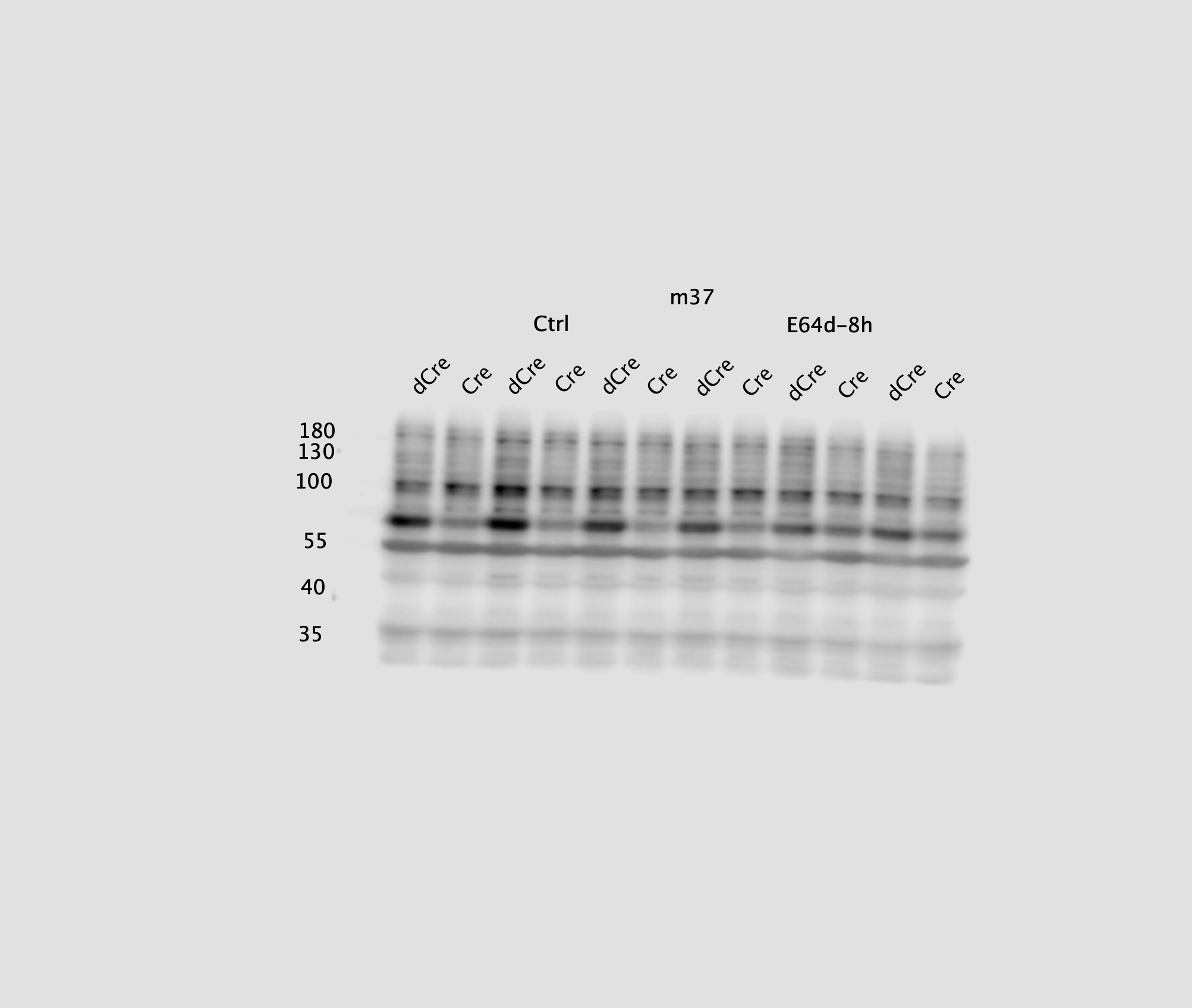

Supplement: Figure 2—figure supplement 2—source data 1. [file elife-85561-fig2-figsupp2-data1.zip › Figure 2 - figure supplement 2_source files/IA-2_replicate3.tif]

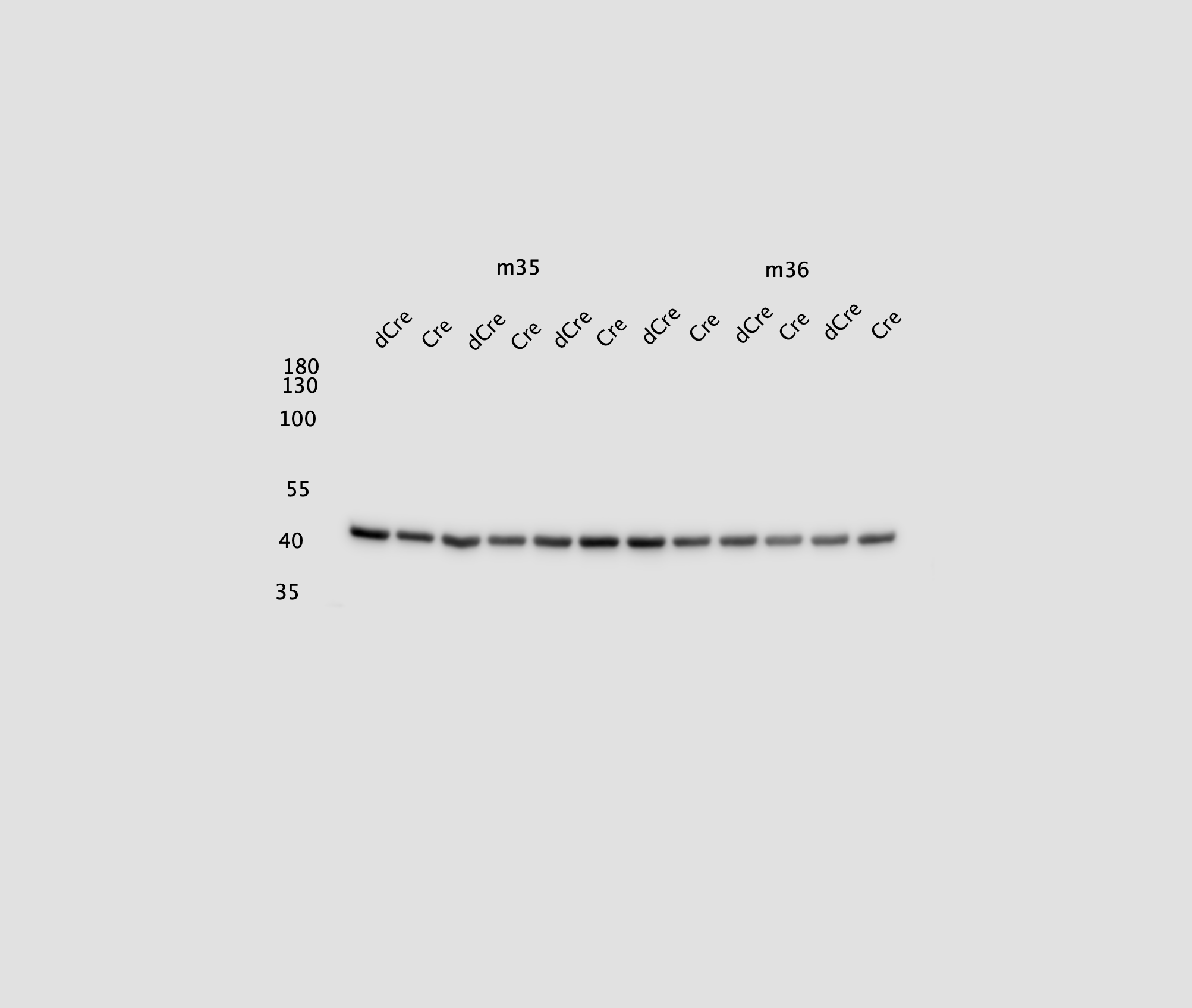

Supplement: Figure 2—figure supplement 2—source data 1. [file elife-85561-fig2-figsupp2-data1.zip › Figure 2 - figure supplement 2_source files/actin_replicates1-2.tif]

## LAMP1

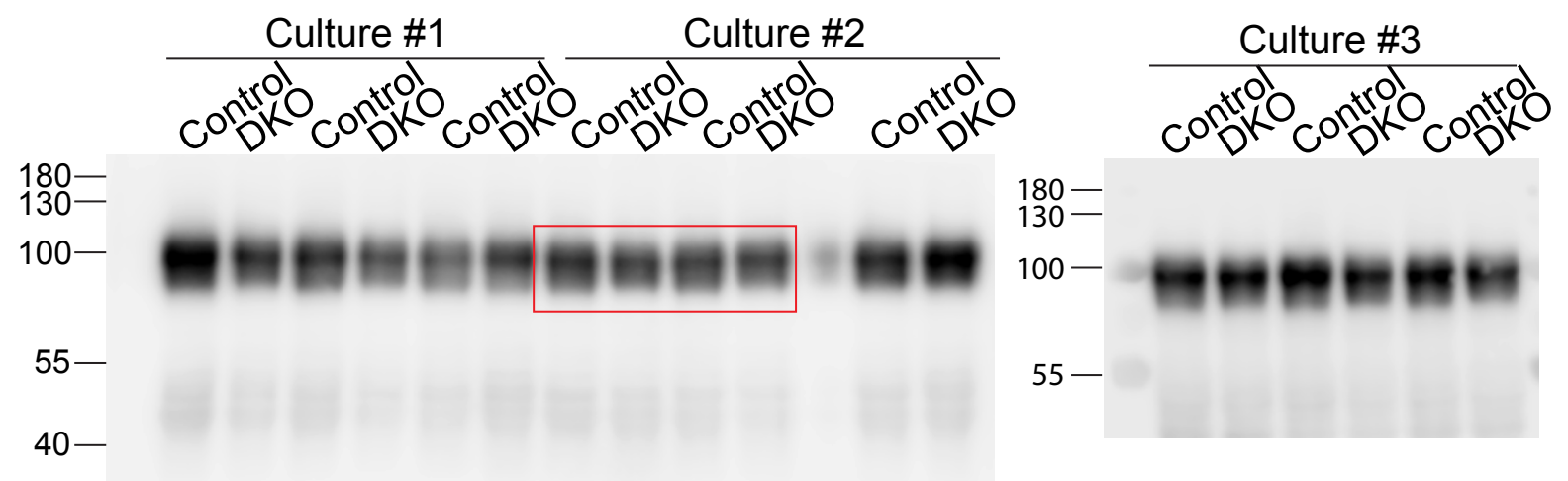

## LIMP2

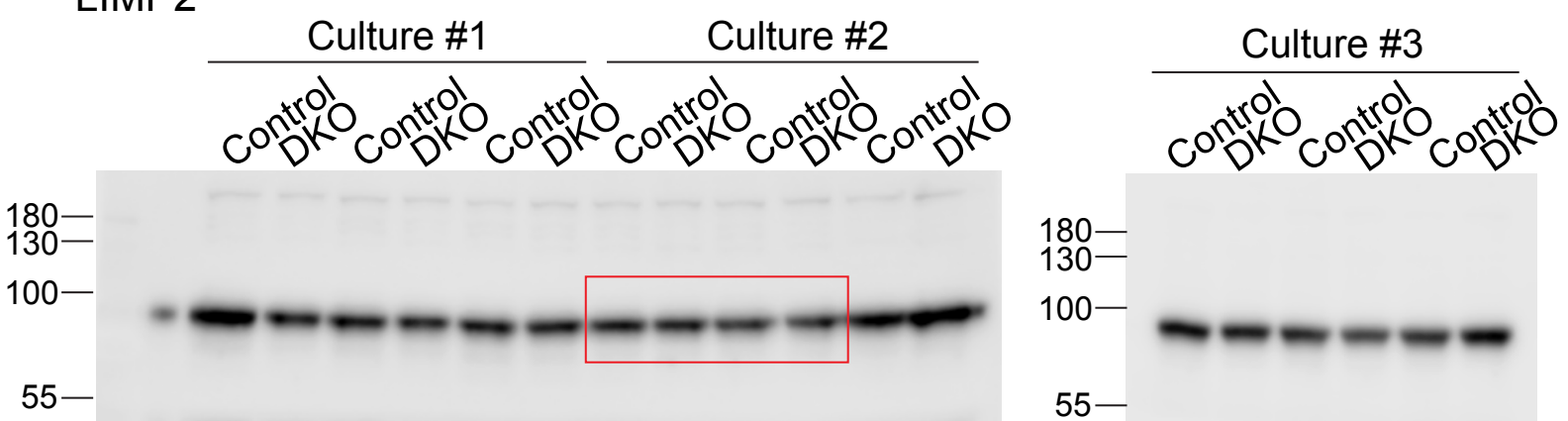

## Actin

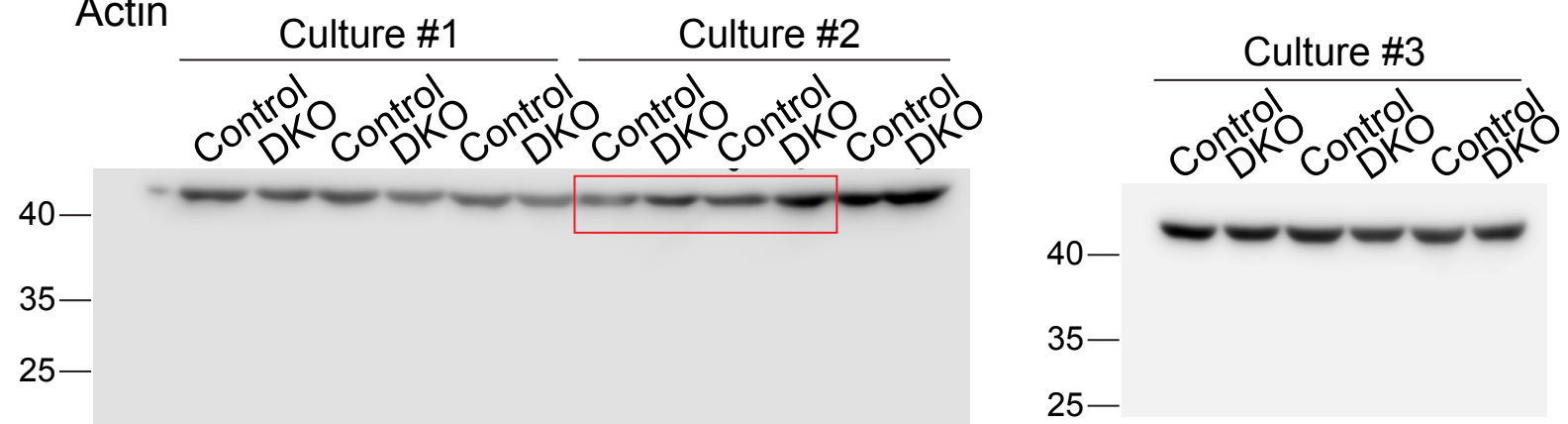

Supplement: Figure 2—figure supplement 3—source data 1. [file elife-85561-fig2-figsupp3-data1.zip › Figure 2 - figure supplement 3_source files/Figure 2 - figure supplement 3_uncropped blots.pdf]

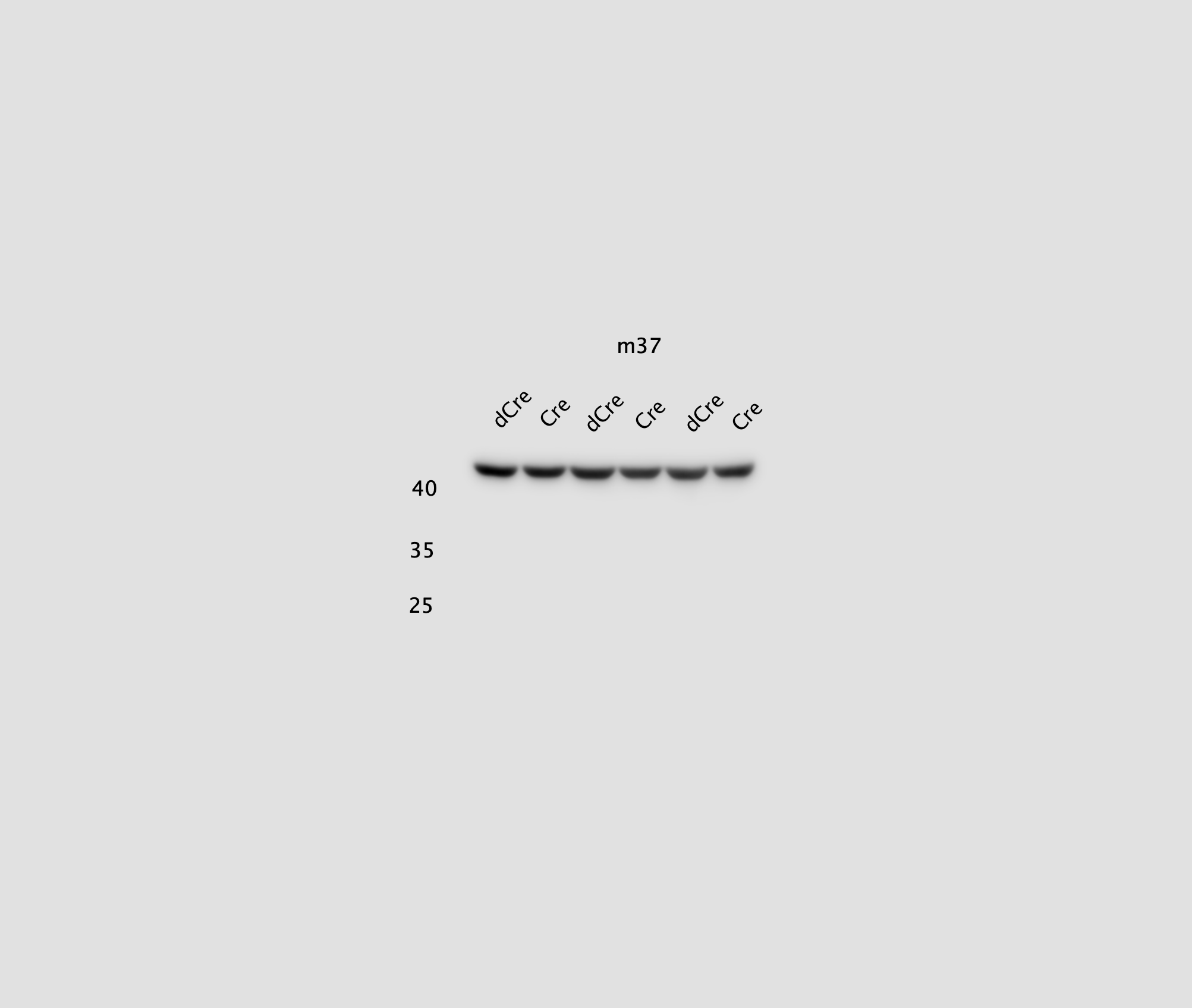

Supplement: Figure 2—figure supplement 3—source data 1. [file elife-85561-fig2-figsupp3-data1.zip › Figure 2 - figure supplement 3_source files/actin_replicate3.tif]

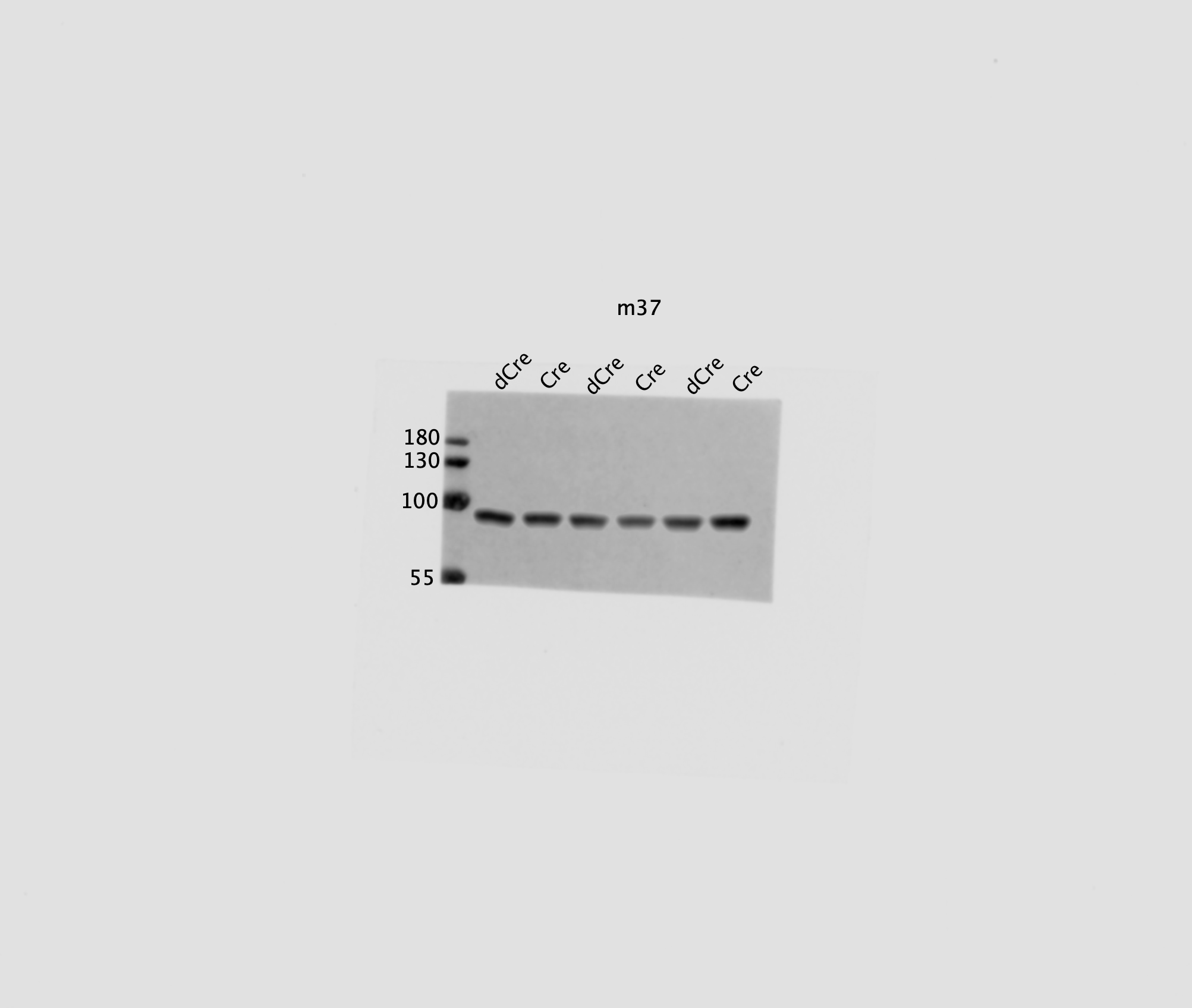

Supplement: Figure 2—figure supplement 3—source data 1. [file elife-85561-fig2-figsupp3-data1.zip › Figure 2 - figure supplement 3_source files/Limp2_replicate3_ladder.tif]

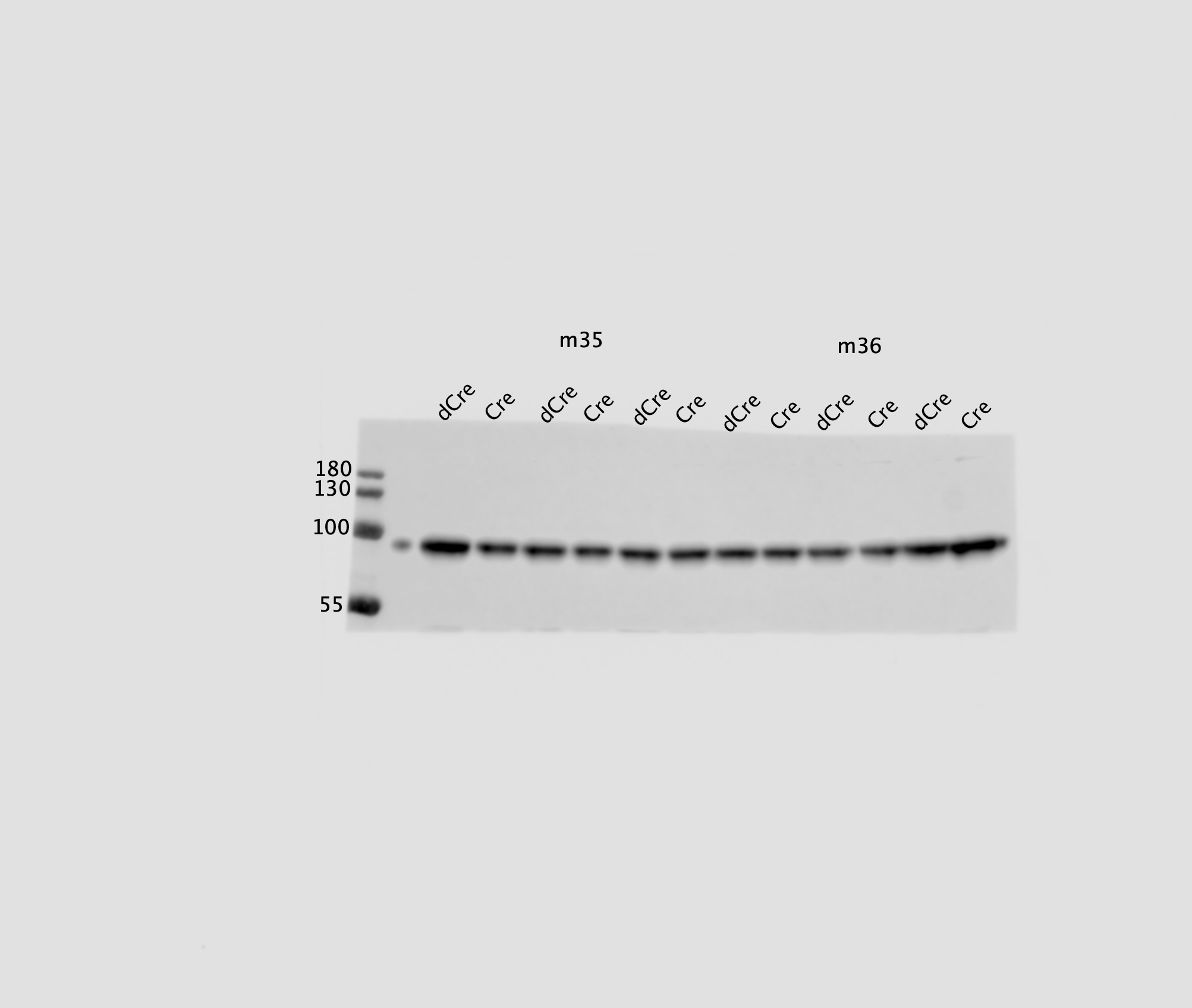

Supplement: Figure 2—figure supplement 3—source data 1. [file elife-85561-fig2-figsupp3-data1.zip › Figure 2 - figure supplement 3_source files/Limp2_replicates1-2_ladder.tif]

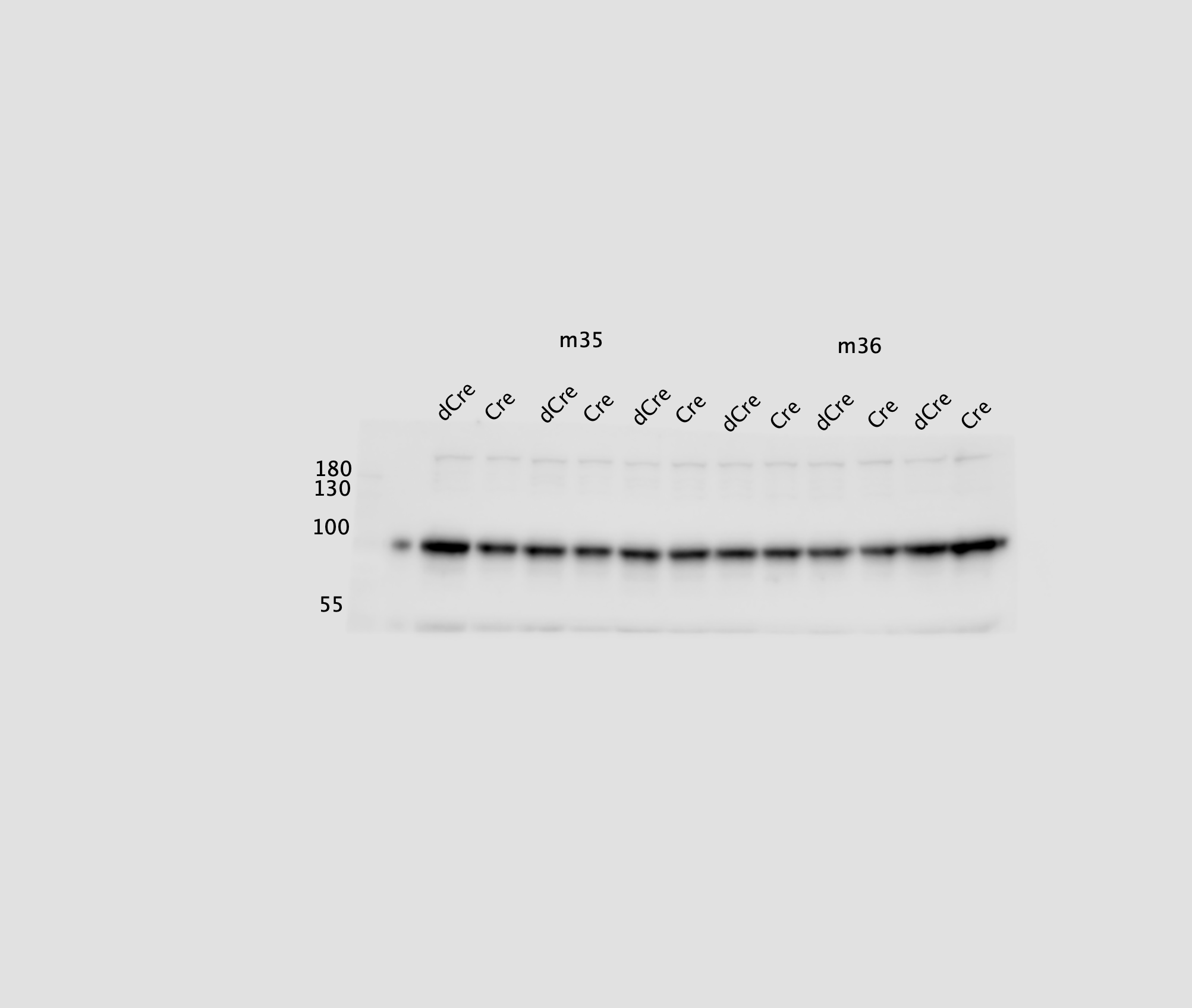

Supplement: Figure 2—figure supplement 3—source data 1. [file elife-85561-fig2-figsupp3-data1.zip › Figure 2 - figure supplement 3_source files/Limp2_replicates1-2.tif]

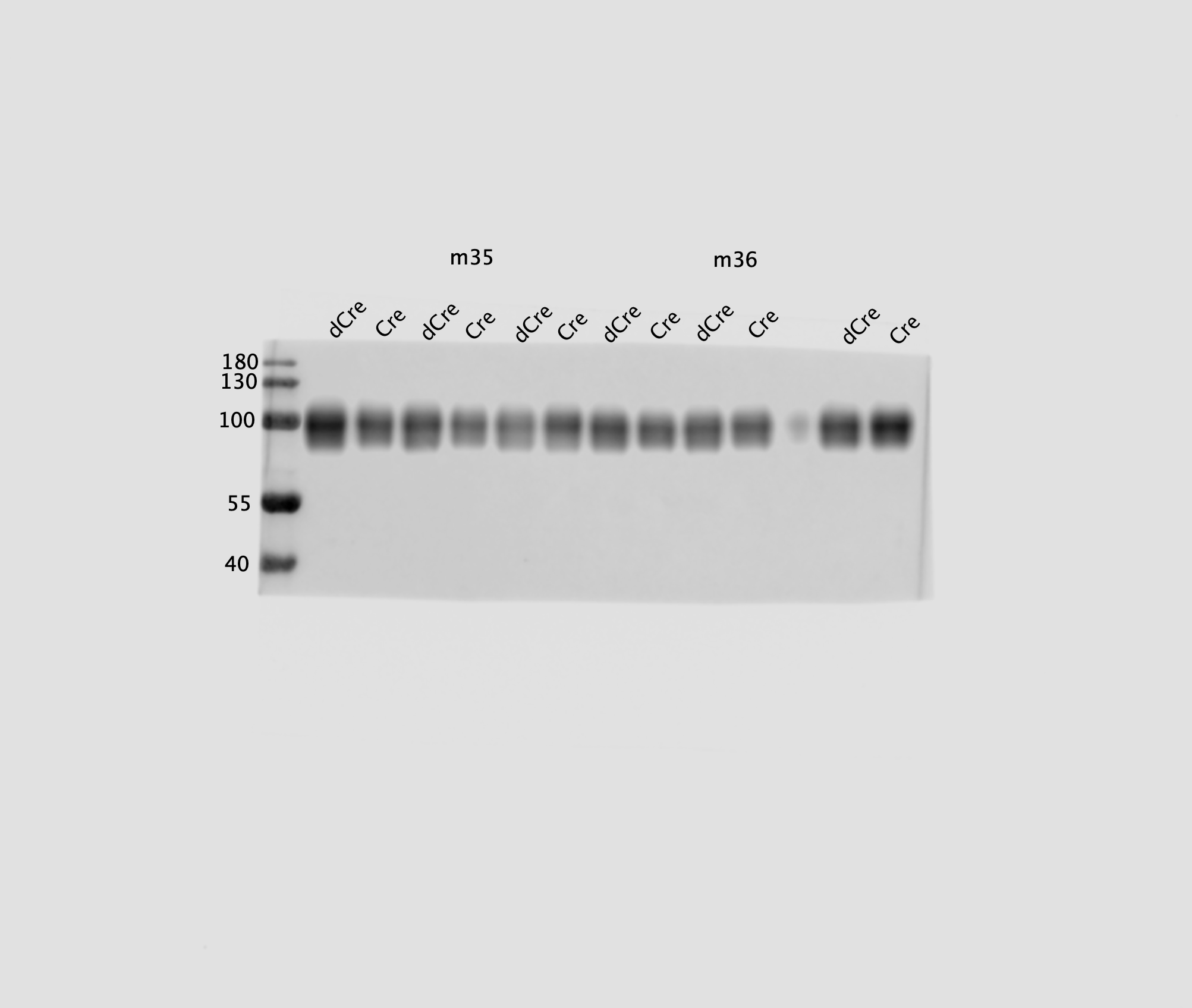

Supplement: Figure 2—figure supplement 3—source data 1. [file elife-85561-fig2-figsupp3-data1.zip › Figure 2 - figure supplement 3_source files/Lamp1_replicates1-2_ladder.tif]

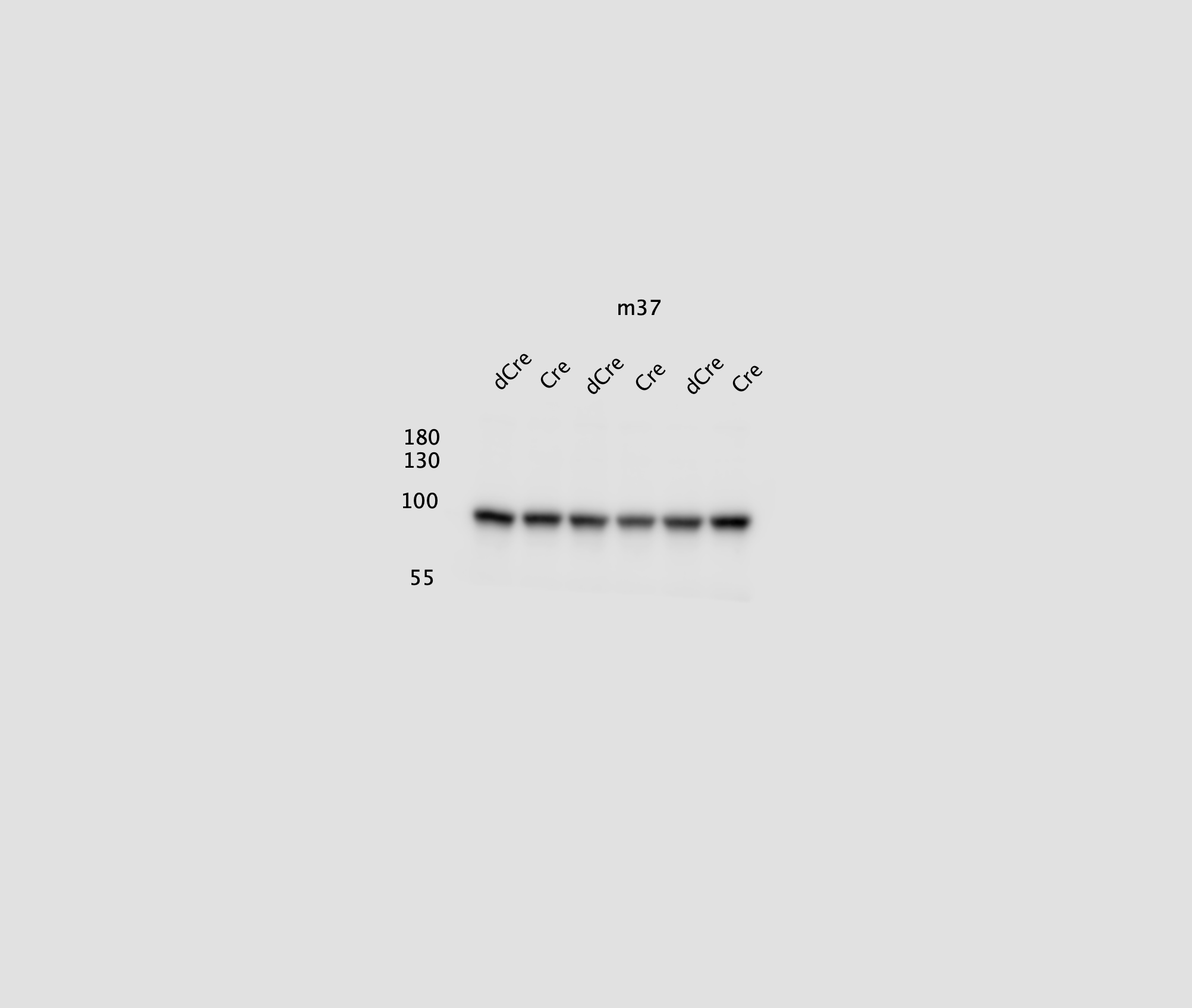

Supplement: Figure 2—figure supplement 3—source data 1. [file elife-85561-fig2-figsupp3-data1.zip › Figure 2 - figure supplement 3_source files/Limp2_replicate3.tif]

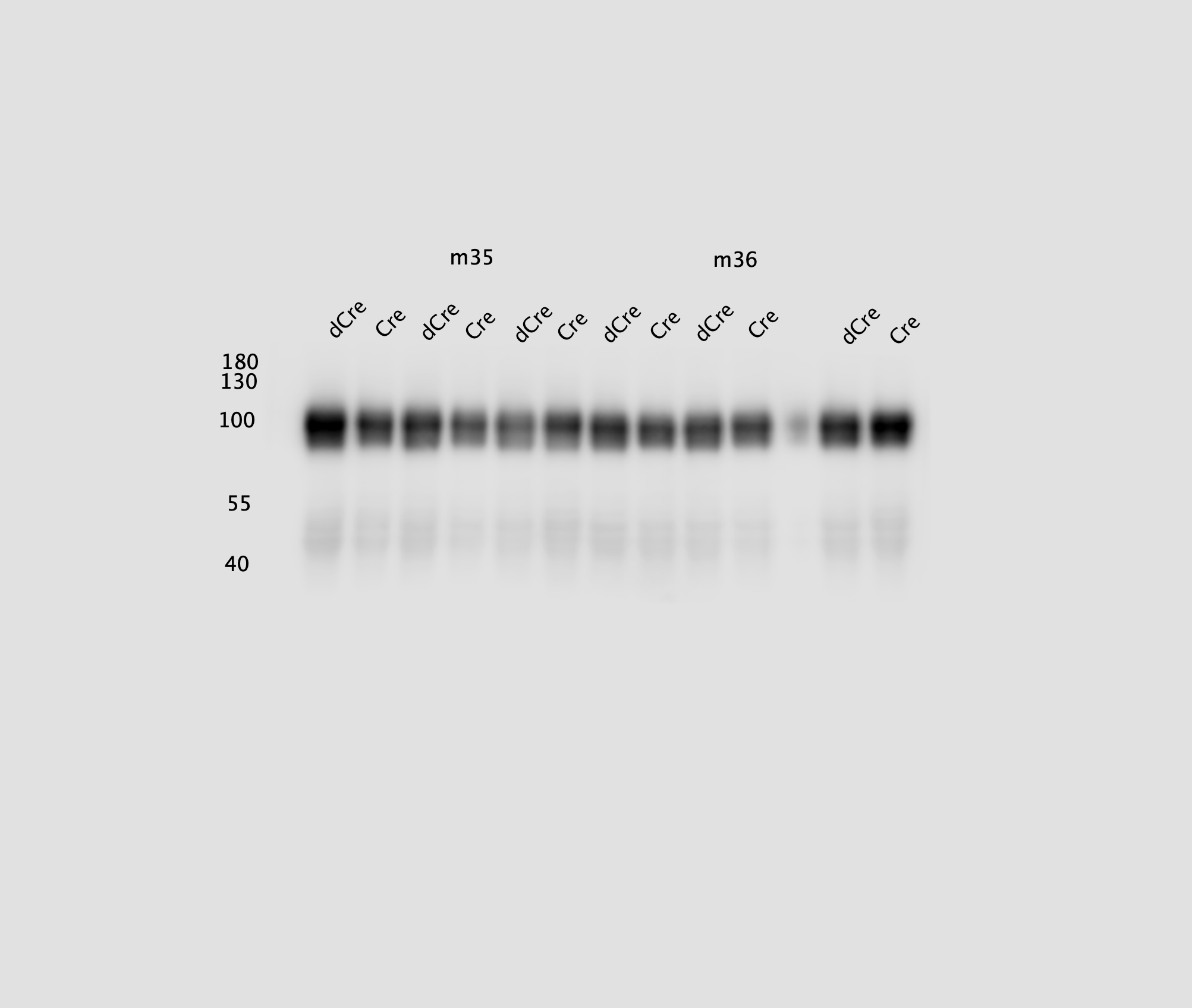

Supplement: Figure 2—figure supplement 3—source data 1. [file elife-85561-fig2-figsupp3-data1.zip › Figure 2 - figure supplement 3_source files/Lamp1_replicate1-2.tif]

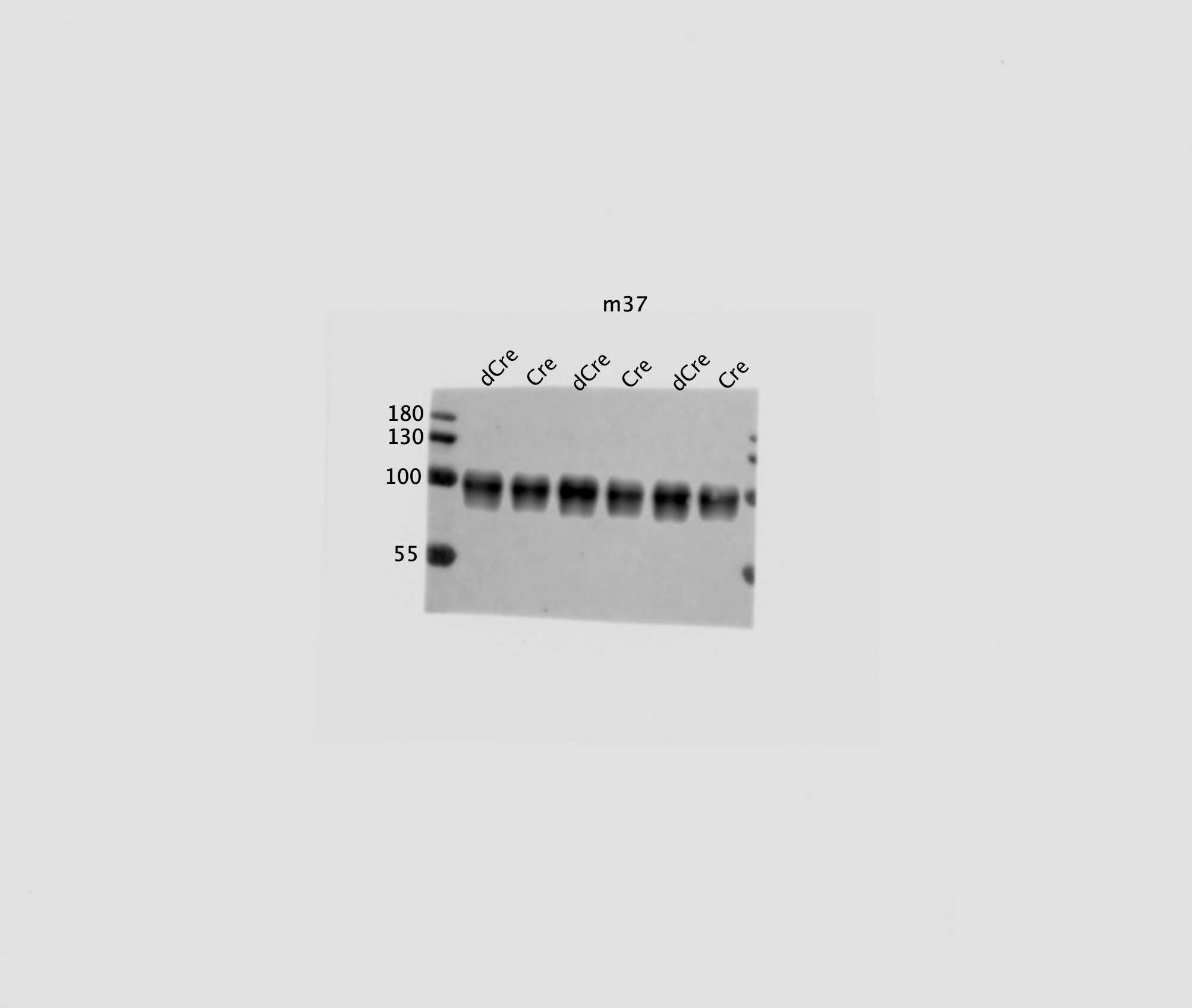

Supplement: Figure 2—figure supplement 3—source data 1. [file elife-85561-fig2-figsupp3-data1.zip › Figure 2 - figure supplement 3_source files/Lamp1_replicate3_ladder.tif]

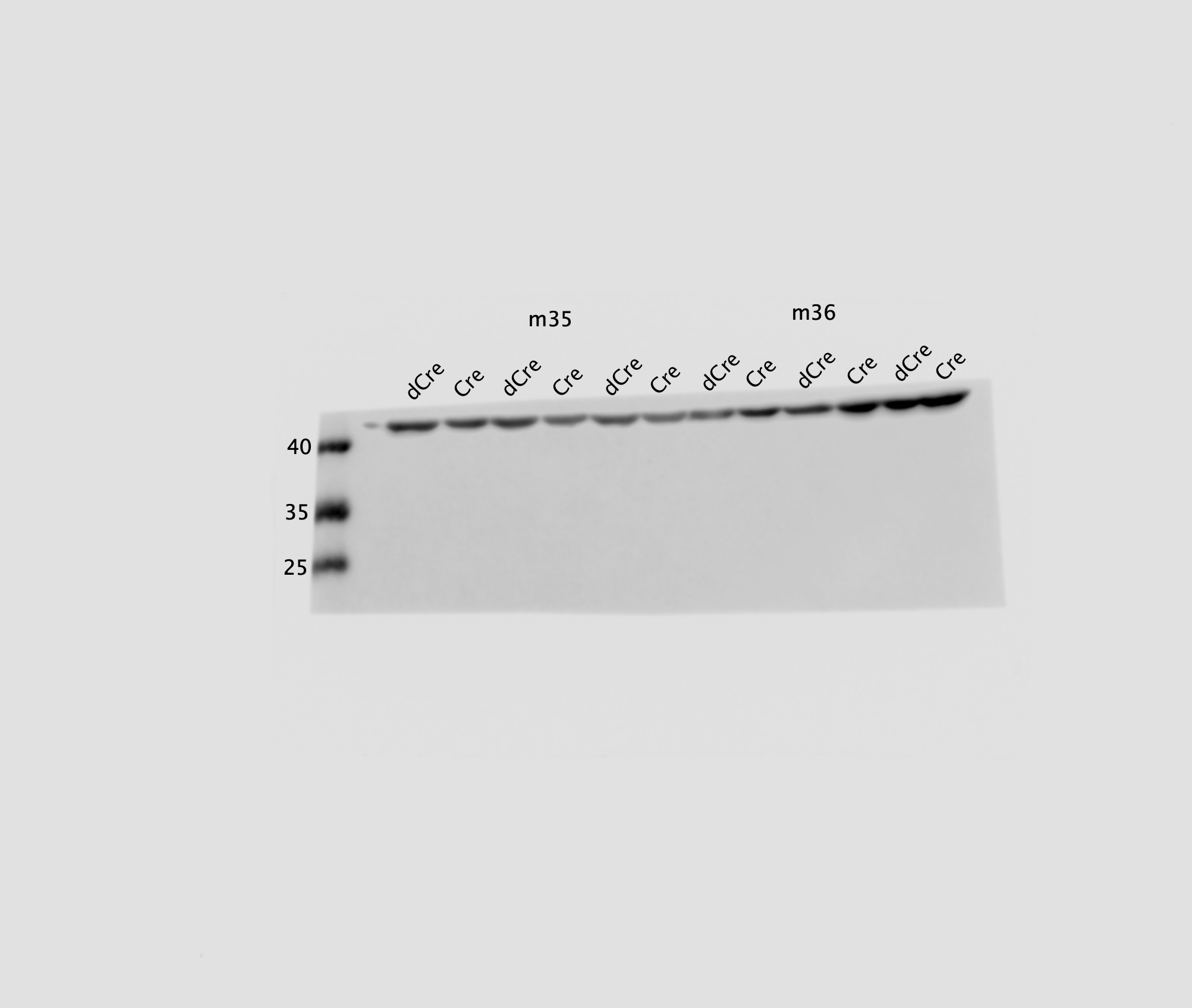

Supplement: Figure 2—figure supplement 3—source data 1. [file elife-85561-fig2-figsupp3-data1.zip › Figure 2 - figure supplement 3_source files/actin_replicates1-2_ladder.tif]

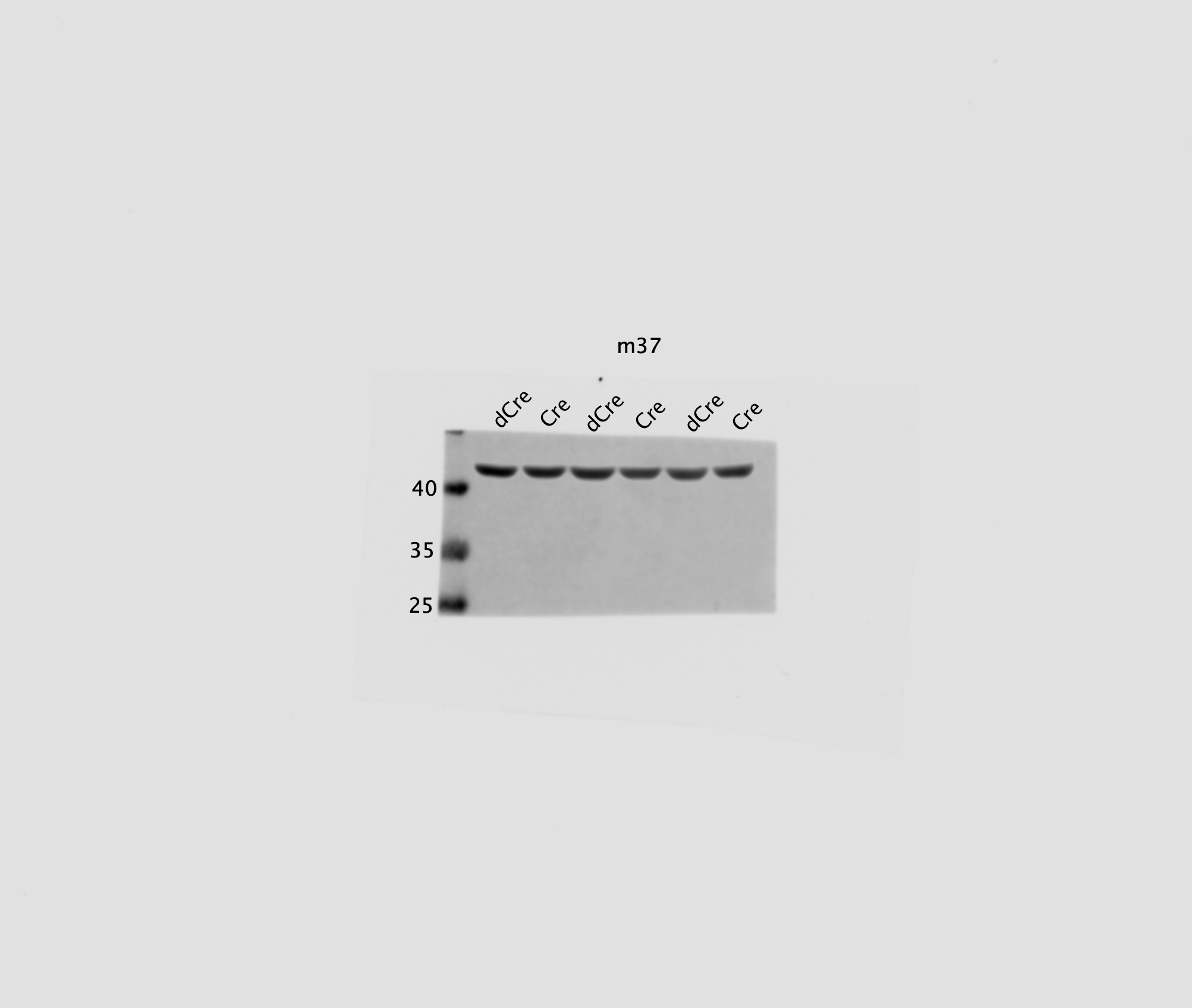

Supplement: Figure 2—figure supplement 3—source data 1. [file elife-85561-fig2-figsupp3-data1.zip › Figure 2 - figure supplement 3_source files/actin_replicate3_ladder.tif]

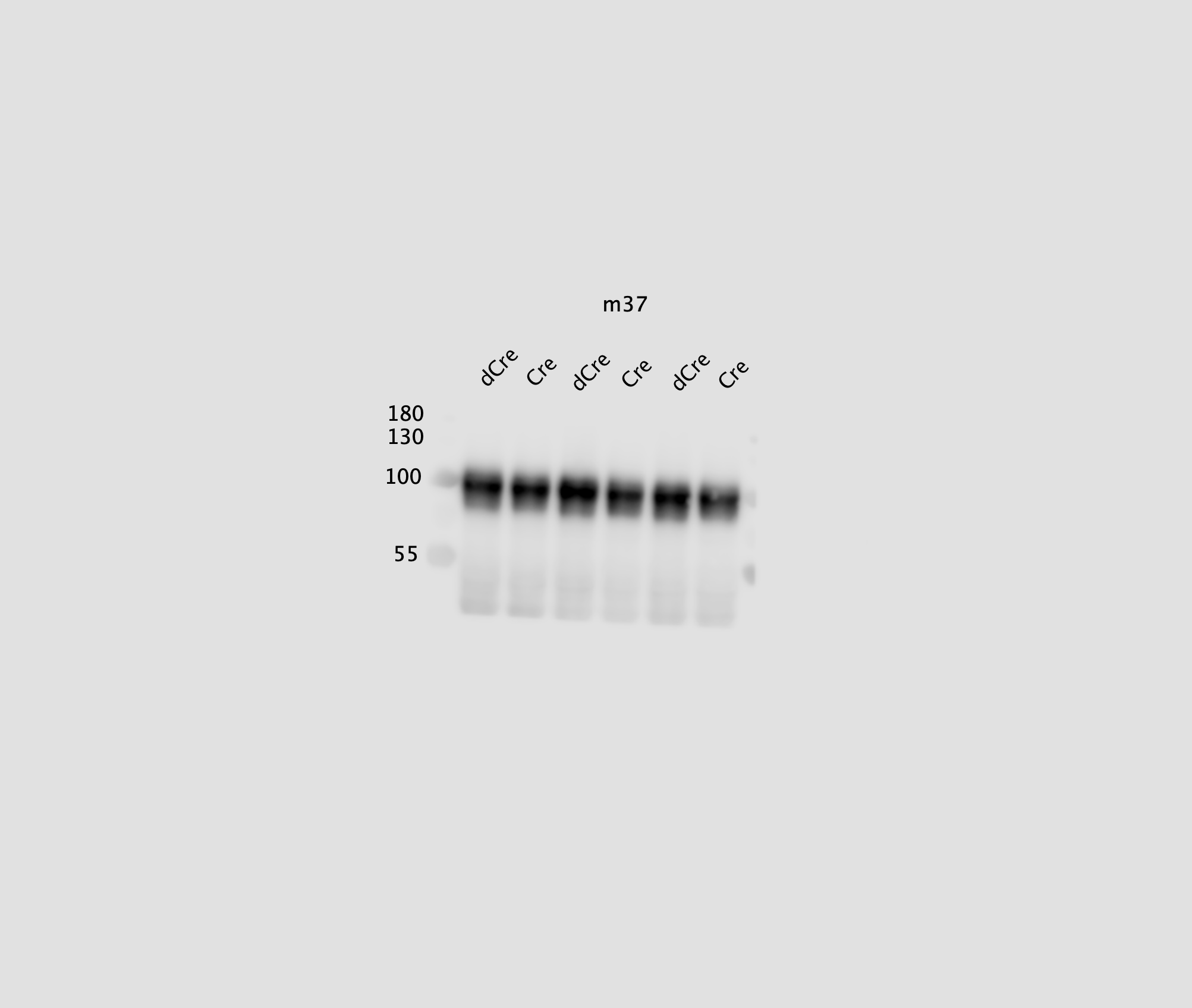

Supplement: Figure 2—figure supplement 3—source data 1. [file elife-85561-fig2-figsupp3-data1.zip › Figure 2 - figure supplement 3_source files/Lamp1_replicate3.tif]

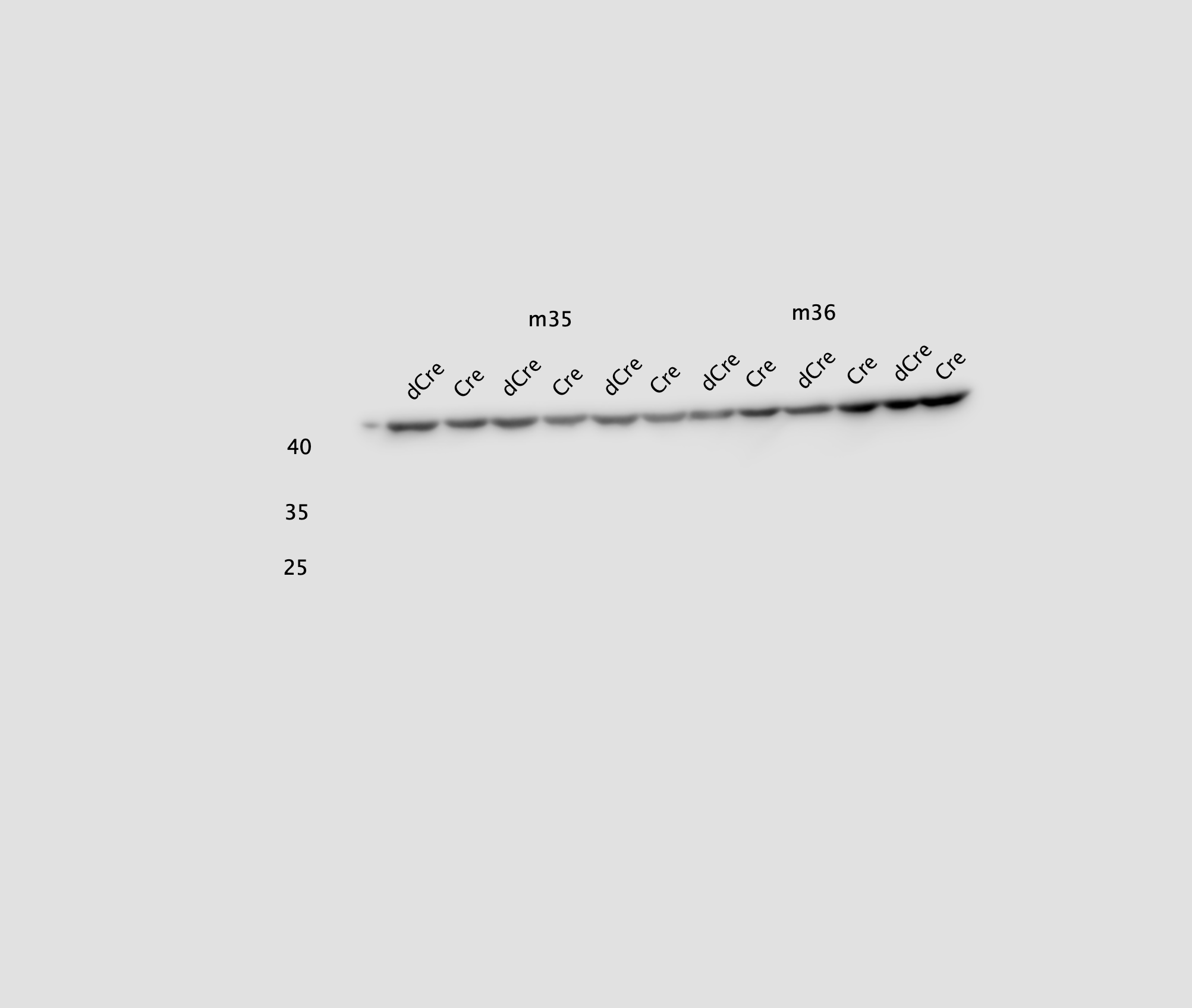

Supplement: Figure 2—figure supplement 3—source data 1. [file elife-85561-fig2-figsupp3-data1.zip › Figure 2 - figure supplement 3_source files/actin_replicates1-2.tif]

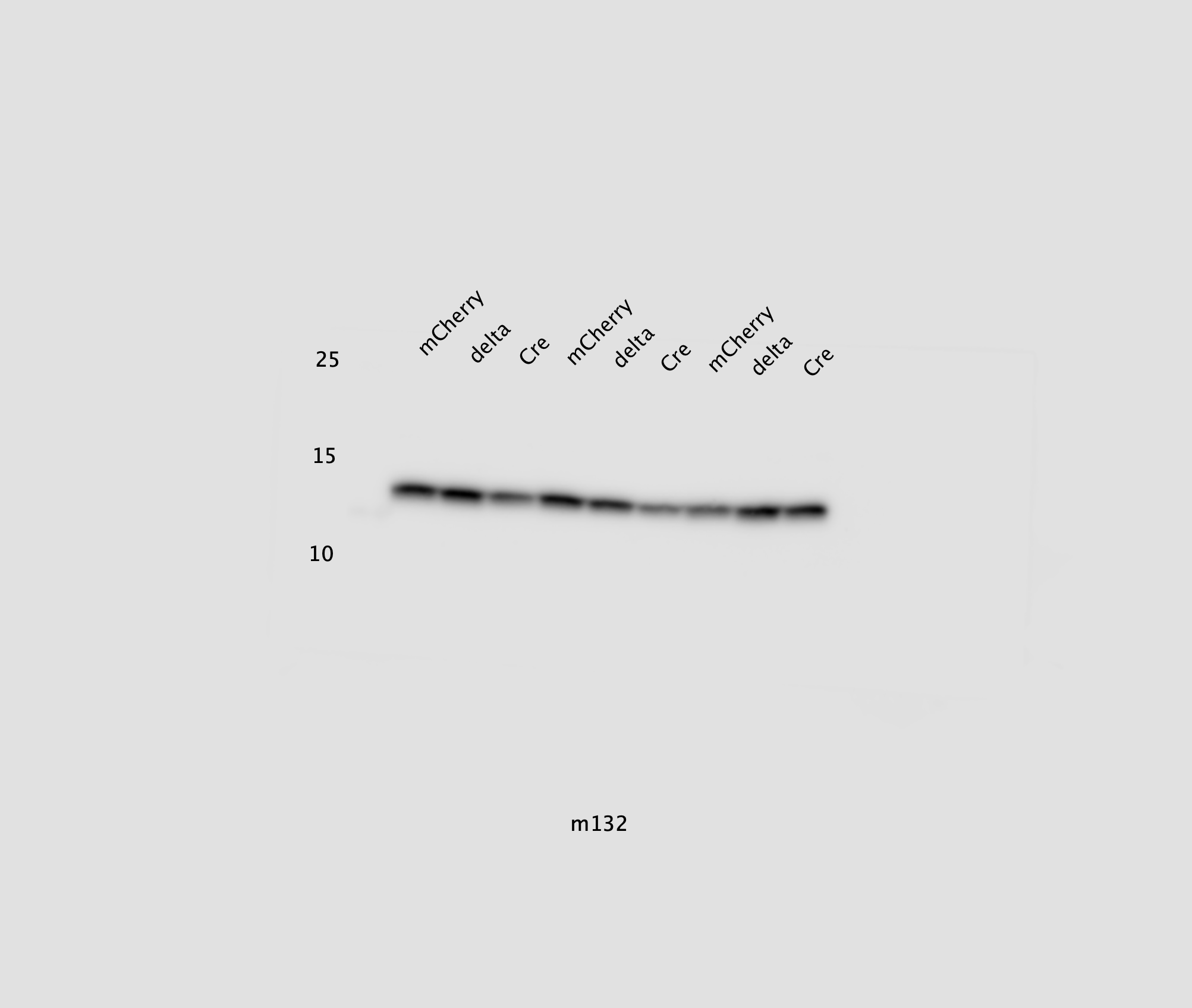

Supplement: Figure 6—source data 1. [file elife-85561-fig6-data1.zip › Figure 6_source files/BDNF.tif]

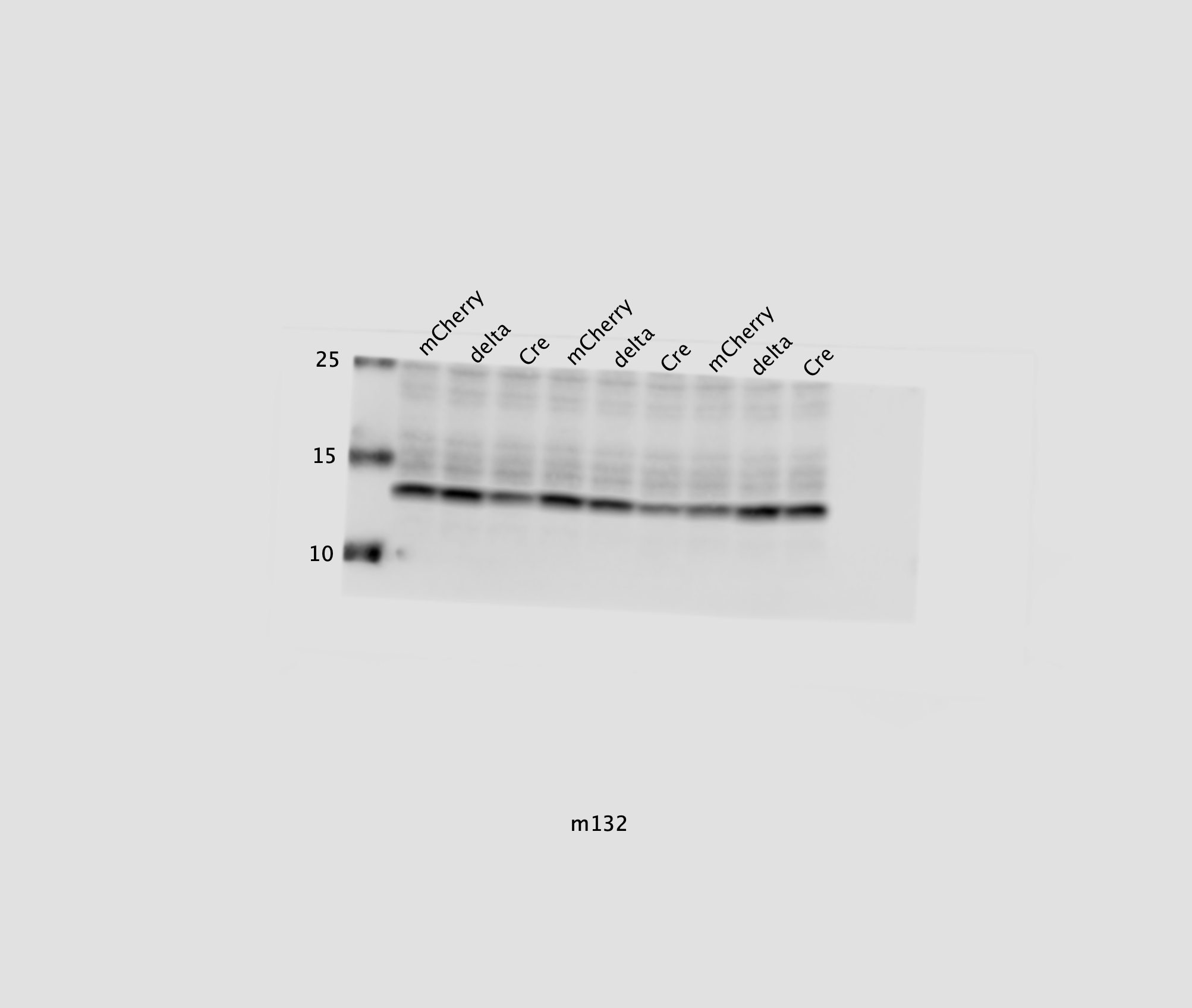

Supplement: Figure 6—source data 1. [file elife-85561-fig6-data1.zip › Figure 6_source files/BDNF_ladder.tif]

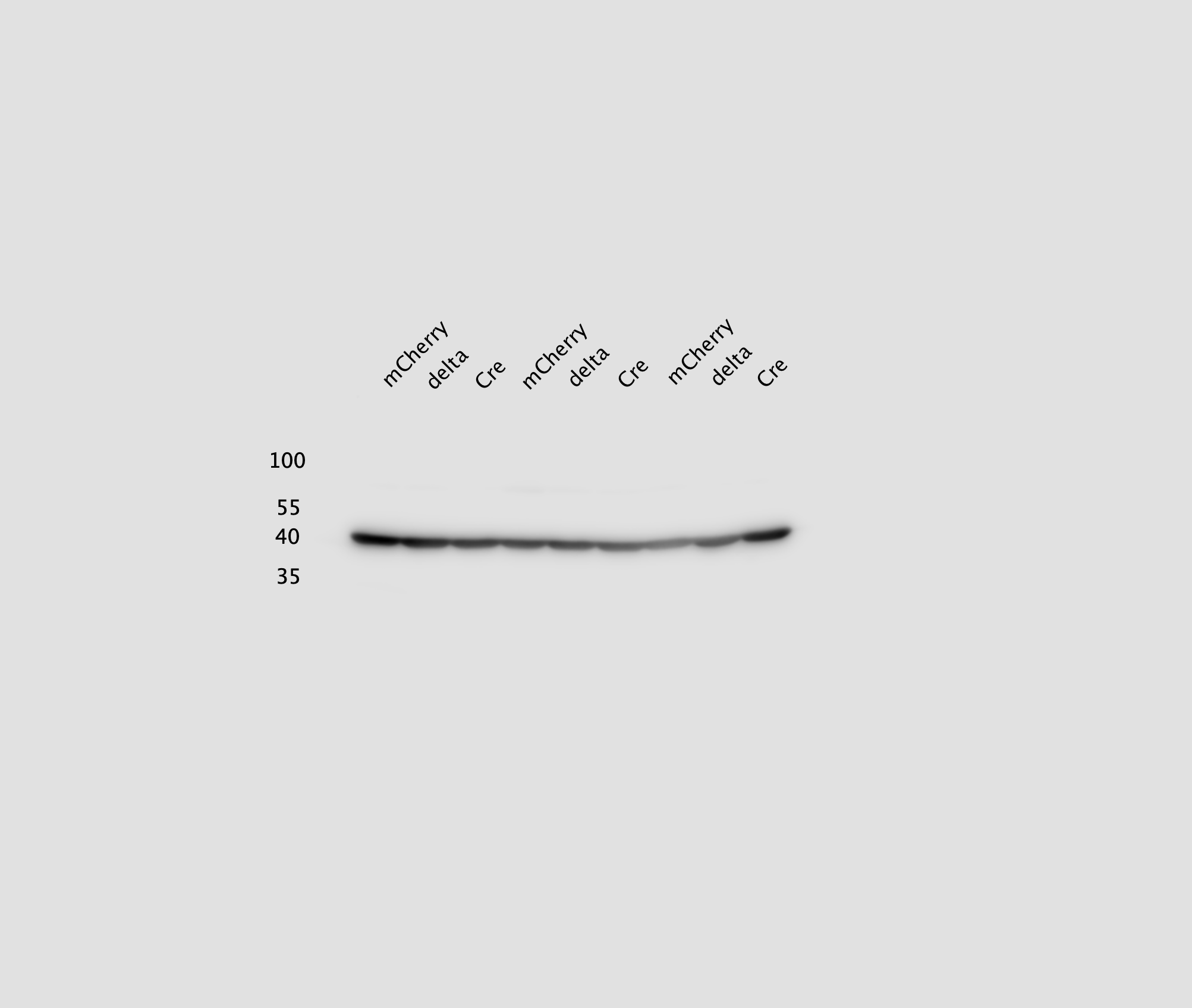

Supplement: Figure 6—source data 1. [file elife-85561-fig6-data1.zip › Figure 6_source files/actin.tif]

BDNF

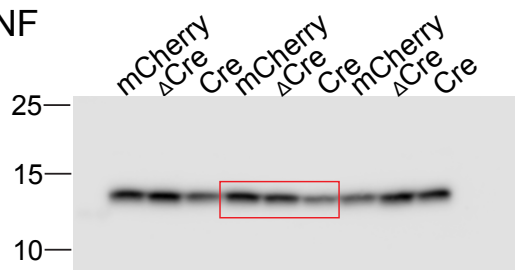

actin

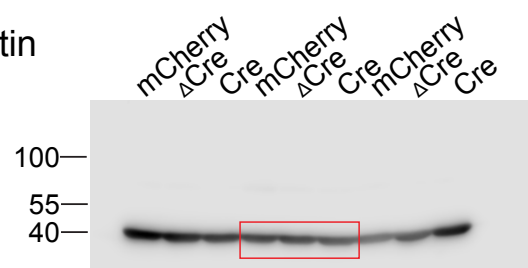

tomosyn

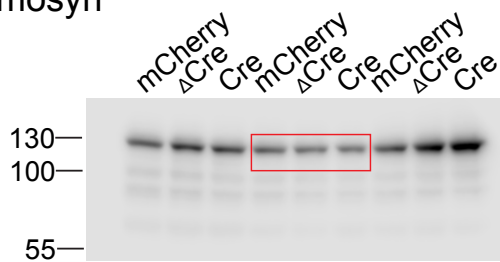

Supplement: Figure 6—source data 1. [file elife-85561-fig6-data1.zip › Figure 6_source files/Figure 6_source files.pdf]

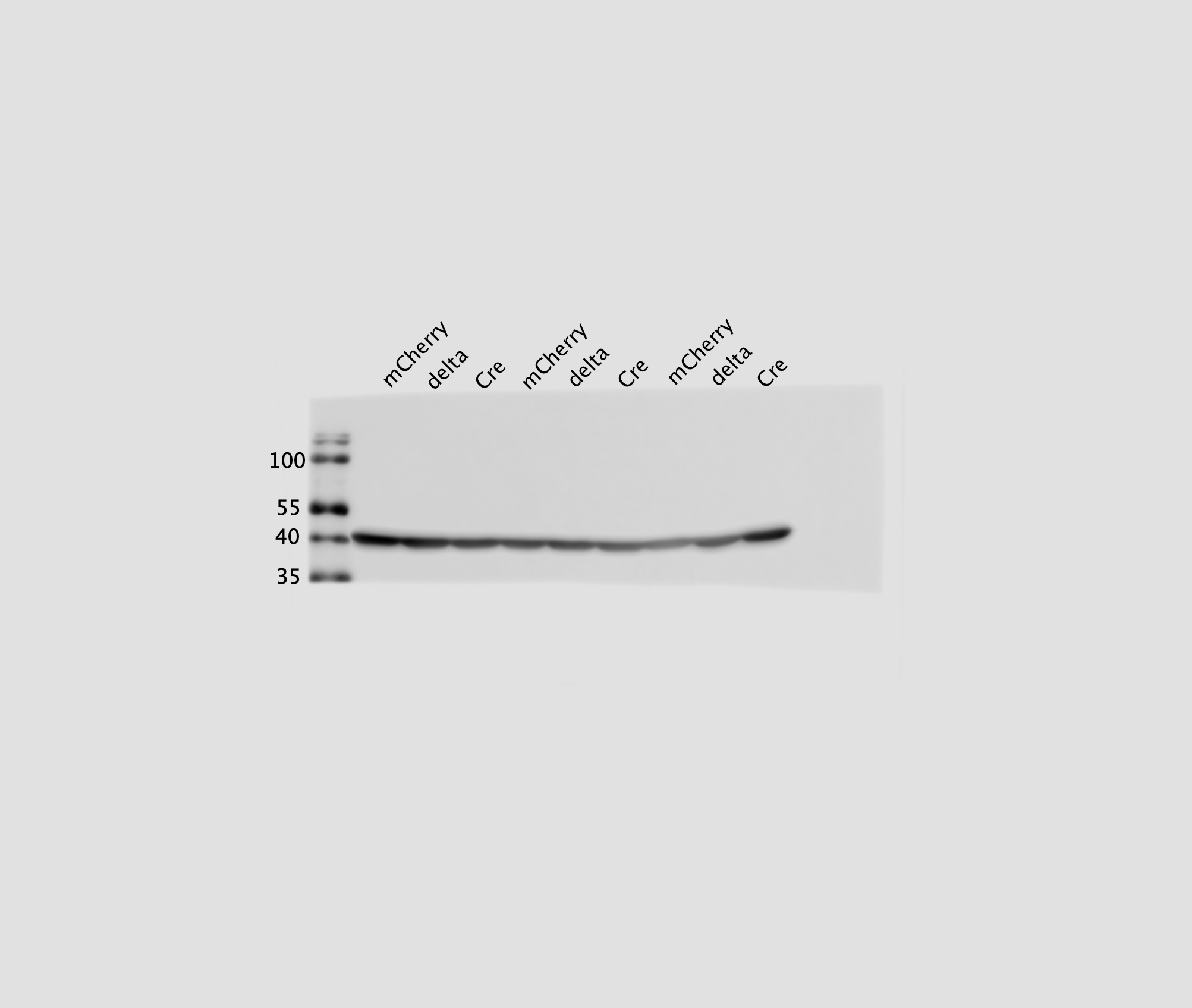

Supplement: Figure 6—source data 1. [file elife-85561-fig6-data1.zip › Figure 6_source files/actin_ladder.tif]

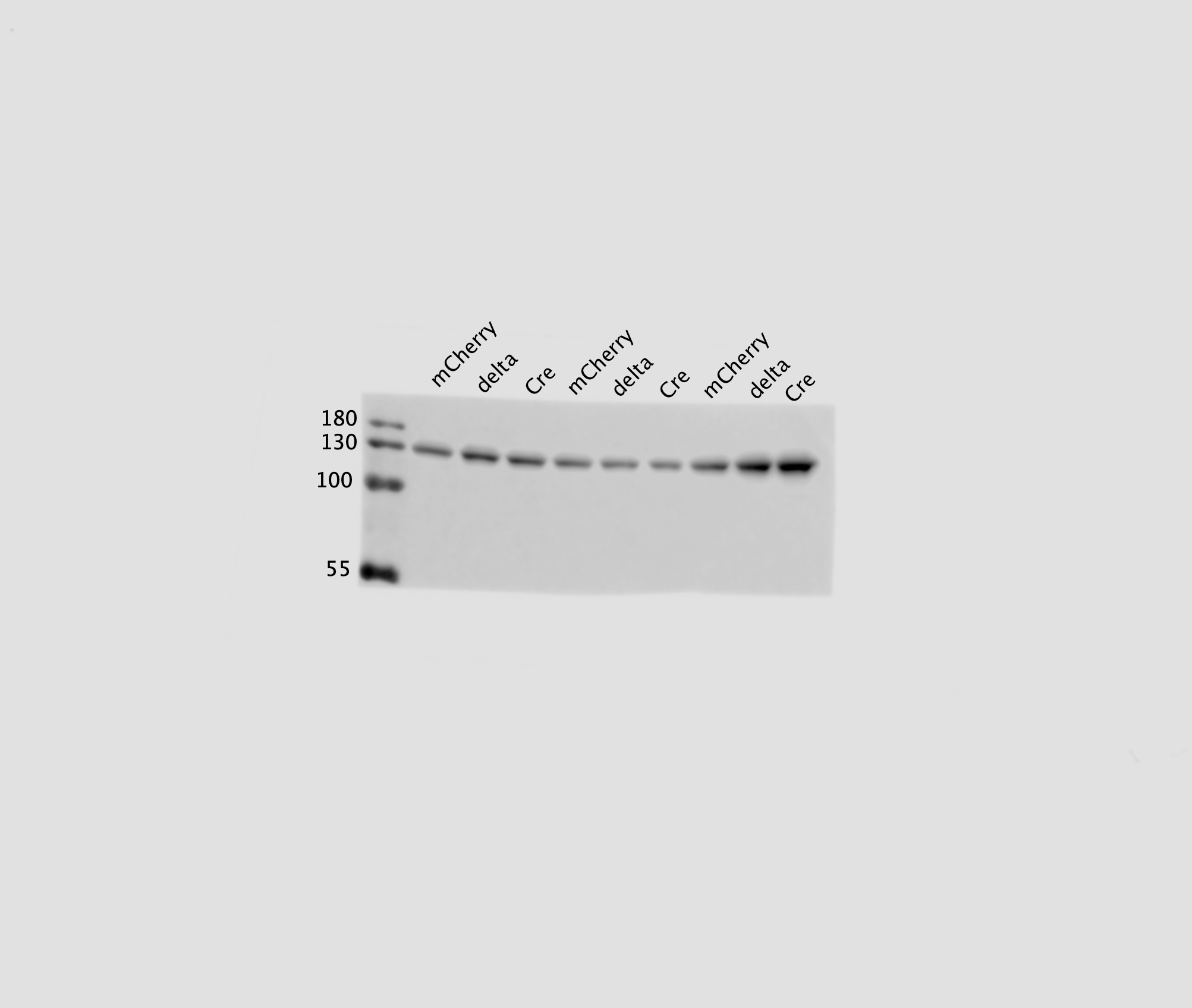

Supplement: Figure 6—source data 1. [file elife-85561-fig6-data1.zip › Figure 6_source files/tomosyn_ladder.tif]

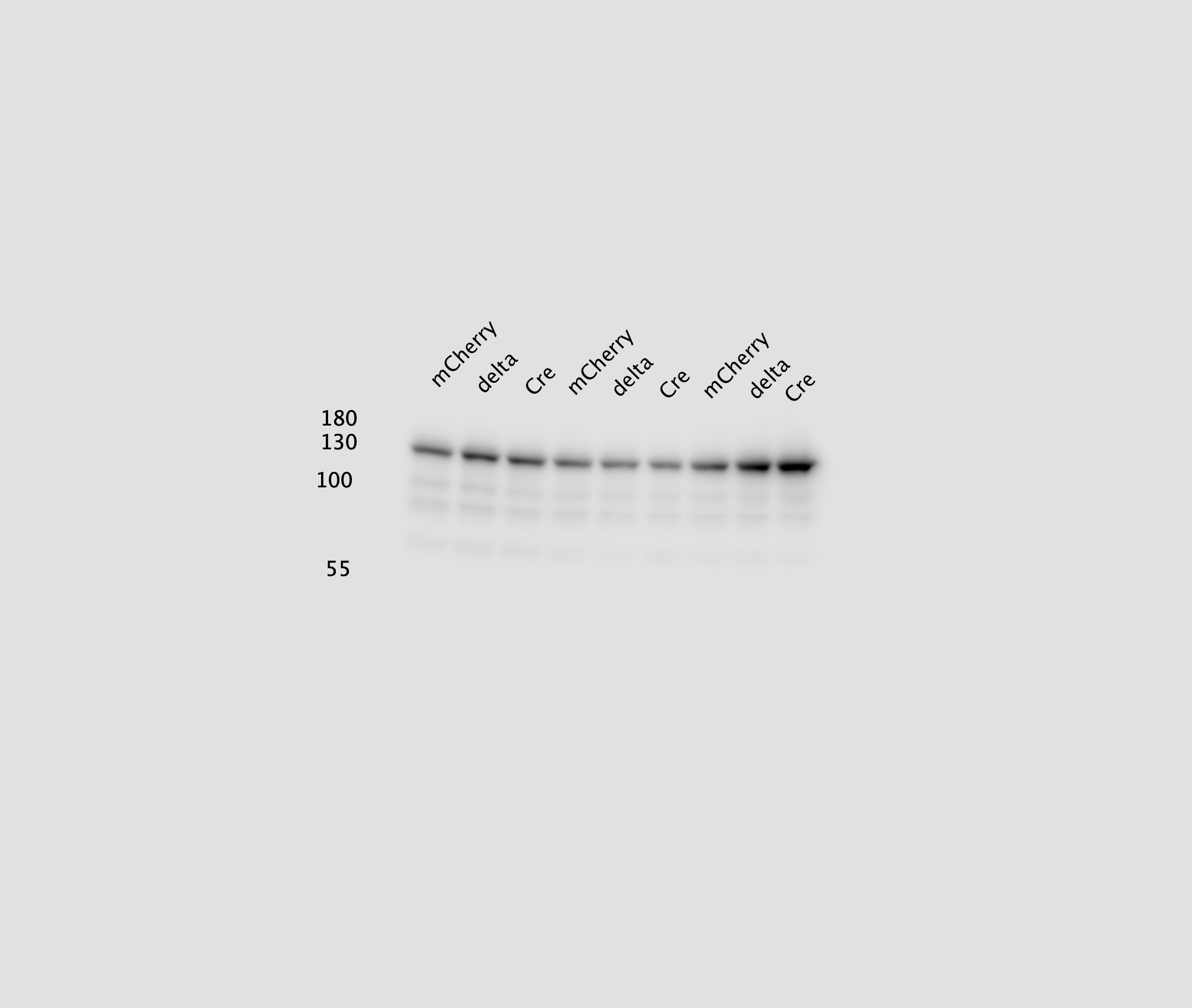

Supplement: Figure 6—source data 1. [file elife-85561-fig6-data1.zip › Figure 6_source files/tomosyn.tif]
